# Supplementary material for: Metabolic adaptations in cancers expressing isocitrate dehydrogenase mutations
Source: Cell Rep Med. 2021 Dec 21;2(12):100469. doi: 10.1016/j.xcrm.2021.100469 (PMC8714851; doi:10.1016/j.xcrm.2021.100469)
Supplement: Document S1. Figure S1 [file mmc1.pdf]

Cell Reports Medicine, Volume *2*

Supplemental information

Metabolic adaptations in cancers expressing isocitrate dehydrogenase mutations

Ingvild Comfort Hvinden, Tom Cadoux-Hudson, Christopher J. Schofield, and James S.O. McCullagh

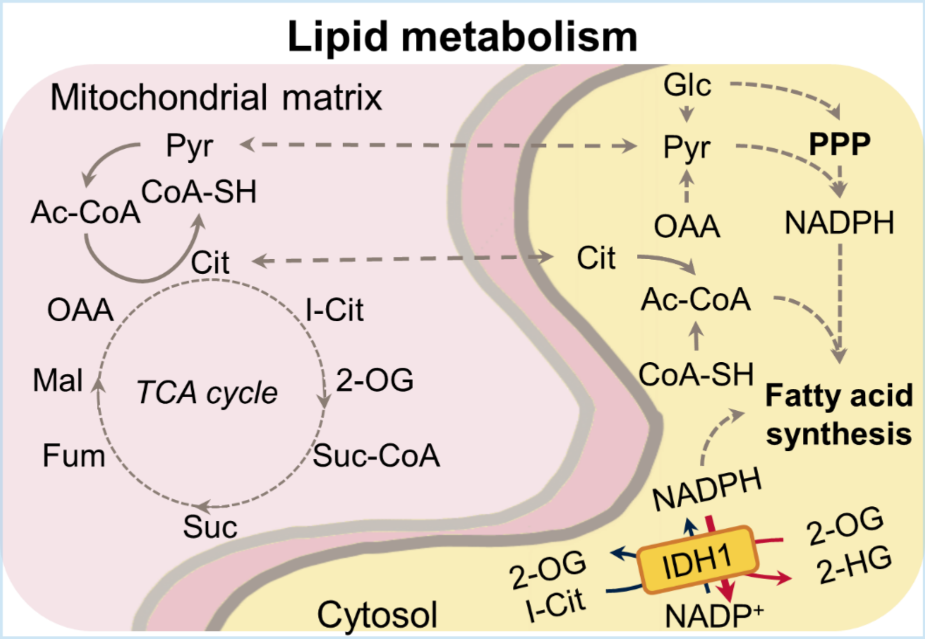

SI Figure 1. Overview of IDH related lipid biosynthesis. Lipid biosynthesis require NADPH, which is sourced from the oxidative pentose phosphate pathway (PPP), citric acid shuttle, and IDH1. When IDH1 is mutated, copious NADPH is used to synthesise 2-HG. Abbreviations: Cit (citrate), I-Cit (isocitrate), 2-OG (2-oxoglutarate), Suc-CoA (succinyl-CoA), Suc (succinate), Fum (fumarate), Mal (malate), OAA (oxaloacetate), Pyr (Pyruvate) and Ac-CoA (acetyl-CoA).
