# Supplementary material for: Metabolic adaptations in cancers expressing isocitrate dehydrogenase mutations
Source: Cell Rep Med. 2021 Dec 21;2(12):100469. doi: 10.1016/j.xcrm.2021.100469 (PMC8714851; doi:10.1016/j.xcrm.2021.100469)
Supplement: Document S2. Article plus supplemental information [file mmc2.pdf]

Cell Reports Medicine

CellPress
OPEN ACCESS

Review

Metabolic adaptations in cancers expressing isocitrate dehydrogenase mutations

Ingvild Comfort Hvinden,[1] Tom Cadoux-Hudson,[1] Christopher J. Schofield,[1,2] and James S.O. McCullagh[1,*]
[1]Chemistry Research Laboratory, 12 Mansfield Road, Department of Chemistry, University of Oxford, Oxford OX1 3TA, UK
[2]Ineos Oxford Institute for Antimicrobial Research, 12 Mansfield Road, Department of Chemistry, University of Oxford, Oxford OX1 3TA, UK
*Correspondence: james.mccullagh@chem.ox.ac.uk
https://doi.org/10.1016/j.xcrm.2021.100469

SUMMARY

The most frequently mutated metabolic genes in human cancer are those encoding the enzymes isocitrate dehydrogenase 1 (IDH1) and IDH2; these mutations have so far been identified in more than 20 tumor types. Since *IDH* mutations were first reported in glioma over a decade ago, extensive research has revealed their association with altered cellular processes. Mutations in *IDH* lead to a change in enzyme function, enabling efficient conversion of 2-oxoglutarate to *R*-2-hydroxyglutarate (*R*-2-HG). It is proposed that elevated cellular *R*-2-HG inhibits enzymes that regulate transcription and metabolism, subsequently affecting nuclear, cytoplasmic, and mitochondrial biochemistry. The significance of these biochemical changes for tumorigenesis and potential for therapeutic exploitation remains unclear. Here we comprehensively review reported direct and indirect metabolic changes linked to *IDH* mutations and discuss their clinical significance. We also review the metabolic effects of first-generation mutant IDH inhibitors and highlight the potential for combination treatment strategies and new metabolic targets.

INTRODUCTION

Metabolic alterations are a hallmark of cancer, but their role in tumorigenesis is not well understood.[1,2] Mutations in the genes encoding enzymes linked to central carbon metabolism have been found in some cancers, including enzymes such as isocitrate dehydrogenase (IDH), succinate dehydrogenase (SDH),[3] and fumarate hydratase (FH)[4]. *SDH* and *FH* mutations are apparently loss-of-function mutations, causing succinate and fumarate, respectively, to accumulate to abnormally high levels, leading to a range of subsequent intracellular metabolic changes.[3,4] Early reports suggested that cancer-associated *IDH1* mutations also caused a "simple" loss of the ability to catalyze conversion of isocitrate to 2-oxoglutarate (2-OG),[5] also known as α-ketoglutarate, and that wild-type (WT) IDH1 activity was dominantly inhibited by formination of a heterodimer with mutant IDH1 (mutIDH1).[6] In a seminal study, Dang et al.[7] revealed that mutIDH1^{R132H} catalyzes production of the metabolite *R*-2-hydroxyglutarate (*R*-2-HG), also referred to as D-2-HG, showing apparent oncogenic selection for production of a specific metabolite. Soon thereafter it was demonstrated that mutIDH2^{R172K} and mutIDH2^{R140Q} also catalyze enantioselective production of *R*-2-HG.[8] The *R*- and *S*-2-HG enantiomers are present at low micromolar levels in healthy individuals,[9–11] but their roles in normal metabolism are poorly understood. For the common mutations of *IDH1* and *IDH2* found in cancer, intracellular and extracellular *R*-2-HG levels are substantially increased.[7,8] *R*-2-HG is now one of the best-validated small-molecule biomarkers in cancer and has been shown to have considerable diagnostic potential.[7,12]

Mutations in the genes for *IDH1* and *IDH2* have now been identified in more than 20 different neoplasms (Table 1). They are prevalent in grade II and III gliomas (>70%) and secondary glioblastomas (GBMs) (55%–88%) but not primary GBMs (5%–14%).[5,13–18] The *IDH* mutations are also prevalent in certain cartilaginous and bone tumors (20%–80%),[19–26] acute myeloid leukemia (AML) (15%–30%),[8,27–34] intrahepatic cholangiocarcinoma (ICC) (6%–30%),[35–43] angioimmunoblastic T cell lymphoma (20%–30%),[44–47] sinonasal undifferentiated carcinoma (35%–80%),[48–50] and solid papillary carcinoma with reverse polarity (>77%).[51,52] The importance of *IDH1/2* mutations in glioma is reflected by the fact that, since 2016, they have featured as diagnostic criteria in the World Health Organization's (WHO) categorization of central nervous system (CNS) tumors.[53] The updated 2021 WHO classification of CNS tumors further emphasizes the clinical importance of the *IDH1/2* mutations by reducing the number of types of adult diffuse glioma to three (astrocytoma, oligodendroglioma, and GBM), with astrocytoma and oligodendroglioma now requiring the presence of an *IDH1/2* mutation for diagnosis.[54] In the remaining cancer types in which *IDH1* or *IDH2* mutations are reported, the incidence rates are lower (<5%). Interestingly, with rare exceptions,[15,28,34] mutations of *IDH1* and *IDH2* appear to be mutually exclusive.[15,18,33]

Mutation of *IDH1* and *IDH2* are reported to occur early in development of solid tumor cells[17,83] but not hematopoietic malignancies.[61,84,85] The current view is that, in nascent tumor cells, elevated *R*-2-HG may dysregulate multiple enzymes, including

This is an open access article under the CC BY license (http://creativecommons.org/licenses/by/4.0/).

Table 1. Reported frequency of canonical *IDH1* and *IDH2* mutations in cancers and benign tumors

Cancer type	Reported occurrence (%) mutIDH1 (R132)	mutIDH2 (R172 or R140)	Other mutIDH1/2	Source
CNS neoplasm				
Grade II and III glioma	>70	5	0.3–2.3	Yan et al.,[5] Balss et al.,[13] Parsons et al.,[14] Hartmann et al.,[15] Ichimura et al.,[16] Watanabe et al.,[17] Pusch et al.,[55] Gupta et al.[56]
Secondary GBM (grade IV)	55–88	3.4	–	Yan et al.,[5] Balss et al.,[13] Parsons et al.,[14] Watanabe et al.,[17] Wang et al.[18]
Primary GBM (grade IV)	5–14	0.5	–	Yan et al.,[5] Parsons et al.,[14] Hartmann et al.,[15] Ichimura et al.,[16] Watanabe et al.,[17] Wang et al.,[18] Balss et al.[57]
Myeloid and lymphoid neoplasms				
AML	6–13	8–20	0.6	Ward et al.,[8] Mardis et al.,[27] Abbas et al.,[28] Marcucci et al.,[29] Schnittger et al.,[30] Wagner et al.,[31] Molenaar et al.,[32] Figueroa et al.,[33] Paschka et al.,[34] Gross et al.[58]
B cell acute lymphoblastic leukemia	1.7	–	–	Kang et al.[59]
Angioimmunoblastic T cell lymphoma	–	20–33	–	Cairns et al.,[44] Odejide et al.,[45] Sakata-Yanagimoto et al.,[46] Wang et al.[47]
Peripheral T cell lymphoma	–	<5	–	Wang et al.[47]
Myelodysplastic syndrome	<4	<4	–	Molenaar et al.,[32] Thol et al.[60]
Myeloproliferative neoplasm, chronic or fibrotic phase	<3	<1.5	–	Tefferi et al.,[61] Pardanani et al.[62]
Myeloproliferative neoplasm, blast phase	5–12	2–9	–	Tefferi et al.,[61] Pardanani et al.[62]
Pediatric AML	<1.5	<2.5	–	Andersson et al.,[63] Oki et al.[64]
Pediatric acute lymphoblastic leukemia	0.4	0	–	Andersson et al.[63]
Bile duct neoplasms				
ICC	6.5–32	1–9	0.3	Borger et al.,[35] Kipp et al.,[36] Wang et al.,[37] Jiao et al.,[38] Ross et al.,[39] Farshidfar et al.,[40] Lee et al.,[41] Nepal et al.,[42] Wang et al.[43]
Extrahepatic cholangiocarcinoma/clear cell extrahepatic cholangiocarcinoma	0–10	<4	–	Borger et al.,[35] Kipp et al.,[36] Lee et al.,[41] Ally et al.[65]
Cartilage and bone neoplasms				
Chondrosarcoma	12–54	5–16	–	Amary et al.,[19] Arai et al.,[20] Lu et al.,[21] Jin et al.,[23] Lugowska et al.,[24] Cleven et al.,[25] Tallegas et al.,[26] Zhu et al.[66]
Giant-cell tumor of the bone/osteoclastoma	–	80	25	Kato Kaneko et al.[22]
Osteosarcoma	–	28	–	Liu et al.[67]
Ewing sarcoma family tumors	3.3	3.3	–	Na et al.[68]
Ollier disease- and Mafucci syndrome-related neoplasms				
Ollier disease-related enchondroma and chondrosarcomas	>80	3	–	Pensuriya et al.,[69] Amary et al.[70]
Mafucci syndrome-related enchondroma and chondrosarcomas	>80	–	–	Pensuriya et al.,[69] Amary et al.[70]
Mafucci syndrome-related hemangioma	1 reported case	–	–	Amary et al.[70]
Mafucci syndrome-related spindle cell hemangioma	70	–	–	Pensuriya et al.[69]
Other neoplasms				
Breast cancer (other)	0.2	–	–	Fathi et al.[71]
Solid papillary carcinoma with reverse polarity, rare breast cancer subtype	–	> 77	-	Chiang et al.,[51] Lozada et al.[52]

(*Continued on next page*)

CellPress
OPEN ACCESS

Table 1. *Continued*

	Reported occurrence (%)			
Cancer type	mutIDH1 (R132)	mutIDH2 (R172 or R140)	Other mutIDH1/2	Source
Gastric adenocarcinoma	2.7	–	–	Li-Chang et al.[72]
Irritable bowel syndrome-associated intestinal adenocarcinoma	13	–	–	Hartman et al.[73]
Melanoma metastasis	1.3	–	–	Lopez et al.[74]
Non-small cell lung cancer	0.6	0.4	–	Toth et al.[75]
Paraganglioma	1.5	–	–	Gaal et al.[76]
Prostate cancer	0.3–2.7	–	–	Kang et al.,[59] Hinsch et al.[77]
Sinonasal undifferentiated carcinoma	–	35–80	–	Dogan et al.,[48] Jo et al.,[49] Riobello et al.[50]
Spindle cell hemangioma	28	7.1	3.6	Kurek et al.[78]
Thyroid cancer	–	–	8–16	Murugan et al.,[79] Hemerly et al.[80]
Wilms tumor	–	–	10	Rakheja et al.[81]

IDH1/2 mutations were determined using DNA sequencing and antibodies. *IDH1* or *IDH2* mutations other than the missense mutation causing substitution at IDH1 R132 and IHD2 R172 or R140, known as non-canonical mutations, are also listed (other mutIDH1/2). Data table created by I.C.H. (also reproduced in Cadoux-Hudson et al.)[82]

some 2-OG-dependent dioxygenases and metabolic enzymes, leading to altered cellular metabolism presumed to support or promote tumorigenesis.[86–88] In myeloid cancers, mutations in *IDH1/2* are considered important for disease progression via similar mechanisms.[85] The presence of mutIDH1 or mutIDH2 in cell models results in alteration of covalent post-oligomerization modifications (e.g., methylation) to the nucleic acid and histone components of chromatin (“epigenetic” modifications).[89,90] Interestingly, it has been reported that maintenance of altered “epigenetic” modifications does not appear to be dependent on the continued presence of active mutIDH,[89,91] except in the case of myeloid cancers.[92–94]

Comparing *mutIDH1/2* with WT *IDH1/2* cells has revealed alterations to central carbon metabolism, amino acid metabolism, lipid metabolism, and redox homeostasis.[95–116] However, there is currently no consensus regarding the precise roles of these changes in relation to cancer development. This knowledge gap has relevance for development and efficacy of therapeutic approaches that currently focus on mutIDH enzyme inhibition. For example, treatment of AML with synthetic small-molecule mutIDH inhibitors leads to a reduction in *R*-2-HG levels, but resistance to first-generation inhibitors has also emerged.[117–120] A better understanding of how altered metabolism is linked to mechanisms of tumor development in *IDH1/2* mutant cancers will therefore support new diagnostic, prognostic, and therapeutic developments.

Research into *IDH1/2* mutations over the last decade, including developing an understanding of their effects on cell function, has been facilitated by multiple state-of-the-art analytical techniques and approaches. Targeted and discovery-driven metabolomics, using nuclear magnetic resonance (NMR) and mass spectrometry (MS), have been techniques at the forefront of investigating altered metabolism in cells, tissues, and biofluids.[121] Magnetic resonance spectroscopy (MRS) is one of few methods capable of measuring metabolite levels *in vivo* non-invasively and has been applied to analysis of *R*-2-HG levels in individuals with *IDH1/2* mutant glioma.[122–124] However, it remains unclear which *R*-2-HG-linked metabolic changes, beyond the increase of *R*-2-HG itself, are important in tumor development and which are bystanders in the processes of cellular transformation and tumorigenesis.

We review metabolic changes reported in the most common mutant *IDH1/2* cancers in models that include cells lines, animal models with patient-derived xenografts (PDXs) and patient tissue biopsy (PTB) samples. We evaluate reports of changes in metabolite levels and altered metabolic pathways linked to *IDH1* and *IDH2* mutations that used a range of analytical platforms, including MS, NMR, and MRS. We also discuss the potential for specific changes in metabolic pathways to act as new therapeutic targets.

WT FUNCTIONS OF IDH1, IDH2, AND IDH3

There are three isoforms of human IDH, the closely related homodimeric IDH1 and IDH2 and the heterotetrameric IDH3, all of which catalyze conversion of isocitrate to 2-OG and CO_2. IDH3 simultaneously reduces nicotinamide adenine dinucleotide (NAD^+) to produce NADH, whereas IDH1 and IDH2 reduce NAD phosphate ($NADP^+$) to NADPH.[125] IDH1 and IDH2 can catalyze the reverse reaction (i.e., reductive carboxylation of 2-OG with CO_2[126,127]), but IDH3 has been reported not to do this under physiological conditions.[128]

The human IDH isoforms have distinctive roles in ‘normal’ cellular metabolism (Figure 1). IDH1 localizes to the cytosol and peroxisomes, whereas IDH2 and IDH3 localize to the mitochondrial matrix.[129–132] IDH1 normally provides the cytosol and peroxisomes with NADPH, which is used in fatty acid synthesis or to protect from oxidative damage.[133–135] In cells with damaged mitochondria or those in hypoxia, for example, IDH1 can indirectly provide acetyl-coenzyme A (CoA) for fatty acid synthesis by catalyzing the reductive carboxylation of glutamine-derived 2-OG to isocitrate; isocitrate is isomerized to citrate, and then ATP citrate lyase cleaves it to acetyl-CoA and

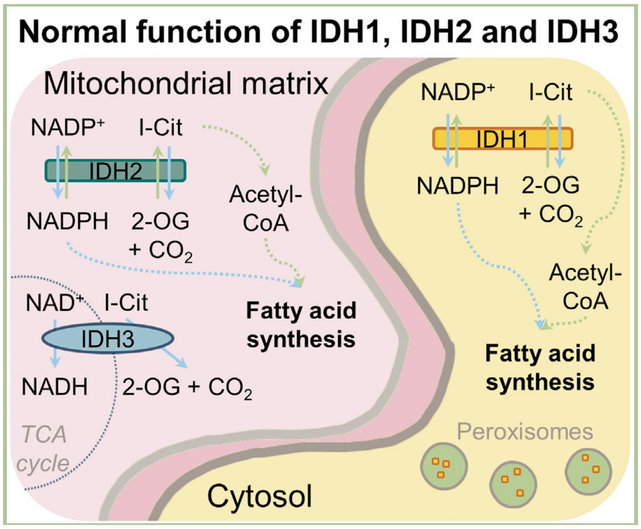

Figure 1. WT functions of IDH1, IDH2, and IDH3

IDH1 localizes in the cytosol and peroxisomes; IDH2 and IDH3 localize in mitochondria. IDH1 and IDH2 oxidize isocitrate (I-Cit) to 2-oxoglutarate (2-OG), producing NADPH; they also reductively carboxylate 2-OG to give I-Cit under hypoxic conditions or in cells with damaged mitochondria. IDH3 is part of the TCA cycle and oxidizes I-Cit to 2-OG, producing NADH. Solid lines denote direct reactions, and dashed lines denote metabolic pathways.

oxaloacetate.[136,137] IDH2 functions similarly to IDH1 but in the context of the mitochondrial matrix, providing NADPH, helping to protect mitochondria against oxidative damage.[138,139] IDH2 also synthesizes isocitrate under hypoxia by reductive carboxylation of glutamine-derived 2-OG.[140] IDH3 takes part in mitochondrial respiration by catalyzing oxidation of isocitrate in the tricarboxylic acid (TCA) cycle, producing NADH for ATP production.[128,131]

BIOSYNTHESIS OF 2-HYDROXYGLUTARATE IN NON-MUTANT IDH CELLS

The role of 2-HG in healthy metabolism is not well understood, but the *R*- and *S*-2-HG enantiomers (Figure 2) occur in low micromolar concentrations in plasma[11,141] and urine (low millimoles per mole creatinine[10] for adults and low micromoles per millimole of creatinine in neonates[9]). 2-HG can be formed by multiple processes in cells. For example, the *R*-2-HG enantiomer results from metabolism of 5-hydroxy-*L*-lysine[142] and by a coupled reaction involving oxidation of a hydroxyacid and reduction of an oxoacid by hydroxyacid oxoacid *trans*-hydrogenase (HOT) (e.g., coupling of γ-conversion of hydroxybutyrate [GHB] to succinic semialdehyde and 2-OG to *R*-2-HG).[143,144] *R*-2-HG and *S*-2-HG can also be formed by "promiscuous" reactions catalyzed by phosphoglycerate dehydrogenase (PHGDH) and mitochondrial malate dehydrogenase (MDH2), respectively.[145,146] In hypoxia, production of *S*-2-HG increases, at least in part catalyzed by promiscuous reactions of lactate dehydrogenase A (LDHA), MDH2, and cytosolic MDH (MDH1) (Figure 2).[147] It has been proposed that *S*-2-HG supports regulation of cellular redox homeostasis under conditions of cell stress; e.g., hypoxia.[148] The increased *S*-2-HG seen in hypoxia is likely due to the increased efficiency in the promiscuous reactions by LDH and MDH under acidic conditions (pH 6.0–6.8).[149,150] Similarly, PHGDH leads to increased production of *R*-2-HG under acidic conditions.[149]

Levels of both 2-HG enantiomers are normally regulated by 2-HG dehydrogenases (2-HGDH), which convert 2-HG to 2-OG. Inborn errors of metabolism, arising from mutations to the genes for *R*- and *S*-2-HGDH, are known as *D*- or *L*-2-HG aciduria (D-2-HGA or L-2-HGA). D-2-HGA can also be caused by mutation of *IDH2*[151]. Loss of *R*-2-HGDH or *S*-2-HGDH catalysis causes accumulation of *R*- or *S*-2-HG to high levels in urine, plasma, and cerebral spinal fluid[151–156]. L-2-HGA and D-2-HGA are associated with neurological abnormalities, including developmental delay, epilepsy, and cerebral ataxia, as well as cardiomyopathy in individuals with D-2-HGA.[152–157] Interestingly, there appears to be a lack of association between D-2-HGA and cancer types commonly reported to have mutations in *IDH1* and IDH2.[158] There is also a small number of reported cases of CNS tumors developing in individuals with L-2-HGA,[159,160] but it is not always clinically observed.[161]

R-2-HG BIOSYNTHESIS IS LINKED TO IDH1 AND IDH2 MUTATIONS

IDH1 and *IDH2* point mutations in cancer are heterozygous and occur most frequently at, or closely linked to, their active sites. In IDH1, R132 is the most commonly substituted residue; in IDH2, the analogous residue R172 and R140 are the most commonly altered. For all three of these mutation sites, the specific substituted residue is often linked to a particular cancer type. Histidine is the most common residue substitution for R132 in mutIDH1 in glioma,[5,13–17] whereas cysteine is more common for chondrosarcoma[19,20,24–26] and ICC,[36,37] and in AML, histidine and cysteine occur at a similar frequency.[27–31,58] Residue R140 in mutIDH2 is most commonly substituted with glutamine in AML.[28,29] Substitution of R172 in mutIDH2 is usually by serine in chondrosarcoma,[19,20,24–26] lysine or tryptophan in ICC,[36,37] and lysine in glioma.[5,15]

Initially it was thought that mutIDH1 did not convert isocitrate to 2-OG[5] and that WT IDH1 was dominantly inhibited as a heterodimer with mutIDH1.[6] Subsequently it was discovered that common mutations (i.e., mutIDH1^{R132}, mutIDH2^{R172}, and mutIDH2^{R140}) produce *R*-2-HG, which accumulates to high levels (Figure 3).[7,8] Kinetic and structural analyses of the mutIDH1s have revealed that substitution of an active-site arginine (R132 IDH1) correlates with a lowered affinity for isocitrate and the NADPH-dependent ability to reduce 2-OG to *R*-2-HG.[6–8,162] However, it has been shown that, when observed with NMR-based enzyme assays[163] rather than a fluorescence-based assay,[164] mutIDH1^{R132H} is capable of producing *R*-2-HG from isocitrate.[163] At least in studied cases, mutIDH2 does not appear to bind to or dominantly inhibit WT IDH2[165] and does not require WT IDH2 or IDH3 to produce *R*-2-HG.[166] Cytosolic mutIDH1, however, has been reported to rely on co-expression with WT IDH1 to elevate intracellular 2-HG,[166–168] but that substrate (2-OG and NADPH) is likely not channeled from WT IDH1 to mutIDH1 in a heterodimer.[168] WT IDH1 and WT IDH2 can produce small amounts of *R*-2-HG from 2OG,[8,162] but the reaction is

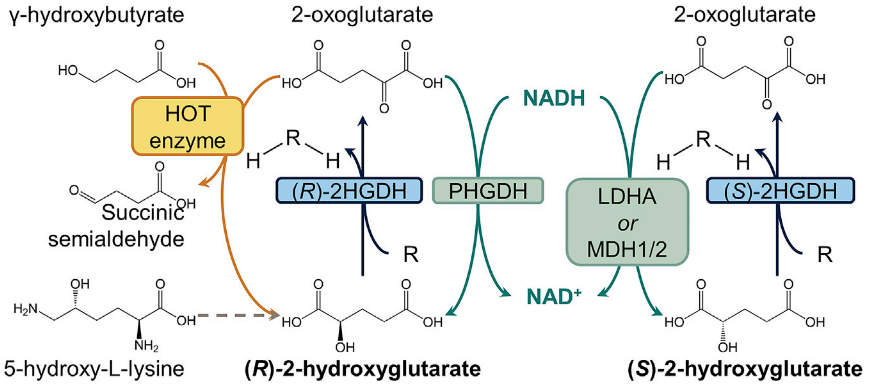

Figure 2. Biosynthesis of 2-hydroxyglutarate in non mutant IDH cells

(R)-2-HG is synthesized by metabolism of 5-hydroxy-*L*-lysine (gray dashed arrow), by hydroxyacid oxoacid *trans*-hydrogenase (HOT) catalysis (yellow), and by promiscuous catalysis by phosphoglycerate dehydrogenase (PHGDH) (green). (S)-2-HG is synthesized by promiscuous reactions involving mitochondrial malate dehydrogenase 1 and 2 (MDH1/2) and lactate dehydrogenase A (LDHA). Promiscuous reactions are in green. (*R*)-2-HG and (*S*)-2-HG are oxidized to 2-OG by (*R*)-2-HG or (*S*)-2-HG dehydrogenases ((*R*)- or (*S*)-2-HGDH), respectively, in reactions where an acceptor (R) is reduced (RH2) (blue).

limited because isocitrate binding is more efficient than that of 2-OG.[162] The ability of WT IDH1 to produce *R*-2-HG is not strongly pH dependent, unlike some other metabolic enzymes with similar promiscuous reactions.[149]

The extent of *R*-2-HG accumulation may in part depend on the residue and position with which the active site arginine is replaced. Studies of rare IDH1 substitutions (e.g., R132L/S/G) report significantly higher *R*-2-HG levels in glioma tumor tissue compared with IDH1^{R132H} and IDH1^{R132C}.[169,170] In cell models with mutIDH2^{R172}, *R*-2-HG levels were significantly higher than in models with mutIDH2^{R140Q} or mutIDH^{R132H}.[166,171] However, in HEK293T cells where mutIDH1^{R132H} was co-overexpressed with WT IDH1, the intracellular *R*-2-HG levels were similar to those of HEK293T cells expressing mutIDH2^{R172K}.[166] Furthermore, when mutIDH1^{R132H} was expressed in the mitochondria of HEK293T cells rather than in the cytosol, *R*-2-HG levels were again comparable with HEK293T cells expressing mutIDH2^{R72K}.[166]

IDH MUTANT AND WT CANCER MODELS

Developing robust pathophysiological models to study metabolism in mutant *IDH1/2* glioma has been challenging. Early attempts to establish a stable mutIDH1 glioma cell line derived from affected individuals proved difficult,[172] and it was reported that the mutant allele was lost after a small number of passages (<10).[172,173] It has been suggested that cells with prior loss of the mutIDH1 allele have a selective growth advantage in tissue culture.[91] However, loss of the mutant allele[91] or the WT allele can occur during *in vitro* culturing.[91,174–176] Most studies reporting insights into altered metabolism using cell models usegenetically engineered cell lines where the mutant enzyme is overexpressed, such as in immortalized GBM cell lines (e.g., U87, U251, or LN229), human oligodendroglioma (HOG) cells, or immortalized normal human astrocytes (NHA).[101,102,106,177–179] These cell lines provide relatively stable models to study the effects of the presence of the mutIDH1/2 enzymes, but it is possible that the process of producing the model itself may have unknown metabolic consequences and that these models do not account for some genetic and, subsequently, metabolic differences between WT IDH and mutIDH1/2 gliomas.[180–182] A limited number of glioma cell lines that endogenously express *mutIDH* have been successfully cultured from grade II astrocytomas,[183] grade III gliomas, and what were formerly known as secondary GBMs.[174,183–186] PDX mouse models bearing cells with *IDH1/2* mutations derived from affected individuals are potentially more physiologically relevant than cell culture using immortalized cell lines.[181,187,188] Several PDX-specific mutIDH1

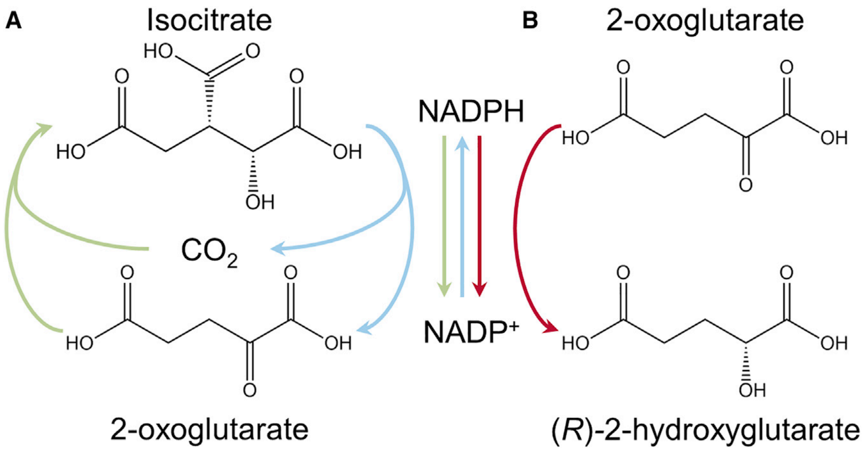

Figure 3. Normal and gain-of-function IDH1 and IDH2 reactions

(A) Normal function of IDH1 and IDH2. I-Cit is oxidized to 2-OG, and $NADP^+$ is reduced to NADPH. The reverse reaction occurs under hypoxic cell conditions.

(B) Mutant IDH1 and IDH2 reduce 2-OG to 2-HG by oxidizing NADPH to $NADP^+$.

glioma cell lines have been established,[99,188,189] but in comparison with cultured cells, these can be less practical and straightforward to work with.[181]

In contrast with glioma cells bearing *IDH* mutations, there are several cell lines derived from chondrosarcomas that harbor endogenous mutIDH1 or mutIDH2 with little to no stability issues; e.g., HT1080 and L835 (IDH1^{R132C}), JJ012 (IDH1^{R132G}), CS1 (IDH2^{R172S}), and SW1353 (IDH2^{R172K}).[95,166,190–195] JJ012 and CS1 have been successfully propagated in mice.[194] For AML, it has been common to use human primary AML cells, either as grafts in mice[196] or cultured cells.[197,198] Transfected commercially available mutIDH1 cell lines have also been established (HL60 with mutIDH1^{R132H}).[109] There are at least two ICC cell lines with endogenous *IDH1* mutations, RBE (IDH1^{R132S}) and SNU-1079 (IDH1^{R132C}), that have genetic characteristics comparable with biopsies from individuals with ICC.[42] Inducing *IDH1* or *IDH2* mutations has also been achieved in intrahepatic biliary organoids[199] as well as hepatoblasts and adult mouse liver[200] to study how the mutations promote tumorigenesis. However, despite the wide variety of non-glioma cell lines with endogenous *IDH1/2* mutations, there are very few comprehensive studies addressing metabolic changes in these models. This review reports predominantly on glioma models because it reflects their extensive use in the literature on *IDH1/2* mutations to date.

METABOLIC CHANGES IN MUTANT IDH CANCERS

Altered metabolite levels in mutant IDH cancer cell and tumor models

Although there is a lack of comprehensive studies on broader metabolism in mutant *IDH1/2* cancers, there have been numerous reports of elevated *R*-2-HG levels. Comparison of WT IDH1/2 and mutIDH1/2 cells has revealed a more than 100-fold change (FC) in *R*-2-HG levels for chondrosarcoma cells (HT1080),[95] glioma cells (LN18),[113] glioma PDX mouse models,[188] and glioma PTBs.[7] A more than 50-FC increase in *R*-2-HG levels in mutIDH1/2 cells derived from individuals with AML compared with WT IDH1/2 cells has also been reported.[58] Multiple studies report significant differences, but no specific FC, in *R*-2-HG levels between WT IDH1/2 and mutIDH1/2 glioma cells (U251, NHA, U87, and HOG),[101,102,107,114,201] chondrosarcoma cells (L835, JJ012, SW1353, and L2975),[202,203] glioma PDX mouse models,[99] and glioma, chondrosarcoma, and AML PTBs[8,106,116,124,204,205] and in plasma from individuals with ICC.[206]

Studies investigating altered metabolite levels in mutIDH1/2 compared with WT IDH glioma cell lines, PDX mouse models, and PTBs, using a range of analytical approaches (gas chromatography [GC]-MS, liquid chromatography [LC]-MS, capillary electrophoresis [CE]-MS, MS imaging [MSI], NMR, and MRS), have reported significantly altered metabolite levels.[99,101,102,104,106,107,113,114,116,124] Comparison of metabolite levels is usually made between WT IDH1/2 and mutIDH1/2 samples; often the difference is reported as a relative difference or FC rather than absolute concentrations. In contrast with *R*-2-HG, the abundance changes associated with other metabolites appear to be more context dependent.[99,101,102,106,107,113,114,116]

There are conflicting reports of altered lactate levels in *IDH1/2* variant-bearing cells compared with WT cells. For example, studies with mutIDH1^{R132H} and WT IDH1 HOG cell lines, PDX mouse models, and PTBs, using GC-MS, CE-MS, LC-MS, or MSI, report no change in lactate levels (Table 2).[99,106,107,116] However, three other studies report lower lactate levels in mutIDH1^{R132H} U87, NHA, and LN18 cells and PDX mouse models compared with WT cells.[102,104,113] Lactate levels in mutIDH1^{R132H} U87 GBM cells have been reported to be significantly increased.[114] An MRS study of individuals with mutIDH1^{R132H} and mutIDH2^{R172K} (grade II and III glioma) reported increased lactate compared with WT IDH1/2 gliomas.[124] In mutIDH2^{R172K} HOG and U87 cells, lactate levels have been reported as being unchanged[107] or decreased,[114] respectively. A potential confounding issue with regard to reporting lactate levels and other metabolite levels, including *R*-2-HG, is whether extracellular and intracellular pools of metabolites have been combined (e.g., when tissue samples are homogenized) or not (e.g., when 2D tissue culture cells are harvested and metabolites are extracted). For example, in studies using cultured cells,[102,107,113,114] extracellular lactate was largely removed prior to intracellular metabolite extraction and analysis, whereas studies using PTBs or PDX mouse models used extracts from whole tissue[99,106,116] or other methods unlikely to distinguish intracellular and extracellular lactate levels; i.e., MSI[99] or *in vivo* MRS.[104,124]

Pyruvate, as measured by LC-MS and MSI in mutIDH1^{R132H} glioma tissue and PDX mouse models as well as in mutIDH1^{R132H}-expressing LN18 or HOG cell lines, showed no significant differences in abundance when comparing *IDH1* WT and mutant samples.[99,106,107,113] No significant changes in pyruvate levels were observed between mutIDH2^{R172K} and WT IDH2-expressing HOG cells.[107] Two studies reported pyruvate to be significantly decreased in abundance in mutIDH1^{R132H} PTBs compared with WT IDH1 PTBs.[99,116]

The TCA cycle intermediates 2-OG, citrate, *cis*-aconitate, isocitrate, and fumarate are reported to be decreased or unchanged in all model types comparing mutIDH1^{R132H} with corresponding WT IDH1 samples (Table 3).[99,101,106,107,113,114,116] Succinate, oxaloacetate, and malate are the only TCA cycle intermediates with reports of increased levels in mutIDH1^{R132H} compared with WT IDH1 in cultured cells.[101,107,114] Other studies of succinate, oxaloacetate, and malate, using PTBs, PDXs, or cultured cells, report decreased relative levels[99,107,113] or no significant change in abundance.[99,106,114,116] Two independent studies reporting relative levels of TCA cycle intermediates (using different cell lines and different analytical methods: LC-MS and NMR, respectively) for mutIDH2^{R172K} cells (HOG and U87) report decreased succinate levels.[107,114]

Changes in amino acid abundance have often been reported for mutIDH cell models, but as with the aforementioned metabolites, other than *R*-2-HG, the abundance changes are generally not consistent across studies or model types (Table 4),[102,104,106,107,114,116] with comprehensive analyses only being reported in a small number of studies.[106,107,114] Only cysteine and proline, of the 20 amino acids measured, have been reported to have the same relative abundance between WT IDH1 and mutIDH1^{R132H} in two studies reporting

CellPress
OPEN ACCESS

Table 2. Analysis of glycolysis intermediates and related metabolites in mutIDH glioma samples

Change	Mutation	Model type	Analysis method	Reference
Glucose-1-phosphate				
–	IDH1^{R132H}	PTB	CE-MS	Ohka et al.[106]
–	IDH1^{R132H}	CL (LN18)	IC-MS	Walsby-Tickle et al.[113]
Glucose-6-phosphate				
–	IDH1^{R132H}	PTB	CE-MS	Ohka et al.[106]
–	IDH1^{R132H}	PTB	GC-MS/LC-MS	Zhou et al.[116]
↓	IDH1^{R132H}	CL (U87)	NMR	Wen et al.[114]
–	IDH2^{R172K}	CL (U87)	NMR	Wen et al.[114]
6-phospho-gluconate				
–	IDH1^{R132H}	PTB	CE-MS	Ohka et al.[106]
↓	IDH1^{R132H}	PTB	GC-MS/LC-MS	Zhou et al.[116]
Ribulose-5-phosphate				
–	IDH1^{R132H}	PTB	CE-MS	Ohka et al.[106]
↓	IDH1^{R132H}	CL (LN18)	IC-MS	Walsby-Tickle et al.[113]
↓	IDH1^{R132H}	CL (U87)	NMR	Wen et al.[114]
↑	IDH2^{R172K}	CL (U87)	NMR	Wen et al.[114]
Ribose-5-phosphate				
–	IDH1^{R132H}	PTB	CE-MS	Ohka et al.[106]
–	IDH1^{R132H}	PTB	GC-MS/LC-MS	Zhou et al.[116]
↓	IDH1^{R132H}	CL (LN18)	IC-MS	Walsby-Tickle et al.[113]
Seduheptulose-7-phosphate				
–	IDH1^{R132}	PTB	CE-MS	Ohka et al.[106]
–	IDH1^{R132H}	PDX	MSI/LC-MS	Fack et al.[99]
↓	IDH1^{R132H}	CL (LN18)	IC-MS	Walsby-Tickle et al.[113]
Fructose-1,6-bisphosphate				
↓	IDH1^{R132H}	PTB	GC-MS/LC-MS	Zhou et al.[116]
–	IDH1^{R132H}	CL (LN18)	IC-MS	Walsby-Tickle et al.[113]
Fructose-6-phosphate				
–	IDH1^{R132}	PTB	CE-MS	Ohka et al.[106]
–	IDH1^{R132H}	PTB	GC-MS/LC-MS	Zhou et al.[116]
–	IDH1^{R132H}	CL (LN18)	IC-MS	Walsby-Tickle et al.[113]
–	IDH1^{R132H}	CL (U87)	NMR	Wen et al.[114]
–	IDH2^{R172K}	CL (U87)	NMR	Wen et al.[114]
Dihydroxyacetone phosphate				
–	IDH1^{R132H}	PTB	CE-MS	Ohka et al.[106]
Glyceraldehyde-3-phosphate				
–	IDH1^{R132H}	PTB	CE-MS	Ohka et al.[106]
↑	IDH1^{R132H}	CL (LN18)	IC-MS	Walsby-Tickle et al.[113]
Phosphoenolpyruvate				
–	IDH1^{R132H}	PTB	CE-MS	Ohka et al.[106]
↓	IDH1^{R132H}	PTB	GC-MS/LC-MS	Zhou et al.[116]
↑	IDH1^{R132H}	CL (LN18)	IC-MS	Walsby-Tickle et al.[113]
3-phospho-glycerate				
–	IDH1^{R132H}	PTB	CE-MS	Ohka et al.[106]
↓	IDH1^{R132H}	PTB	GC-MS/LC-MS	Zhou et al.[116]
↓	IDH1^{R132H}	CL (LN18)	IC-MS	Walsby-Tickle et al.[113]
Acetyl-CoA				
↑	IDH1^{R132H}	CL (HOG)	LC-MS	Reitman et al.[107]
↑	IDH2^{R172K}	CL (HOG)	LC-MS	Reitman et al.[107]
Pyruvate				
–	IDH1^{R132H}	PTB	CE-MS	Ohka et al.[106]
–	IDH1^{R132H}	PDX	MSI/LC-MS	Fack et al.[99]
–	IDH1^{R132H}	CL (HOG)	LC-MS	Reitman et al.[107]
–	IDH1^{R132H}	CL (LN18)	IC-MS	Walsby-Tickle et al.[113]
↓	IDH1^{R132H}	PTB	GC-MS/LC-MS	Zhou et al.[116]
↓	IDH1^{R132H}	PTB	LC-MS	Fack et al.[99] (suppl.)
–	IDH2^{R172K}	CL (HOG)	LC-MS	Reitman et al.[107]
Lactate				
–	IDH1^{R132H}	PTB	CE-MS	Ohka et al.[106]
–	IDH1^{R132H}	PTB	GC-MS/LC-MS	Zhou et al.[116]
–	IDH1^{R132H}	PTB	LC-MS	Fack et al.[99] (suppl.)
–	IDH1^{R132H}	PDX	MSI/LC-MS	Fack et al.[99]
–	IDH1^{R132H}	CL (HOG)	LC-MS	Reitman et al.[107]
↓	IDH1^{R132H}	PDX	MRSI	Lenting et al.[104]
↓	IDH1^{R132H}	CL (U87)	NMR	Izquierdo-Garcia et al.[102]
↓	IDH1^{R132H}	CL (NHA)	NMR	Izquierdo-Garcia et al.[102]

(*Continued on next page*)

Table 2. *Continued*

Change	Mutation	Model type	Analysis method	Reference
↓	IDH1^{R132H}	CL (LN18)	IC-MS	Walsby-Tickle et al.[113]
↑	IDH1^{R132H}	CL (U87)	NMR	Wen et al.[114]
-	IDH2^{R172K}	CL (HOG)	LC-MS	Reitman et al.[107]
↓	IDH2^{R172K}	CL (U87)	NMR	Wen et al.[114]

Changes in metabolite levels in mutIDH glioma samples relative to WT IDH glioma samples. -, not significantly different; ↓, significantly lower in mutIDH1; ↑, significantly higher in mutIDH1; PTB, patient tissue biopsy; PDX, patient-derived mouse xenograft; CL, cell line; suppl., from supplemental information.

them.[106,107] Despite a lack of agreement in abundance changes across models and techniques, the consistent modulation of amino acids in the context of *IDH1* mutations generally is interesting and merits further study.

Comparisons of mutIDH1$^{R132H/C}$, mutIDH2$^{R172K/W/G}$, and WT IDH1 glioma, using *in vivo* MRS in humans, has shown that *N*-acetylated amino acids (NAAAs) are consistently decreased in all tumor types measured compared with healthy tissue.[122–124] Orthotopic mutIDH1^{R132H} and WT IDH1 glioma PDX mouse models similarly show lower levels of NAAAs compared with healthy tissue.[99,104] In one study comparing the abundance of NAAAs in individuals with mutIDH1^{R132H} and WT IDH1 glioma, MRS revealed that total NAAAs were slightly higher in mutIDH1^{R132H} than WT IDH1 gliomas.[124] On the other hand, it was found that specific NAAAs were depleted in mutIDH1^{R132H} cells compared with WT IDH1 cells (Table 5).[107,113] These differences may be linked to concomitant differences in amino acid abundance *in vivo* and *in vitro*, but this link requires further confirmation.

Glutathione, in its thiol or disulfide forms, has been reported as lower in mutIDH1/2 compared with WT IDH1/2 cultured cells in four studies,[102,107,113,114] except for mutIDH1^{R132H} U87 cells (increased)[114] and mutIDH1^{R132H} NHA cells (unchanged)[102] (Table 6). Interestingly, a different study also using mutIDH1^{R132H} U87 cells, reported lower glutathione disulfide levels compared with WT IDH1 U87 cells.[102] Both U87 studies used NMR measurements, and both expressed mutIDH1 and WT IDH1 using a lentiviral vector; it is unclear why different relative glutathione levels were observed.[102,114] The one study reporting on glutathione levels in tissues did not find a significant difference between mutIDH1^{R132H} and WT IDH1 PDX samples or PTBs.[99] Few studies have reported levels of other redox metabolites directly (e.g., NADP/NADPH or NAD/NADH), or energy "currency" compounds (e.g., creatine, AMP/ADP/ATP).[99,102,113,114]

Studies of altered metabolite abundance in the presence of mutIDH (all reported as significant) are inconsistent across model types (e.g., cultured cells versus PTB/PDX) and/or analysis methods (e.g., MS, NMR, and MRS) (Tables 2, 3, 4, 5, and 6). The differences in reported relative levels of metabolites likely results from multiple factors, including the varied genetic backgrounds of the multiple cell models used. The cell lines discussed are especially relevant in this respect because they represent a mixture of cancerous and non-cancerous cell types (e.g., HOG and NHA) or gliomas with different mutational landscapes (e.g., U87, U251 and LN18). In addition, "background" mutations also have the potential to contribute to metabolic differences observed between cell types for mutIDH1 and *R*-2-HG effects, previously highlighted by, e.g., Carbonneau et al.[182]

Furthermore, it is unclear to what extent the altered metabolite levels directly result from raised *R*-2-HG levels (for example, directly affected by *R*-2-HG-mediated enzyme inhibition) or result from secondary effects (for example, the consequence of altered redox equilibrium because of changes in NADPH production mediated by mutIDH). It is also possible that differences in cell proliferation rates lead to metabolic differences, as reported for a number of isogenic cell lines,[207–210] which are commonly used when studying the effects of mutIDH1 in glioma. The slower proliferation rate of mutIDH1 cells (compared with the WT) has also been reported for glioma cells derived from affected individuals[173] and human leukemic cells exposed to *R*-2-HG.[211] Currently, other than for elevated *R*-2-HG, it is difficult to form clear conclusions regarding metabolic adaptions in mutIDH1/2 glioma based on changes in metabolite levels alone. However, when combined with information from additional techniques (e.g., isotopic tracer experiments, proteomics and transcriptomics data) and information about the models, a somewhat clearer picture of metabolic changes at a functional (e.g., pathways) level in mutIDH1/2 models starts to emerge. A discussion of studies in this wider context is provided next.

Mutant IDH1 glioma cells are less glycolytic and have altered TCA cycle function compared with WT cells

Recent studies in which levels of metabolic enzymes were measured in PDX mice or PTBs found that mutIDH1^{R132H} gliomas appear to rely less on glycolysis and more on mitochondrial metabolism to alleviate mutIDH1-related metabolic stress.[104,212,213] These results support the proposal that some mutIDH1^{R132H} gliomas use lactate and glutamate as anaplerotic substrates for TCA cycle metabolism.[104,212,213] In contrast, it has been proposed that WT IDH1 gliomas are more dependent on glucose, glutamine, and acetate as anaplerotic substrates (Figure 4).[104,212,214,215] In mutIDH1 glioma, glutamate and lactate appear to be further metabolized by deamination of glutamate to 2-OG and carboxylation of pyruvate (from imported lactate) to give oxaloacetate, respectively.[104,212,213]

MutIDH1^{R132H} gliomas are reported to have reduced glucose uptake compared with WT IDH1 gliomas.[104,173,212] Cultured mutIDH1^{R132H} NHA and glioma (BT142) cells have reduced expression of the mono-carboxylate exporters MCT-1 and MCT-4 compared with WT IDH glioma cells (NHA and U87),[216,217] supporting the hypothesis that mutIDH1 gliomas are less glycolytic than WT IDH1 gliomas. LDHA, which catalyzes oxidation of pyruvate to lactate, is downregulated in mutIDH1^{R132H} glioma cells, PDX (mouse), and PTBs,[213,217,218] whereas LDHB (which converts lactate to pyruvate) has increased expression in mutIDH1^{R132H}-expressing BT142 cells, PTBs, and PDX (mouse) gliomas.[104,212,213,217] Isotope tracer experiments show that production of intracellular lactate from hyperpolarized [1-^{13}C]-pyruvate is significantly lower in mutIDH1^{R132H} versus WT IDH1 NHA cells.[216] A similar experiment comparing BT142 (mutIDH1^{R132H}) with U87 (WT IDH1) cells in cell culture and mouse tumor models

Table 3. Analysis of TCA cycle intermediates in mutIDH glioma samples

Change	Mutation	Model type	Analysis method	Reference
2-OG				
–	IDH1^{R132H}	PTB	CE-MS	Ohka et al.[106]
–	IDH1^{R132H}	CL (U87)	NMR	Wen et al.[114]
–	IDH1^{R132H}	CL (U251)	LC-MS	Gelman et al.[101]
↓	IDH1^{R132H}	PTB	GC-MS/ LC-MS	Zhou et al.[116]
↓	IDH1^{R132H}	PTB	LC-MS	Fack et al.[99] (suppl.)
↓	IDH1^{R132H}	PDX	MSI/LC-MS	Fack et al.[99]
↓	IDH1^{R132H}	CL (LN18)	IC-MS	Walsby-Tickle et al.[113]
↓	IDH1^{R132H}	CL (HOG)	LC-MS	Reitman et al.[107]
–	IDH2^{R172K}	CL (HOG)	LC-MS	Reitman et al.[107]
↓	IDH2^{R172K}	CL (U87)	NMR	Wen et al.[114]
Oxaloacetate				
–	IDH1^{R132H}	PTB	GC-MS/ LC-MS	Zhou et al.[116]
↑	IDH1^{R132H}	CL (U87)	NMR	Wen et al.[114]
↓	IDH2^{R172K}	CL (U87)	NMR	Wen et al.[114]
Citrate				
–	IDH1^{R132H}	PTB	CE-MS	Ohka et al.[106]
–	IDH1^{R132H}	PTB	GC-MS/ LC-MS	Zhou et al.[116]
–	IDH1^{R132H}	PTB	LC-MS	Fack et al.[99] (suppl.)
–	IDH1^{R132H}	PDX	MSI/LC-MS	Fack et al.[99]
–	IDH1^{R132H}	CL (U87)	NMR	Wen et al.[114]
–	IDH1^{R132H}	CL (LN18)	IC-MS	Walsby-Tickle et al.[113]
–	IDH1^{R132H}	CL (U251)	LC-MS	Gelman et al.[101]
↓	IDH1^{R132H}	CL (HOG)	LC-MS	Reitman et al.[107]
↑	IDH2^{R172K}	CL (HOG)	LC-MS	Reitman et al.[107]
↓	IDH2^{R172K}	CL (U87)	NMR	Wen et al.[114]
Cis-aconitate				
–	IDH1^{R132H}	PTB	CE-MS	Ohka et al.[106]
↓	IDH1^{R132H}	CL (HOG)	LC-MS	Reitman et al.[107]
Isocitrate				
–	IDH1^{R132H}	PTB	CE-MS	Ohka et al.[106]
↓	IDH1^{R132H}	PTB	GC-MS/ LC-MS	Zhou et al.[116]
Succinate				
–	IDH1^{R132H}	PTB	CE-MS	Ohka et al.[106]
–	IDH1^{R132H}	PTB	LC-MS	Fack et al.[99] (suppl.)
–	IDH1^{R132H}	PDX	MSI/LC-MS	Fack et al.[99]
↓	IDH1^{R132H}	CL (LN18)	IC-MS	Walsby-Tickle et al.[113]
↑	IDH1^{R132H}	CL (HOG)	LC-MS	Reitman et al.[107]
↑	IDH1^{R132H}	CL (U251)	LC-MS	Gelman et al.[101]
↑	IDH1^{R132H}	CL (U87)	NMR	Wen et al.[114]
↓	IDH2^{R172K}	CL (U87)	NMR	Wen et al.[114]
↓	IDH2^{R172K}	CL (HOG)	LC-MS	Reitman et al.[107]
Fumarate				
–	IDH1^{R132H}	PTB	CE-MS	Ohka et al.[106]
–	IDH1^{R132H}	PTB	GC-MS/ LC-MS	Zhou et al.[116]
–	IDH1^{R132H}	CL (U87)	NMR	Wen et al.[114]
–	IDH1^{R132H}	CL (U251)	LC-MS	Gelman et al.[101]
↓	IDH1^{R132H}	CL (HOG)	LC-MS	Reitman et al.[107]
↓	IDH1^{R132H}	CL (LN18)	IC-MS	Walsby-Tickle et al.[113]
–	IDH2^{R172K}	CL (U87)	NMR	Wen et al.[114]
↓	IDH2^{R172K}	CL (HOG)	LC-MS	Reitman et al.[107]
Malate				
–	IDH1^{R132H}	PTB	LC-MS	Fack et al.[99] (suppl.)
–	IDH1^{R132H}	CL (U87)	NMR	Wen et al.[114]
↓	IDH1^{R132H}	PDX	MSI/LC-MS	Fack et al.[99]
↓	IDH1^{R132H}	CL (LN18)	IC-MS	Walsby-Tickle et al.[113]
↓	IDH1^{R132H}	CL (HOG)	LC-MS	Reitman et al.[107]
↑	IDH1^{R132H}	CL (U251)	LC-MS	Gelman et al.[101]
–	IDH2^{R172K}	CL (U87)	NMR	Wen et al.[114]
↓	IDH2^{R172K}	CL (HOG)	LC-MS	Reitman et al.[107]

Changes in metabolite levels in mutIDH glioma samples relative to WT IDH glioma samples. –, not significantly different; ↓, significantly lower in mutIDH1; ↑, significantly higher in mutIDH1; PTB, patient tissue biopsy; PDX, patient-derived mouse xenograft; CL, cell line; suppl., from supplemental information.

showed that there is significantly less labeled lactate in mutIDH1 compared with WT IDH1 cells after perfusion with hyperpolarized [1-^{13}C]-pyruvate.[217] However, levels of isotopically labeled lactate derived from [1-^{13}C]-glucose tracer experiments have been reported as being significantly lower in mtIHD1^{R132H} cells (NHA)[216] and unchanged in U87 mutIDH1^{R132H} and WT IDH1 cells.[219] It has been reported that it can take a number of cell growth cycles (passages) for sufficient promoter region hypermethylation of, e.g., the *LDHA* gene to affect expression levels;[218,220] therefore, whether lactate level changes are particularly cell line dependent or sensitive to passage number after induction of mutIDH1^{R132H} remains to be determined.

As an anaplerotic substrate for the TCA cycle, pyruvate can be converted to oxaloacetate by pyruvate carboxylase (PC) and to acetyl-CoA by pyruvate dehydrogenase (PDH). In mutIDH1^{R132H} U87 and NHA cells, PC showed increased expression levels and activity, whereas PDH had reduced activity.[177,219] Furthermore, the fractional flux of pyruvate through PC was increased in mutIDH1^{R132H} NHA cells compared with WT IDH1 cells, and the fractional flux of pyruvate through PDH was decreased.[177]

Table 4. Analysis of amino acids in mutIDH glioma samples

Change	Mutation	Model type	Analysis method	Reference
**Glutamate**				
–	$IDH1^{R132H}$	PTB	GC-MS/ LC-MS	Zhou et al.[116]
–	$IDH1^{R132H}$	PDX	MRSI	Lenting et al.[104]
↓	$IDH1^{R132H}$	PTB	CE-MS	Ohka et al.[106]
↓	$IDH1^{R132H}$	PTB	LC-MS	Fack et al.[99] (suppl.)
↓	$IDH1^{R132H}$	PTB	NMR	Jalbert et al.[103]
↓	$IDH1^{R132H}$	CL (U87)	NMR	Wen et al.[114]
↓	$IDH1^{R132H}$	CL (HOG)	LC-MS	Reitman et al.[107]
↓	$IDH1^{R132H}$	CL (U87)	NMR	Izquierdo-Garcia et al.[102]
↓	$IDH1^{R132H}$	CL (NHA)	NMR	Izquierdo-Garcia et al.[102]
↑	$IDH1^{R132H}$	CL (U251)	LC-MS	Gelman et al.[101]
–	$IDH2^{R172K}$	CL (HOG)	LC-MS	Reitman et al.[107]
↑	$IDH2^{R172K}$	CL (U87)	NMR	Wen et al.[114]
**Aspartate**				
–	$IDH1^{R132H}$	PTB	CE-MS	Ohka et al.[106]
–	$IDH1^{R132H}$	PTB	GC-MS/ LC-MS	Zhou et al.[116]
–	$IDH1^{R132H}$	CL (NHA)	NMR	Izquierdo-Garcia et al.[102]
↓	$IDH1^{R132H}$	PTB	LC-MS	Fack et al.[99] (suppl.)
↓	$IDH1^{R132H}$	CL (HOG)	LC-MS	Reitman et al.[107]
↓	$IDH1^{R132H}$	CL (U87)	NMR	Izquierdo-Garcia et al.[102]
↓	$IDH2^{R172K}$	CL (HOG)	LC-MS	Reitman et al.[107]
**Alanine**				
–	$IDH1^{R132H}$	PTB	CE-MS	Ohka et al.[106]
↓	$IDH1^{R132H}$	PTB	GC-MS/ LC-MS	Zhou et al.[116]
↓	$IDH1^{R132H}$	CL (U87)	NMR	Wen et al.[114]
↑	$IDH1^{R132H}$	CL (HOG)	LC-MS	Reitman et al.[107]
↓	$IDH2^{R172K}$	CL (U87)	NMR	Wen et al.[114]
↑	$IDH2^{R172K}$	CL (HOG)	LC-MS	Reitman et al.[107]
**Arginine**				
–	$IDH1^{R132H}$	PTB	CE-MS	Ohka et al.[106]
↑	$IDH1^{R132H}$	CL (HOG)	LC-MS	Reitman et al.[107]
↓	$IDH2^{R172K}$	CL (HOG)	LC-MS	Reitman et al.[107]
**Asparagine**				
↓	$IDH1^{R132H}$	PTB	CE-MS	Ohka et al.[106]
↑	$IDH1^{R132H}$	PTB	GC-MS/ LC-MS	Zhou et al.[116]
↑	$IDH1^{R132H}$	CL (HOG)	LC-MS	Reitman et al.[107]
↑	$IDH2^{R172K}$	CL (HOG)	LC-MS	Reitman et al.[107]

Table 4. *Continued*

Change	Mutation	Model type	Analysis method	Reference
**Cysteine**				
–	$IDH1^{R132H}$	PTB	CE-MS	Ohka et al.[106]
–	$IDH1^{R132H}$	CL (HOG)	LC-MS	Reitman et al.[107]
–	$IDH2^{R172K}$	CL (HOG)	LC-MS	Reitman et al.[107]
**Glutamine**				
–	$IDH1^{R132H}$	PTB	GC-MS/ LC-MS	Zhou et al.[116]
–	$IDH1^{R132H}$	PTB	LC-MS	Fack et al.[99] (suppl.)
–	$IDH1^{R132H}$	CL (NHA)	NMR	Izquierdo-Garcia et al.[102]
↓	$IDH1^{R132H}$	PTB	CE-MS	Ohka et al.[106]
↓	$IDH1^{R132H}$	CL (U87)	NMR	Izquierdo-Garcia et al.[102]
↑	$IDH1^{R132H}$	PDX	MRSI	Zhou et al.[116]
↑	$IDH1^{R132H}$	CL (HOG)	LC-MS	Reitman et al.[107]
↑	$IDH2^{R172K}$	CL (HOG)	LC-MS	Reitman et al.[107]
**Glycine**				
–	$IDH1^{R132H}$	PTB	CE-MS	Ohka et al.[106]
↓	$IDH1^{R132H}$	PTB	GC-MS/ LC-MS	Zhou et al.[116]
↓	$IDH1^{R132H}$	CL (U87)	NMR	Wen et al.[114]
↑	$IDH1^{R132H}$	CL (HOG)	LC-MS	Reitman et al.[107]
↓	$IDH2^{R172K}$	CL (U87)	NMR	Wen et al.[114]
↑	$IDH2^{R172K}$	CL (HOG)	LC-MS	Reitman et al.[107]
**Histidine**				
–	$IDH1^{R132H}$	PTB	CE-MS	Ohka et al.[106]
↑	$IDH1^{R132H}$	CL (HOG)	LC-MS	Reitman et al.[107]
–	$IDH2^{R172K}$	CL (HOG)	LC-MS	Reitman et al.[107]
**Isoleucine**				
–	$IDH1^{R132H}$	PTB	CE-MS	Ohka et al.[106]
↓	$IDH1^{R132H}$	CL (U87)	NMR	Wen et al.[114]
↑	$IDH1^{R132H}$	CL (HOG)	LC-MS	Reitman et al.[107]
↑	$IDH2^{R172K}$	CL (HOG)	LC-MS	Reitman et al.[107]
↑	$IDH2^{R172K}$	CL (U87)	NMR	Wen et al.[114]
**Leucine**				
–	$IDH1^{R132H}$	PTB	CE-MS	Ohka et al.[106]
↓	$IDH1^{R132H}$	CL (U87)	NMR	Wen et al.[114]
↑	$IDH1^{R132H}$	CL (HOG)	LC-MS	Reitman et al.[107]
↓	$IDH2^{R172K}$	CL (U87)	NMR	Wen et al.[114]
↑	$IDH2^{R172K}$	CL (HOG)	LC-MS	Reitman et al.[107]
**Lysine**				
–	$IDH1^{R132H}$	PTB	CE-MS	Ohka et al.[106]
↑	$IDH1^{R132H}$	CL (HOG)	LC-MS	Reitman et al.[107]
↑	$IDH2^{R172K}$	CL (HOG)	LC-MS	Reitman et al.[107]

(*Continued on next page*)

Table 4. *Continued*

Change	Mutation	Model type	Analysis method	Reference
Methionine				
-	IDH1^{R132H}	PTB	CE-MS	Ohka et al.[106]
↑	IDH1^{R132H}	CL (HOG)	LC-MS	Reitman et al.[107]
↑	IDH2^{R172K}	CL (HOG)	LC-MS	Reitman et al.[107]
Phenylalanine				
↓	IDH1^{R132H}	PTB	CE-MS	Ohka et al.[106]
↓	IDH1^{R132H}	CL (U87)	NMR	Wen et al.[114]
↑	IDH1^{R132H}	CL (HOG)	LC-MS	Reitman et al.[107]
↓	IDH2^{R172K}	CL (U87)	NMR	Wen et al.[114]
↑	IDH2^{R172K}	CL (HOG)	LC-MS	Reitman et al.[107]
Proline				
-	IDH1^{R132H}	PTB	CE-MS	Ohka et al.[106]
-	IDH1^{R132H}	CL (HOG)	LC-MS	Reitman et al.[107]
↓	IDH2^{R172K}	CL (HOG)	LC-MS	Reitman et al.[107]
Serine				
↓	IDH1^{R132H}	PTB	GC-MS/ LC-MS	Zhou et al.[116]
↑	IDH1^{R132H}	CL (HOG)	LC-MS	Reitman et al.[107]
-	IDH2^{R172K}	CL (HOG)	LC-MS	Reitman et al.[107]
Threonine				
-	IDH1^{R132H}	PTB	CE-MS	Ohka et al.[106]
↓	IDH1^{R132H}	CL (U87)	NMR	Wen et al.[114]
↑	IDH1^{R132H}	CL (HOG)	LC-MS	Reitman et al.[107]
↑	IDH2^{R172K}	CL (U87)	NMR	Wen et al.[114]
↑	IDH2^{R172K}	CL (HOG)	LC-MS	Reitman et al.[107]
Tryptophan				
-	IDH1^{R132H}	PTB	CE-MS	Ohka et al.[106]
↑	IDH1^{R132H}	CL (HOG)	LC-MS	Reitman et al.[107]
↑	IDH2^{R172K}	CL (HOG)	LC-MS	Reitman et al.[107]
Tyrosine				
-	IDH1^{R132H}	PTB	CE-MS	Ohka et al.[106]
↑	IDH1^{R132H}	CL (HOG)	LC-MS	Reitman et al.[107]
↑	IDH2^{R172K}	CL (HOG)	LC-MS	Reitman et al.[107]
Valine				
-	IDH1^{R132H}	PTB	CE-MS	Ohka et al.[106]
↓	IDH1^{R132H}	CL (U87)	NMR	Wen et al.[114]
↑	IDH1^{R132H}	CL (HOG)	LC-MS	Reitman et al.[107]
↑	IDH1^{R132H}	CL (U87)	NMR	Izquierdo-Garcia et al.[102]
↑	IDH1^{R132H}	CL (NHA)	NMR	Izquierdo-Garcia et al.[102]
↑	IDH2^{R172K}	CL (U87)	NMR	Wen et al.[114]
↑	IDH2^{R172K}	CL (HOG)	LC-MS	Reitman et al.[107]

Changes in metabolite levels in mutIDH glioma samples relative to WT IDH glioma samples. -, not significantly different; ↓, significantly lower in mutIDH1; ↑, significantly higher in mutIDH1; PTB, patient tissue biopsy; PDX, patient-derived mouse xenograft; CL, cell line; suppl., from supplemental information.

Thus, by reducing PDH activity and increasing PC levels, mutIDH1^{R132H} glioma U87 and NHA cells have been shown to use pyruvate for production of oxaloacetate, a process supported by separate studies.[177,219] In general, there appears to be experimental agreement that gliomas with mutIDH1R^{R132H} are less glycolytic and rely more on oxidative phosphorylation than WT IDH1 gliomas.[99,101,104,188,212,217,218]

Glutamate is an important anaplerotic substrate in mutant IDH1 glioma cells

Glutamate dehydrogenase 1 (GLUD1) and GLUD2, which catalyze oxidative deamination of glutamate to 2-OG, are significantly elevated in mutIDH1^{R132H} glioma compared with WT IDH1 glioma,[104,212,213,221,222] indicating the potential for increased glutamate utilization by the TCA cycle. Moreover, increased expression of nerve-tissue-specific GLUD2 leads to enhanced tumor growth in mutIDH1^{R132H} glioma murine models.[221,222]

Branched-chain amino acid transaminase 1 (BCAT1), which is located in the cytosol and widely expressed in the brain,[223] is present at significantly lower levels in mutIDH1^{R132H} glioma PTB and PDX compared with WT IDH1 glioma samples.[104,212,224] BCAT1 catalyzes transamination of valine, leucine, and isoleucine; the α-amino group of the amino acids is transferred to 2-OG, producing glutamate and branched-chain α-ketoacids.[225] High expression of BCAT1 may be counterproductive to glutamate in its role as an anaplerotic substrate of the TCA cycle in *IDH* mutant tumors. The reduced level of BCAT1 in mutIDH1^{R132H} cells is in part due to extensive hypermethylation of the promoter region of the *BCAT1* gene.[212,224] However, other mutIDH1-related mechanisms may be involved in regulation of *BCAT1* expression because expression of *mutIDH1* in immortalized human astrocytes causes *BCAT1* downregulation, but not by hypermethylation of its promoter region.[224] It has been reported that *R*-2-HG can directly inhibit BCAT1 activity in mutIDH1^{R132H} HOG cells at high (millimolar) concentrations,[201] although this was not the case in mouse brain detergent extracts exposed to millimolar *R*-2-HG.[224]

Glutaminolysis, where glutamine is converted to TCA cycle intermediates, is a hallmark of metabolism in several types of cancers.[226] Cultured glioma cells (D54 and U87) expressing mutIDH1^{R132H} are sensitive to inhibition of glutaminase (GLS),[106,227] the main enzyme catalyzing conversion of glutamine to glutamate, but GLS expression is not significantly increased in samples from individuals with mutIDH1^{R132H}.[104,212] The reliance on glutaminolysis in cultured cells could be due to the high levels of cystine in standard culture media. When a variety of cancer cell lines were grown in the presence of high levels of cystine, the glutamate/cystine antiporter xCT/*SLC7A11* led to a depletion of glutamate in cells, which was ameliorated via glutaminolysis[228]. Cells grown in low cystine media were significantly less sensitive to inhibition of glutaminolysis as the xCT glutamate/cystine antiporter no longer exported glutamate from the cells.[228] The importance of glutaminolysis in mutIDH1 glioma thus requires further study.

Other pathways involved in TCA cycle anaplerosis

Additional changes related to TCA cycle-linked metabolism reported in mutIDH1 cells include the γ-aminobutyric acid (GABA) shunt, lipid oxidation-derived acetyl-CoA, and function

Table 5. Analysis of NAAAs in mutIDH glioma samples

Change	Mutation	Model type	Analysis method	Reference
Total NAAA				
↑	IDH1^{R132H} IDH2^{R172K}	PTB	MRS	Emir et al.[124]
‒	IDH1^{R132H}	PDX	MRS	Lenting et al.[104]
NAAG				
↑	IDH1^{R132H}	PTB	LC-MS	Fack et al.[99] (suppl.)
‒	IDH1^{R132H}	PDX	MSI	Fack et al.[99]
↓	IDH1^{R132H}	CL (HOG)	LC-MS	Reitman et al.[107]
↓	IDH1^{R132H}	CL (LN18)	IC-MS	Walsby-Tickle et al.[113]
↓	IDH2^{R172K}	CL (HOG)	LC-MS	Reitman et al.[107]
NAAsp				
↓	IDH1^{R132H}	PTB	CE-MS	Ohka et al.[106]
↓	IDH1^{R132H}	PTB	LC-MS	Fack et al.[99] (suppl.)
‒	IDH1^{R132H}	PDX	MSI	Fack et al.[99]
↓	IDH1^{R132H}	CL (HOG)	LC-MS	Reitman et al.[107]
↓	IDH2^{R172K}	CL (HOG)	LC-MS	Reitman et al.[107]
NAAla				
↓	IDH1^{R132H}	CL (HOG)	LC-MS	Reitman et al.[107]
↓	IDH2^{R172K}	CL (HOG)	LC-MS	Reitman et al.[107]
NAGln				
↓	IDH1^{R132H}	CL (HOG)	LC-MS	Reitman et al.[107]
‒	IDH2^{R172K}	CL (HOG)	LC-MS	Reitman et al.[107]
NAGlu				
↓	IDH1^{R132H}	PTB	CE-MS	Ohka et al.[106]
↓	IDH1^{R132H}	CL (HOG)	LC-MS	Reitman et al.[107]
↓	IDH2^{R172K}	CL (HOG)	LC-MS	Reitman et al.[107]
NAGly				
↑	IDH1^{R132H}	CL (U87)	NMR	Wen et al.[114]
↓	IDH2^{R172K}	CL (U87)	NMR	Wen et al.[114]
NAHis				
↓	IDH1^{R132H}	PTB	CE-MS	Ohka et al.[106]
NAMet				
↓	IDH1^{R132H}	CL (HOG)	LC-MS	Reitman et al.[107]
‒	IDH2^{R172K}	CL (HOG)	LC-MS	Reitman et al.[107]
NASer				
↓	IDH1^{R132H}	CL (HOG)	LC-MS	Reitman et al.[107]
↓	IDH2^{R172K}	CL (HOG)	LC-MS	Reitman et al.[107]
NAThr				
↓	IDH1^{R132H}	CL (HOG)	LC-MS	Reitman et al.[107]
↓	IDH2^{R172K}	CL (HOG)	LC-MS	Reitman et al.[107]

Changes in metabolite levels in mutIDH glioma samples relative to WT IDH glioma samples. ‒, not significantly different; ↓, significantly lower in mutIDH1; ↑, significantly higher in mutIDH1; PTB, patient tissue biopsy; PDX, patient-derived mouse xenograft; CL, cell line; suppl., from supplemental information; NAAA, N-acetylated amino acids; NAAG, N-acetylaspartylglutamate; NAAsp, N-acetylaspartate; NAAla, N-acetylalanine; NAGln, N-acetylglutamine; NAGlu, N-acetylglutamate; NAGly, N-acetylglycine; NAHis, N-acetylhistidine; NAMet, N-acetylmethionine; NASer, N-acetylserine; NAThr, N-acetylthreonine.

of the 2-OG dehydrogenase complex;[101,104,109,229] the significance of these changes for tumor development is unclear. In the GABA shunt, glutamate is decarboxylated, forming GABA (catalyzed by glutamate decarboxylase [GAD-1]), followed by deamination to give succinic semialdehyde (catalyzed by 4-aminobutyrate aminotransferase [ABAT]), and finally oxidation to succinate by succinate-semialdehyde dehydrogenase (SSADH). Levels of the enzymes involved in the GABA shunt pathway are significantly elevated in mutIDH1^{R132H} glioma tissue[101,104] but not in an orthotopic xenograft mouse model of mutIDH1^{R132H} glioma.[104] In U251 glioma cells, expression of mutIDH1 or treatment of WT IDH1 cells with exogenous *R*-2-HG leads to a reduction in the pro-proliferative effects of GABA.[101] Further studies are needed to understand the effects of *R*-2-HG on enzymes in the GABA shunt and its role in glioma metabolism.

In human leukemia (HL60) mutIDH1^{R132H} cells, acetyl-CoA derived from lipid oxidation is suggested to be an anaplerotic substrate for the TCA cycle. mutIDH1^{R132H} HL60 cells are reported to have increased levels of enzymes linked to fatty acid oxidation compared with WTIDH1 HL60 cells.[109] Furthermore, mutIDH1^{R132H} glioma tumor samples have been shown to have increased levels of citrate synthase (CS)[104,212] even though PDH activity was reduced.[177,219] It remains unclear whether mutIDH1 gliomas utilize acetyl-CoA derived from lipid oxidation for anaplerosis. Finally, activity of the 2-OG dehydrogenase complex (OGDH) has been shown to be lowered by *R*-2-HG,[229] but this has yet to be explored further in the context of mutIDH1/2 cancers.

Because of the focus of research on glioma models in relation to mutIDH1 metabolism, the metabolic significance of mutIDH2 has been less well explored. MutIDH2^{R172S} SW1353 chondrosarcoma cells under mitochondrial stress (hypoxia) show an increased ability to activate reductive glutamine metabolism compared with mutIDH1^{R132C} HT1080 fibrosarcoma cells,[207], with the latter having limited ability to generate isocitrate by reductive decarboxylation.[95,207] These observations suggest that, at least in some contexts, mutIDH2 cells may be better able to alleviate metabolic stress than mutIDH1 cells, an observation that could have implications for developing new mutIDH therapeutic strategies.

ALTERED REDOX HOMEOSTASIS LINKED TO MUTANT IDH1 CONSUMPTION OF NADPH

Cells must control reactive oxygen species (ROS) to limit damage to nucleic acids, proteins and lipids and to maintain ROS-based signaling pathways.[230] Antioxidants are central to regulating ROS; glutathione is a ubiquitous antioxidant tripeptide thiol requiring NADPH for its production.[231] Cells employ multiple pathways for NADPH production; in the cytosol, major

Table 6. Analysis of other metabolites in mutIDH glioma samples

Change	Mutation	Model type	Analysis method	Reference
Glutathione (oxidized)				
↓	IDH1^{R132H}	CL (HOG)	LC-MS	Reitman et al.[107]
↓	IDH2^{R172K}	CL (HOG)	LC-MS	Reitman et al.[107]
Glutathione (reduced)				
–	IDH1^{R132H}	PTB	LC-MS	Fack et al.[99] (suppl.)
–	IDH1^{R132H}	PDX	MSI/ LC-MS	Fack et al.[99]
–	IDH1^{R132H}	CL (NHA)	NMR	Izquierdo-Garcia et al.[102]
↓	IDH1^{R132H}	CL (HOG)	LC-MS	Reitman et al.[107]
↓	IDH1^{R132H}	CL (U87)	NMR	Izquierdo-Garcia et al.[102]
↓	IDH1^{R132H}	CL (LN18)	IC-MS	Walsby-Tickle et al.[113]
↑	IDH1^{R132H}	CL (U87)	NMR	Wen et al.[114]
↓	IDH2^{R172K}	CL (U87)	NMR	Wen et al.[114]
↓	IDH2^{R172K}	CL (HOG)	LC-MS	Reitman et al.[107]
Cystathionine				
↓	IDH1^{R132H}	PDX	MSI/ LC-MS	Fack et al.[99]
Creatine				
–	IDH1^{R132H}	CL (NHA)	NMR	Izquierdo-Garcia et al.[102]
↓	IDH1^{R132H}	CL (U87)	NMR	Wen et al.[114]
↓	IDH1^{R132H}	CL (U87)	NMR	Izquierdo-Garcia et al.[102]
–	IDH2^{R172K}	CL (U87)	NMR	Wen et al.[114]
ATP				
–	IDH1^{R132H}	PTB	LC-MS	Fack et al.[99] (suppl.)
–	IDH1^{R132H}	CL (LN18)	IC-MS	Walsby-Tickle et al.[113]
↓	IDH1^{R132H}	PDX	MSI/ LC-MS	Fack et al.[99]
ADP				
–	IDH1^{R132H}	PDX	MSI/ LC-MS	Fack et al.[99]
↑	IDH1^{R132H}	CL (LN18)	IC-MS	Walsby-Tickle et al.[113]
AMP				
–	IDH1^{R132H}	PTB	LC-MS	Fack et al.[99] (suppl.)
–	IDH1^{R132H}	PDX	MSI/ LC-MS	Fack et al.[99]
–	IDH1^{R132H}	CL (LN18)	IC-MS	Walsby-Tickle et al.[113]
NAD^+				
↓	IDH1^{R132H}	CL (U87)	NMR	Wen et al.[114]
↑	IDH2^{R172K}	CL (U87)	NMR	Wen et al.[114]
NADH				
↑	IDH1^{R132H}	CL (LN18)	IC-MS	Walsby-Tickle et al.[113]

Changes in metabolite levels in mutIDH glioma samples relative to WT IDH glioma samples. –, not significantly different; ↓, significantly lower in mutIDH1; ↑, significantly higher in mutIDH1; PTB, patient tissue biopsy; PDX, patient-derived mouse xenograft; CL, cell line; suppl., from supplemental information.

contributors to ROS regulation are IDH1, malic enzyme 1 (ME1), and glucose-6-phopshate dehydrogenase (G6PD)/6-phosphogluconate dehydrogenase (PGD) in the oxidative pentose phosphate pathway (oxPPP).[232,233] IDH1 is especially important for NADPH production in the brain.[97] IDH2 plays an important role in mitochondrial redox balance and in protection against ROS,[138,139] protecting tissues such as the lungs, kidneys, heart, and liver from mitochondrial oxidative damage.[234–237] MutIDH1 and mutIDH2 have a substantially reduced ability to produce NADPH compared with the WT and instead consume significant amounts of NADPH during *R*-2-HG production.[95–97,100] This puts pressure on maintenance of the cellular NADPH/$NADP^+$ balance and redox homeostasis, potentially making mutIDH cells more vulnerable to ROS and metabolic stress.[95,96,100,105,108,110,112]

There is evidence that mutIDH1 cells employ compensatory pathways to ameliorate the increased use of NADPH for *R*-2-HG production. The PPP has been suggested to act in this role, and there is evidence of increased flux through the PPP in mutIDH1^{R132H} HCT116 and NHA cells.[100] However, such increased flux has been shown not to fully compensate for *R*-2-HG-mediated NADPH consumption, especially when the mutIDH1^{R132H} cells are under metabolic stress.[95,100] In mutIDH1^{R132H} U87 glioma, primary GBM, and immortalized astrocytes cell lines, NADPH levels were partially restored by phosphorylating NAD^+ by NAD^+ kinase.[96] The upregulation of NAD^+ synthesis enzymes varies between immortalized astrocyte and GBM cell lines as well as PTBs, indicating the changing role of mutIDH1 throughout tumorigenesis.[96] MutIDH1^{R132H} glioma xenograft cell lines have reduced NAD^+ levels as well as lowered nicotinate phosphoribosyltransferase (Naprt1), an enzyme involved in the NAD^+ salvagepathway.[111] The mutIDH1^{R132H} glioma cells were sensitive to inhibition of nicotinamide phosphoribosyltransferase (NAMPT), the rate-limiting enzyme of the NAD^+ salvage pathway, which left the mutIDH1^{R132H} cells with few options to increase intracellular NAD^+.[111]

Glioma (BT142) cells rely on glutamate to boost redox homeostasis by increasing the NADPH/$NADP^+$ and reduced/oxidized glutathione ratios.[112] Induction of mutIDH1^{R132H} or mutIDH1^{R132C} expression in U251 glioma cell increases expression of glutathione biosynthesis enzymes.[110] The nuclear factor erythroid 2-related factor (Nrf2), which regulates the response to oxidative damage, including glutathione biosynthesis, has enhanced activity in mutIDH1$^{R132C/H}$ U251 cells.[110] MutIDH1 astrocytoma cells have displayed critical reliance on cystathionine-γ-lyase (CSE) *in vitro* and *in* vivo;[176] CSE provides cysteine for GSH synthesis via lysis of cystathionine. The reliance on CSE

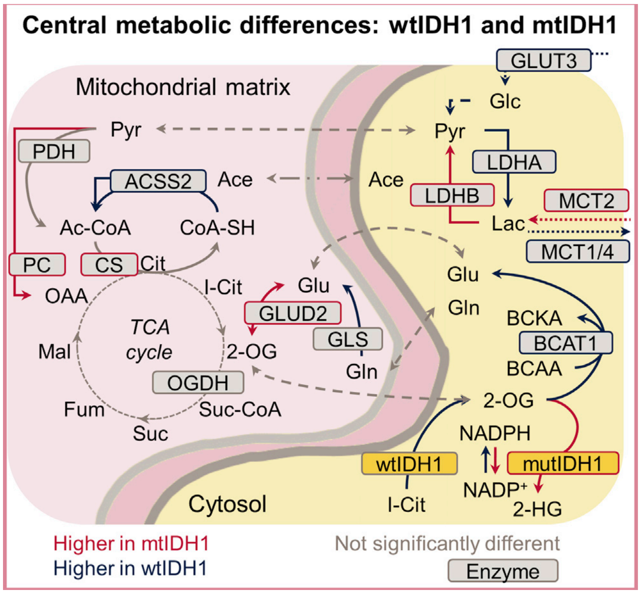

Figure 4. Mutant IDH1 glioma cells are less glycolytic and have altered TCA cycle function compared with WT cells

In mutIDH1R132H glioma cells, glutamate and lactate are favored for anaplerosis of the TCA cycle, whereas WT IDH1 gliomas are more glycolytic and use acetate and glutamine in anaplerosis of the TCA cycle. PDH, pyruvate dehydrogenase; PC, pyruvate carboxylase; CS, citrate synthase; OGDH, 2-OG complex; GLUD2, glutamate dehydrogenase; GLS, glutaminase; GLUT3, glucose transporter 3; LDHA and LDHB, lactate dehydrogenase A and B; MCT1/2/4, monocarboxylate transporter; BCAT1, branched-chain amino acid transferase; IDH, isocitrate dehydrogenase; Cit, citrate; Suc-CoA, succinyl-CoA; Suc, succinate; Fum, fumarate; Mal, malate; OAA, oxaloacetate; Pyr, Pyruvate; Ac-CoA, acetyl-CoA; Ace, acetate; Glc, glucose; Lac, lactate; Glu, glutamate; Gln, glutamine; BCAA, branched-chain amino acids; BCKA, branched-chain α-ketoacids.

was most pronounced under limited cysteine availability.[176] GBMs also have upregulated WT IDH1 expression,[238,239] and gene knockdown or pharmacological inhibition of WT IDH1 has been shown to lead to decreased NADPH and glutathione levels, along with increased ROS expression and apoptosis.[238,239] These observations suggest the importance of WT IDH1 activity in maintaining redox homeostasis.

Interestingly, mutIDH1/2 do not appear to confer survival benefits in AML,[28,240] chondrosarcoma,[24,66] or ICC[37,241] but appear to do so in glioma.[242] Additionally, in chondrosarcoma, the response to radiation treatment does not correlate with mutIDH1/2-status.[243] Thus, the current understanding of mutIDH1/2 in relation to redox homeostasis is that it is cancer-type dependent. This conclusion is of significance when developing and optimizing therapeutic approaches targeting mutIDH1/2 effects in tumor cells.

ALTERED LIPID METABOLISM IN CELLS EXPRESSING MUTANT IDH

The conversion of isocitrate to 2-OG by WT IDH1 provides NADPH that is subsequently available for fatty acid synthesis,[133] and both WT IDH1 and IDH2 support fatty acid synthesis under hypoxic conditions by providing isocitrate, which is converted to acetyl-CoA via citrate[136,140] (Figure S1). Because mutIDH1^{R132H} loses the ability to produce NADPH and to carry out reductive carboxylation,[207,244] it is reasonable to propose that cells carrying mutIDH1^{R132H} may have altered lipid metabolism compared with WT IDH1 cells.

In mutIDH1 glioma, alterations in phospholipid profiles have been observed in cultured cell models and tumors, as shown by LC-MS, MSI, *in vitro* and *ex vivo* ^{1}H and ^{31}P NMR, and *in vivo* MRS,[98,99,102,103] as summarized in part in Table 7. Independent studies using MRS/NMR show that phosphocholine (PCho) and

Table 7. Analysis of phosphorylated lipids in mutIDH glioma samples

Change	Mutation	Model type	Analysis method	Reference
Phosphocholine				
↑	IDH1^{R132H}	PTB	^{1}H NMR	Jalbert et al.[103]
–	IDH1^{R132H}	PTB	^{31}P NMR	Esmaeili et al.[98]
–	IDH1^{R132H}	PDX	^{31}P MRI	Esmaeili et al.[98]
↑	IDH1^{R132H}	CL (U251)	^{31}P NMR	Esmaeili et al.[98]
↓	IDH1^{R132H}	CL (HOG)	LC-MS	Reitman et al.[107]
↓	IDH1^{R132H}	CL (U87)	^{1}H NMR	Izquierdo-Garcia et al.[102]
↓	IDH1^{R132H}	CL (NHA)	^{1}H NMR	Izquierdo-Garcia et al.[102]
↓	IDH2^{R172K}	CL (HOG)	LC-MS	Reitman et al.[107]
Glycerophosphocholine				
↑	IDH1^{R132H}	PTB	^{1}H NMR	Jalbert et al.[103]
↑	IDH1^{R132H}	PTB	^{31}P NMR	Esmaeili et al.[98]
↑	IDH1^{R132H}	PDX	^{31}P MRI	Esmaeili et al.[98]
↑	IDH1^{R132H}	CL (U251)	^{31}P NMR	Esmaeili et al.[98]
↑	IDH1^{R132H}	CL (HOG)	LC-MS	Reitman et al.[107]
↑	IDH1^{R132H}	CL (U87)	^{1}H NMR	Izquierdo-Garcia et al.[102]
–	IDH1^{R132H}	CL (NHA)	^{1}H NMR	Izquierdo-Garcia et al.[102]
↓	IDH2^{R172K}	CL (HOG)	LC-MS	Reitman et al.[107]
Phosphoethanolamine				
–	IDH1^{R132H}	PTB	^{1}H MRI	Wenger et al.[245]
↓	IDH1^{R132H}	PTB	^{31}P NMR	Esmaeili et al.[98]
↓	IDH1^{R132H}	PDX	^{31}P MRI	Esmaeili et al.[98]
↓	IDH1^{R132H}	CL (U251)	^{31}P NMR	Esmaeili et al.[98]
Glycerophospho-ethanolamine				
–	IDH1^{R132H}	PTB	^{31}P NMR	Esmaeili et al.[98]
–	IDH1^{R132H}	PDX	^{31}P MRI	Esmaeili et al.[98]
–	IDH1^{R132H}	CL (U251)	^{31}P NMR	Esmaeili et al.[98]
Phosphatidylinositol				
↑	IDH1^{R132H}	PDX	MSI	Fack et al.[99]

Changes in metabolite levels in mutIDH glioma samples relative to WT IDH glioma samples. –, not significantly different; ↓, significantly lower in mutIDH1; ↑, significantly higher in mutIDH1; PTB, patient tissue biopsy; PDX, patient-derived mouse xenograft; CL, cell line.

glycerophosphocholine (GPCho) are increased in cultured glioma cells expressing mutIDH1^{R132H}, xenograft models, and PTBs compared with equivalent WT IDH1 glioma samples.[98,103] However, a study measuring PCho with LC-MS in cultured HOG cells expressing mutIDH1^{R132H} or mutIDH2^{R172K} found that PCho was significantly lower compared with HOG WT IDH cells[107] and reported GPCho to be increased. In addition to PCho and GPCho, phosphoethanolamine (PE) was significantly lower in mutIDH1^{R132H} gliomas across all the sample types analyzed.[98] In an MSI study, four putatively identified PE lipids have been reported to be substantially increased in mutIDH1^{R132H} glioma mouse PDXs.[99] However, the NMR methods employed were insufficiently sensitive to differentiate between the different PEs.

In addition to PE and PCho, levels of phosphatidylinositol (PI) lipids are reported as being increased when comparing mutIDH1^{R132H} and WT IDH1 glioma PDXs in mice.[99] When gliomas were analyzed in affected individuals using *in vivo* MRS measurements, no significant differences in ratios of PE/PCho, GPCho/glycerophosphoethanolamine (GPE), or (PCho+GPCho)/(PE+GPE), were detected between individuals with mutIDH1^{R132H} and WT IDH1 glioma.[245] The apparently specific differences in lipid profiles in glioma may in part be due to cells compensating for loss of WT IDH1 activity[98] by increasing IDH2-enabled NADPH and lipid production. Cells from mouse PDXs of mutIDH1 glioma have been shown to have significantly higher mitochondrial density than corresponding WT IDH1 cells,[188] an interesting observation given that IDH2 localizes to mitochondria. Additional mitochondria would also increase the lipid membrane content in cells, which could help explain the differences seen in the phospholipid composition of mutIDH1 and WT IDH1 gliomas.[98]

Cholesterol metabolism in mutIDH1/2 glioma has received limited attention to date, but a recent study suggests that it may be of therapeutic relevance.[115] It has been found that cholesterol levels were lower in brains of mutIDH1^{R132H} knockin (KI) mice and mutIDH1^{R132H}-expressing U87 and U251 cells compared with corresponding WT IDH1 samples.[115] MutIDH glioma cells had increased expression of the *de novo* cholesterol synthesis enzymes 3-hydroxy-3-methyl-glutaryl-coenzyme A reductase (HMGCR) and sterole regulatory element-binding protein 2 (SREBP2), and inhibition of HMGCR by atorvastatin led to significant cell death in mutIDH1^{R132G}-expressing U87 and U251 cells but had little effect on the WT IDH1-expressing U87 and U251 cells.[115]

Lipid metabolism in leukemia (HL60) cells with mutIDH1^{R132H} was altered compared with WT IDH1 cells.[109] Differences included increased levels of proteins involved in lipid synthesis. ^{13}C labeling experiments revealed that HL60 mutIDH1^{R132H} cells have a higher rate of fatty acid synthesis compared with WT IDH1 cells.[109] Total PI, sphingosine, sphingoanine, sphingomyelin, free cholesterol, and monounsaturated fatty acid (MUFA) levels were significantly higher, but esterified cholesterol was significantly lower, in mutIDH1^{R132H} compared with WT IDH1 HL60 cells. Fatty acid synthesis in HL60 cells under normoxic conditions relied on glucose, not glutamine, as the main carbon source.[109] In a chondrosarcoma (HT1080) cell study, no increases in expression of fatty acid synthesis-related genes were observed in mutIDH1^{R132C} relative to WT IDH1 cells. However, *R*-2-HG production has been shown to limit the metabolic flexibility of cells under stress (de-lipidated media or hypoxia) because of shunting of NADPH toward 2-HG synthesis and away from other cellular processes.[95]

METABOLISM-MEDIATED THERAPEUTICS IN MUTANT IDH CANCERS

The specificity of metabolic changes in mutIDH1 or mutIDH2 cancers and the apparent lack of a critical metabolic role of *R*-2-HG in WT IDH cells, means mutIDHs are promising medicinal chemistry targets. Multiple small-molecule inhibitors have been developed to target mutIDH, and there are several clinical trials underway for treatment of glioma, AML, chondrosarcoma, and ICC (ClinicalTrials.gov: NCT03564821, NCT03515512, NCT03471260, NCT03383575, NCT03343197, NCT02746081, NCT02073394, NCT03684811, NCT03683433, NCT03127735, NCT02977689, NCT02677922).[63,246] First-generation therapeutic mutIDH-selective inhibitors are effective in reducing *R*-2-HG levels *in vivo* and were approved for AML treatment in 2018.[119,247] For solid tumors, promising initial results from clinical trials have been reported for advanced cholangiocarcinoma[246] and glioma.[248] Individuals with advanced mutIDH1 cholangiocarcinomas treated with the mutIDH1 inhibitor ivosidenib report significantly increased progression-free survival (PFS) ($p < 0.0001$) and improved overall survival,[246] whereas a different trial of ivosidenib in advanced mutIDH1 gliomas reported improved disease control and reduced tumor growth.[248]

A variety of mutIDH1 and mutIDH2 inhibitors substantially decrease *R*-2-HG levels in *in vitro* and xenograft models[111,114,202,203,249–253] and individuals with glioma[254] and AML.[119,247,255,256] Some of these inhibitors have been reported to initiate differentiation in AML and glioma cell lines and mouse models,[250,251] but do not necessarily slow growth for all types of glioma or chondrosarcoma cells.[111,203] Resistance has been reported for these first-generation inhibitors,[117–120] which is generally categorized as primary or acquired and *R*-2-HG restoring or non-restoring.[117,118,120] Primary resistance to ivosidenib and enasidenib (i.e., where non-restoration of *R*-2-HG levels is manifest) has been reported in individuals with AML. The non-responding individuals had a higher mutational burden compared with responders, either as baseline mutations in genes of the receptor-tyrosine kinase (RTK) pathway[120] or of the rat sarcoma virus (RAS) pathway.[119] Two different types of acquired *R*-2-HG-restoring mechanisms are described in the literature. The first type relates to second-site mutations that are proposed to reduce the binding affinity of the allosteric inhibitors enasidenib[117] and ivosidenib[120] in mutIDH2 and mutIDH1, respectively. The second type of acquired *R*-2-HG-restoring mechanism is emergence of the "opposite" *IDH* mutation (isoform switching); i.e., mutIDH1 arising in individuals previously with mutIDH2 or vice versa.[118,120]

Altered metabolism in mutIDH1 and mutIDH2 cancer cells after inhibitor treatment, beyond modulation of *R*-2-HG, has received limited attention to date. Two studies, each using cultured glioma cell lines (U87 and/or NHA, mutIDH1^{R132H}) and NMR, confirm that *R*-2-HG levels are significantly decreased

upon treatment with AG5198,[114] AG-120, or AG-881.[252] There is otherwise not necessarily a high degree of agreement between these two studies with regard to changes in other metabolite levels. Lactate has been reported as unchanged[252] or significantly reduced[114] upon treatment. Glutamate has been reported as being significantly increased after treatment ($p < 0.001$),[252] in addition to a concomitant increase in flux from glutamine to glutamate and a decreased flux from glutamine to *R*-2-HG.[252] The second study does not report a significant change in glutamate levels.[114] The difference in glutamate response to treatment is potentially due to use of different cell media in the tissue culture experiments (Dulbecco's modified Eagle's medium[252] versus Roswell Park Memorial Institute medium[114]) because the cell line (U87) and analysis method (NMR) were the same. A third study using isogenic mutIDH1$^{R132H/C}$ clones of HCT116 cells reported that reductive carboxylation could not be rescued after treatment with the mutIDH1 inhibitor IDH1iA.[207]

In more clinically relevant models, two further studies investigated the effect of mutIDH1 inhibitors on the wider metabolism of mutIDH1 glioma cells.[253,254] In orthotopic mouse tumors from mutIDH1^{R132H} U87 or mutIDH1 BT257 (astrocytoma) and mutIDH1 SF10417 (oligodendroglioma) derived from affected individuals, both inhibitors (AG-881 and BAY1436032) were able to significantly decrease *R*-2-HG levels and significantly increase glutamate and (the combined MRS signal of) glutamate/glutamine.[253] Interestingly, NAA was significantly increased across all tumors and drug combinations, but only at the first measurement time point after treatment induction (7 days) and not at the final time point (14–15 days). The first measurement was made prior to changes in tumor volume.[253] A clinical trial of the mutIDH1 inhibitor IDH305 in glioma, studying 5 individuals, 1 week of treatment (550 mg/day, orally) led to a significant reduction in 2-HG levels ($p < 0.05$).[254] Furthermore, there was a trend toward increased lactate levels and an inverse correlation between glutathione and 2-HG levels. Glutamine/glutamate levels, however, were reported as being unchanged.[254]

Interestingly, several studies have shown that mutIDH1 inhibitor treatment makes mutIDH1 glioma cells less sensitive to radiation therapy and certain DNA damaging chemotherapies.[208,257–259] However, in chondrosarcoma cell lines, no correlation was found between *IDH* mutation status and response to radiation therapy, including in the presence of a mutIDH1 inhibitor.[243] Combination of the mutIDH2 inhibitor enasidenib with all-*trans* retinoic acid (ATRA), which is known to initiate differentiation in hematopoietic progenitor cells,[260] led to increased differentiation in commercially available (mutIDH2^{R140Q} TF-1) AML cells and those derived from affected individuals compared with either drug separately.[261] The combination of mutIDH1/2 inhibitors with other types of therapy is likely to be highly dependent on cancer type. A better understanding of the wider biochemical effects of IDH inhibitors on cells is needed and may lead to more effective combinations of mutIDH1/2 inhibitors with other therapies.

There has been some interest in alternative therapeutic approaches that take advantage of metabolic vulnerabilities in mutIDH,[262] such as where a particular cancer type is reliant on a specific metabolic pathway. For example, the apparent reliance of mutIDH1 cells on glutamine has been explored. Treatment with GLS inhibitors showed a greater reduction in viability for mutIDH1 compared with WT IDH1 glioma and AML cells.[198,227,263,264] There is also an ongoing clinical trial using a GLS inhibitor (CB-839/telaglenastat) combined with radiation therapy and temozolomide for treatment of astrocytoma with mutIDH1 or mutIDH2 (ClinicalTrials.gov: NCT03528642). GLS inhibitors have received attention as an adjuvant drug to more traditional chemotherapy in other cancers too, and telaglenastat is generally well tolerated.[265–268] In advanced/metastatic renal cell carcinoma (RCC), telaglenastat in combination with everolimus (a mammalian target of rapamycin[mTOR] inhibitor[269]) improved PFS[268], but did not have a similar effect when paired with cabozantinib (a tyrosine kinase inhibitor[270]). As a single-agent treatment, it appears that telaglenastat stabilizes disease rather than being cytotoxic.[265] Finally, the use of GLS inhibitors in general would benefit from stratification of affected individuals to ensure that genetic mutations that confer vulnerability to glutamine starvation are present.[271,272]

Other drugs with promising mutIDH1 cell-targeting effects are being explored; e.g., repurposing of metformin, phenformin, and chloroquine.[192,264] Chloroquine, best known as an antimalarial agent[273] and autophagy inhibitor,[274] is also capable of inhibiting nerve-specific GLUD2.[275] MutIDH1 glioma cells are likely reliant on GLUD2 for glutamate-dependent anaplerosis of the TCA cycle[104,221] and express GLUD2 at significantly higher levels than WT IDH1 glioma.[104,212,213,221,222] Treatment with chloroquine could potentially render mutIDH1 glioma cells more metabolically vulnerable by limiting their ability to utilize glutamate. Extracellular glutamate has been reported to increase redox potential in mutIDH1 glioma cells,[112] and chloroquine could combine synergistically with a treatment that applies oxidative stress to cells; e.g., radiation therapy. A preclinical study using WT IDH1 stem-like glioma cells demonstrated that treatment with chloroquine during radiation significantly increased cell death; however, in this context, it was considered to be due to the autophagy inhibitory effects of chloroquine.[276] Cells derived from individuals with AML showed large variations in sensitivity towards chloroquine treatment, indicating that, similarly to telaglenastat, stratification of affected individuals is likely necessary for effective chloroquine treatment.[277]

Metformin is commonly used for treatment of type 2 diabetes (T2D)[278] and has emerged as a promising anticancer drug after epidemiological studies revealed reduced cancer risk in individuals using metformin to treat T2D.[279,280] *In vitro* and *in vivo* studies with a variety of cancers have demonstrated that metformin suppresses growth of cancer cells[281] and can have a synergistic effect with other therapies,[282] including WT DH1/2 glioma[283] and AML cells.[284] It is thought that the antiproliferative effects of metformin are in part mediated through activation of AMP-activated phosphate kinase (AMPK).[285] Metformin has been reported to reduce the cell viability of KI mutIDH1^{R132H} breast cancer cells (MCF10A), amplified by concomitant treatment with the GLS inhibitor bis-2-(5-phenylacetamido-1,2,4-thiadiazol-2-yl)ethyl sulfide (BPTES) or the mutIDH1 inhibitor AGI5198.[264] A phase 1b clinical trial targeting mutIDH-bearing solid tumors (glioma, chondrosarcoma, and ICC) with a combination of metformin and chloroquine was well tolerated[286] but showed a lack of clinical response, potentially

because of low intracellular levels of metformin. This led to the more cell-permeable phenformin being proposed as an alternative to metformin.[286]

Combination treatment strategies are a potentially important means of exploiting specific metabolic vulnerabilities in particular cancer types. They can also be used to augment conventional therapies and target cancer-specific metabolic adaptations resulting from conventional treatments. The latter has not been investigated to a significant extent with respect to mutIDH inhibitor treatment but could provide an additional therapeutic approach for clinical studies. The reliance of mutIDH1 glioma cells on pyruvate for anaplerosis of the TCA cycle via PC[177,219] is a potential avenue for metabolism-based therapies. Reliance on PC has been demonstrated for breast[287] and non-small cell lung cancer[288] as well as glutamine-deprived GBM cells (LN229 and SF188)[289] and therefore merits further investigation using combination therapeutics that could target glutamine/glutamate reliance and PC simultaneously. Finally, the increased expression of LDHB in mutIDH1^{R132H} glioma,[104,212,213,217] the enzyme that converts lactate to pyruvate, is also of interest. Silencing the LDHB gene with small interfering RNA has been shown to reduce cell growth in a number of cancer cell lines, including WT IDH1 glioma.[290] LDHB promotes autophagy in a variety of cancer cell lines,[290,291] which can enable advanced solid tumors to recycle intracellular components and alleviate metabolic stress.[292]

CONCLUSIONS

Mutations in genes encoding for *IDH1/2* can lead to remarkably high intracellular and extracellular *R*-2-HG levels, accompanied by apparently wide-ranging, likely context-dependent effects on metabolism and redox homeostasis. Comprehensive studies reporting metabolic changes with respect to *mutIDH* have mainly focused on glioma, despite the availability of cell lines with stable mutIDH1/2 expression for several other cancers with high rates of *IDH1/2* mutations; e.g., AML, chondrosarcoma, and ICC. It has been proposed that many of the metabolic changes observed in *mutIDH* cells are a consequence of elevated *R*-2-HG, in particular via inhibition of specific enzymes, but direct evidence for this is only available in a relatively small number of cases.

Glioma cells harboring mutIDH1 appear to be less glycolytic and rely on oxidative phosphorylation to a greater extent than WT IDH1 glioma cells. Altered metabolic flux in *IDH* mutant cells appears to compensate for reduced production of NADPH via WT IDH1/2 and increased consumption by mutIDH1/2. However, the consumption of NADPH by mutIDH1/2 extends beyond upregulation of the PPP, and the compensatory mechanisms are poorly understood. Glutathione metabolism is also modulated with likely pleiotropic effects on redox chemistry in cells. The evidence suggests that mutIDH1/2-mediated modulation of redox homeostasis is context dependent, varying with cancer type, an observation that is relevant when considering relevant therapies. Amino acid and lipid metabolism are often reported to be altered in mutIDH1/2 cancer cells, but the type and extent of changes appears to be highly context and disease model dependent; a better understanding of what drives changes in amino acids levels in mutIDH1/2 cells is needed.

Selective inhibition of mutIDH1 or mutIDH2 has been demonstrated as a chemically and biologically tractable therapeutic approach, and inhibition of mutIDH1/2 leads to a clear reduction in *R*-2-HG levels *in vitro* and *in vivo*. In terms of benefit, the inhibitors have mainly been tested for efficacy on individuals with more advanced disease and provide relief from disease progression. However, resistance to approved inhibitors has also now been reported, including isoform switching between mutIDH1/2 and mutations leading to reduced efficacy of the allosteric inhibitors. To date, relatively little focus has been given to targeting metabolic vulnerabilities other than elevated *R*-2-HG, despite their prevalence in *IDH1/2* mutant cells compared with WT IDH1/2 cells. This is likely in part due to a lack of consistency across different models and how well the models reflect relevant disease-specific targets. Despite these uncertainties, therapeutically promising metabolic vulnerabilities in *IDH1/2* mutant cells include a greater reliance on altered redox homeostasis, glutamate anaplerosis, and lactate transport and conversion to pyruvate.

A significant amount of research revealing altered metabolism in *mutIDH* cells has been conducted to date, but there is a need for further insights to better understand how metabolic changes are causally linked to specific tumorigenic mechanisms in *mutIDH* cells. It therefore remains to be determined whether pursuing direct inhibition of the mutIDH1/2 enzymes alone using specific inhibitors, or combining these with modulation of additional metabolic targets, will lead to the most effective therapeutic approach for treatment of individuals with mutIDH1/2 cancers.

SUPPLEMENTAL INFORMATION

Supplemental information can be found online at https://doi.org/10.1016/j.xcrm.2021.100469.

ACKNOWLEDGMENTS

I.C.H. thanks the Anne Grete Eidsvig and Kjell Inge Røkke's Foundation for Education for an Aker Scholarship. This work was funded in whole or in part by the Wellcome Trust (106244/Z/14/Z) and CRUK (C8717/A18245). The graphical abstract created with BioRender.

DECLARATION OF INTERESTS

The authors declare no competing interests.

REFERENCES

1. Fouad, Y.A., and Aanei, C. (2017). Revisiting the hallmarks of cancer. Am. J. Cancer Res. *7*, 1016–1036.
2. DeBerardinis, R.J., and Chandel, N.S. (2016). Fundamentals of cancer metabolism. Sci. Adv. *2*, e1600200.
3. Sciacovelli, M., and Frezza, C. (2016). Oncometabolites: Unconventional triggers of oncogenic signalling cascades. Free Radic. Biol. Med. *100*, 175–181.
4. Schmidt, C., Sciacovelli, M., and Frezza, C. (2020). Fumarate hydratase in cancer: A multifaceted tumour suppressor. Semin. Cell Dev. Biol. *98*, 15–25.
5. Yan, H., Parsons, D.W., Jin, G., McLendon, R., Rasheed, B.A., Yuan, W., Kos, I., Batinic-Haberle, I., Jones, S., Riggins, G.J., et al. (2009). IDH1 and IDH2 mutations in gliomas. N. Engl. J. Med. *360*, 765–773.

6. Zhao, S., Lin, Y., Xu, W., Jiang, W., Zha, Z., Wang, P., Yu, W., Li, Z., Gong, L., Peng, Y., et al. (2009). Glioma-derived mutations in IDH1 dominantly inhibit IDH1 catalytic activity and induce HIF-1α. Science *324*, 261–265.

7. Dang, L., White, D.W., Gross, S., Bennett, B.D., Bittinger, M.A., Driggers, E.M., Fantin, V.R., Jang, H.G., Jin, S., Keenan, M.C., et al. (2009). Cancer-associated IDH1 mutations produce 2-hydroxyglutarate. Nature *462*, 739–744.

8. Ward, P.S., Patel, J., Wise, D.R., Abdel-Wahab, O., Bennett, B.D., Coller, H.A., Cross, J.R., Fantin, V.R., Hedvat, C.V., Perl, A.E., et al. (2010). The common feature of leukemia-associated IDH1 and IDH2 mutations is a neomorphic enzyme activity converting α-ketoglutarate to 2-hydroxyglutarate. Cancer Cell *17*, 225–234.

9. Gregersen, N., Ingerslev, J., and Rasmussen, K. (1977). Low molecular weight organic acids in the urine of the newborn. Acta Paediatr. Scand. *66*, 85–89.

10. Hoffmann, G., Aramaki, S., Blum-Hoffmann, E., Nyhan, W.L., and Sweetman, L. (1989). Quantitative analysis for organic acids in biological samples: batch isolation followed by gas chromatographic-mass spectrometric analysis. Clin. Chem. *35*, 587–595.

11. Janin, M., Mylonas, E., Saada, V., Micol, J.-B., Renneville, A., Quivoron, C., Koscielny, S., Scourzic, L., Forget, S., Pautas, C., et al. (2014). Serum 2-hydroxyglutarate production in IDH1- and IDH2-mutated de novo acute myeloid leukemia: a study by the Acute Leukemia French Association group. J. Clin. Oncol. *32*, 297–305.

12. Dang, L., and Su, S.M. (2017). Isocitrate Dehydrogenase Mutation and (R)-2-Hydroxyglutarate: From Basic Discovery to Therapeutics Development. Annu. Rev. Biochem. *86*, 305–331.

13. Balss, J., Meyer, J., Mueller, W., Korshunov, A., Hartmann, C., and von Deimling, A. (2008). Analysis of the IDH1 codon 132 mutation in brain tumors. Acta Neuropathol. *116*, 597–602.

14. Parsons, D.W., Jones, S., Zhang, X., Lin, J.C.-H., Leary, R.J., Angenendt, P., Mankoo, P., Carter, H., Siu, I.-M., Gallia, G.L., et al. (2008). An integrated genomic analysis of human glioblastoma multiforme. Science *321*, 1807–1812.

15. Hartmann, C., Meyer, J., Balss, J., Capper, D., Mueller, W., Christians, A., Felsberg, J., Wolter, M., Mawrin, C., Wick, W., et al. (2009). Type and frequency of IDH1 and IDH2 mutations are related to astrocytic and oligodendroglial differentiation and age: a study of 1,010 diffuse gliomas. Acta Neuropathol. *118*, 469–474.

16. Ichimura, K., Pearson, D.M., Kocialkowski, S., Bäcklund, L.M., Chan, R., Jones, D.T.W., and Collins, V.P. (2009). IDH1 mutations are present in the majority of common adult gliomas but rare in primary glioblastomas. Neuro-oncol. *11*, 341–347.

17. Watanabe, T., Nobusawa, S., Kleihues, P., and Ohgaki, H. (2009). IDH1 mutations are early events in the development of astrocytomas and oligodendrogliomas. Am. J. Pathol. *174*, 1149–1153.

18. Wang, H.-Y., Tang, K., Liang, T.-Y., Zhang, W.-Z., Li, J.-Y., Wang, W., Hu, H.-M., Li, M.-Y., Wang, H.-Q., He, X.-Z., et al. (2016). The comparison of clinical and biological characteristics between IDH1 and IDH2 mutations in gliomas. J. Exp. Clin. Cancer Res. *35*, 86.

19. Amary, M.F., Bacsi, K., Maggiani, F., Damato, S., Halai, D., Berisha, F., Pollock, R., O'Donnell, P., Grigoriadis, A., Diss, T., et al. (2011). IDH1 and IDH2 mutations are frequent events in central chondrosarcoma and central and periosteal chondromas but not in other mesenchymal tumours. J. Pathol. *224*, 334–343.

20. Arai, M., Nobusawa, S., Ikota, H., Takemura, S., and Nakazato, Y. (2012). Frequent IDH1/2 mutations in intracranial chondrosarcoma: a possible diagnostic clue for its differentiation from chordoma. Brain Tumor Pathol. *29*, 201–206.

21. Lu, C., Venneti, S., Akalin, A., Fang, F., Ward, P.S., Dematteo, R.G., Intlekofer, A.M., Chen, C., Ye, J., Hameed, M., et al. (2013). Induction of sarcomas by mutant IDH2. Genes Dev. *27*, 1986–1998.

22. Kato Kaneko, M., Liu, X., Oki, H., Ogasawara, S., Nakamura, T., Saidoh, N., Tsujimoto, Y., Matsuyama, Y., Uruno, A., Sugawara, M., et al. (2014). Isocitrate dehydrogenase mutation is frequently observed in giant cell tumor of bone. Cancer Sci. *105*, 744–748.

23. Jin, Y., Elalaf, H., Watanabe, M., Tamaki, S., Hineno, S., Matsunaga, K., Woltjen, K., Kobayashi, Y., Nagata, S., Ikeya, M., et al. (2015). Mutant IDH1 Dysregulates the Differentiation of Mesenchymal Stem Cells in Association with Gene-Specific Histone Modifications to Cartilage- and Bone-Related Genes. PLoS ONE *10*, e0131998.

24. Lugowska, I., Mikula, M., Teterycz, P., Kulecka, M., Kluska, A., Piatkowska, M., Balabas, A., Wagrodzki, M., Pienkowski, A., Rutkowski, P., et al. (2016). IDH1/2 mutations to predict shorter survival in chondrosarcoma. J. Clin. Oncol. *34*, 11024.

25. Cleven, A.H.G., Suijker, J., Agrogiannis, G., Briaire-de Bruijn, I.H., Frizzell, N., Hoekstra, A.S., Wijers-Koster, P.M., Cleton-Jansen, A.M., and Bovée, J.V.M.G. (2017). *IDH1* or *-2* mutations do not predict outcome and do not cause loss of 5-hydroxymethylcytosine or altered histone modifications in central chondrosarcomas. Clin. Sarcoma Res. *7*, 8.

26. Tallegas, M., Miquelestorena-Standley, É., Labit-Bouvier, C., Badoual, C., Francois, A., Gomez-Brouchet, A., Aubert, S., Collin, C., Tallet, A., and de Pinieux, G. (2019). IDH mutation status in a series of 88 head and neck chondrosarcomas: different profile between tumors of the skull base and tumors involving the facial skeleton and the laryngotracheal tract. Hum. Pathol. *84*, 183–191.

27. Mardis, E.R., Ding, L., Dooling, D.J., Larson, D.E., McLellan, M.D., Chen, K., Koboldt, D.C., Fulton, R.S., Delehaunty, K.D., McGrath, S.D., et al. (2009). Recurring mutations found by sequencing an acute myeloid leukemia genome. N. Engl. J. Med. *361*, 1058–1066.

28. Abbas, S., Lugthart, S., Kavelaars, F.G., Schelen, A., Koenders, J.E., Zeilemaker, A., van Putten, W.J.L., Rijneveld, A.W., Löwenberg, B., and Valk, P.J.M. (2010). Acquired mutations in the genes encoding IDH1 and IDH2 both are recurrent aberrations in acute myeloid leukemia: prevalence and prognostic value. Blood *116*, 2122–2126.

29. Marcucci, G., Maharry, K., Wu, Y.-Z., Radmacher, M.D., Mrózek, K., Margeson, D., Holland, K.B., Whitman, S.P., Becker, H., Schwind, S., et al. (2010). IDH1 and IDH2 gene mutations identify novel molecular subsets within de novo cytogenetically normal acute myeloid leukemia: a Cancer and Leukemia Group B study. J. Clin. Oncol. *28*, 2348–2355.

30. Schnittger, S., Haferlach, C., Ulke, M., Alpermann, T., Kern, W., and Haferlach, T. (2010). *IDH1* mutations are detected in 6.6% of 1414 AML patients and are associated with intermediate risk karyotype and unfavorable prognosis in adults younger than 60 years and unmutated *NPM1* status. Blood *116*, 5486–5496.

31. Wagner, K., Damm, F., Göhring, G., Görlich, K., Heuser, M., Schäfer, I., Ottmann, O., Lübbert, M., Heit, W., Kanz, L., et al. (2010). Impact of IDH1 R132 mutations and an IDH1 single nucleotide polymorphism in cytogenetically normal acute myeloid leukemia: SNP rs11554137 is an adverse prognostic factor. J. Clin. Oncol. *28*, 2356–2364.

32. Molenaar, R.J., Thota, S., Nagata, Y., Patel, B., Clemente, M., Przychodzen, B., Hirsh, C., Viny, A.D., Hosano, N., Bleeker, F.E., et al. (2015). Clinical and biological implications of ancestral and non-ancestral IDH1 and IDH2 mutations in myeloid neoplasms. Leukemia *29*, 2134–2142.

33. Figueroa, M.E., Abdel-Wahab, O., Lu, C., Ward, P.S., Patel, J., Shih, A., Li, Y., Bhagwat, N., Vasanthakumar, A., Fernandez, H.F., et al. (2010). Leukemic IDH1 and IDH2 mutations result in a hypermethylation phenotype, disrupt TET2 function, and impair hematopoietic differentiation. Cancer Cell *18*, 553–567.

34. Paschka, P., Schlenk, R.F., Gaidzik, V.I., Habdank, M., Krönke, J., Bullinger, L., Späth, D., Kayser, S., Zucknick, M., Götze, K., et al. (2010). IDH1 and IDH2 mutations are frequent genetic alterations in acute myeloid leukemia and confer adverse prognosis in cytogenetically normal acute myeloid leukemia with NPM1 mutation without FLT3 internal tandem duplication. J. Clin. Oncol. *28*, 3636–3643.

CellPress
OPEN ACCESS

35. Borger, D.R., Tanabe, K.K., Fan, K.C., Lopez, H.U., Fantin, V.R., Straley, K.S., Schenkein, D.P., Hezel, A.F., Ancukiewicz, M., Liebman, H.M., et al. (2012). Frequent mutation of isocitrate dehydrogenase (IDH)1 and IDH2 in cholangiocarcinoma identified through broad-based tumor genotyping. Oncologist *17*, 72–79.

36. Kipp, B.R., Voss, J.S., Kerr, S.E., Barr Fritcher, E.G., Graham, R.P., Zhang, L., Highsmith, W.E., Zhang, J., Roberts, L.R., Gores, G.J., and Halling, K.C. (2012). Isocitrate dehydrogenase 1 and 2 mutations in cholangiocarcinoma. Hum. Pathol. *43*, 1552–1558.

37. Wang, P., Dong, Q., Zhang, C., Kuan, P.F., Liu, Y., Jeck, W.R., Andersen, J.B., Jiang, W., Savich, G.L., Tan, T.X., et al. (2013). Mutations in isocitrate dehydrogenase 1 and 2 occur frequently in intrahepatic cholangiocarcinomas and share hypermethylation targets with glioblastomas. Oncogene *32*, 3091–3100.

38. Jiao, Y., Pawlik, T.M., Anders, R.A., Selaru, F.M., Streppel, M.M., Lucas, D.J., Niknafs, N., Guthrie, V.B., Maitra, A., Argani, P., et al. (2013). Exome sequencing identifies frequent inactivating mutations in BAP1, ARID1A and PBRM1 in intrahepatic cholangiocarcinomas. Nat. Genet. *45*, 1470–1473.

39. Ross, J.S., Wang, K., Gay, L., Al-Rohil, R., Rand, J.V., Jones, D.M., Lee, H.J., Sheehan, C.E., Otto, G.A., Palmer, G., et al. (2014). New routes to targeted therapy of intrahepatic cholangiocarcinomas revealed by next-generation sequencing. Oncologist *19*, 235–242.

40. Farshidfar, F., Zheng, S., Gingras, M.-C., Newton, Y., Shih, J., Robertson, A.G., Hinoue, T., Hoadley, K.A., Gibb, E.A., Roszik, J., et al.; Cancer Genome Atlas Network (2017). Integrative Genomic Analysis of Cholangiocarcinoma Identifies Distinct IDH-Mutant Molecular Profiles. Cell Rep. *18*, 2780–2794.

41. Lee, J.H., Shin, D.H., Park, W.Y., Shin, N., Kim, A., Lee, H.J., Kim, Y.K., Choi, K.U., Kim, J.Y., Yang, Y.I., et al. (2017). IDH1 R132C mutation is detected in clear cell hepatocellular carcinoma by pyrosequencing. World J. Surg. Oncol. *15*, 82.

42. Nepal, C., O'Rourke, C.J., Oliveira, D.V.N.P., Taranta, A., Shema, S., Gautam, P., Calderaro, J., Barbour, A., Raggi, C., Wennerberg, K., et al. (2018). Genomic perturbations reveal distinct regulatory networks in intrahepatic cholangiocarcinoma. Hepatology *68*, 949–963.

43. Wang, J., Zhang, Z.G., Ding, Z.Y., Dong, W., Liang, H.F., Chu, L., Zhang, B.X., and Chen, X.P. (2018). IDH1 mutation correlates with a beneficial prognosis and suppresses tumor growth in IHCC. J. Surg. Res. *231*, 116–125.

44. Cairns, R.A., Iqbal, J., Lemonnier, F., Kucuk, C., de Leval, L., Jais, J.-P., Parrens, M., Martin, A., Xerri, L., Brousset, P., et al. (2012). IDH2 mutations are frequent in angioimmunoblastic T-cell lymphoma. Blood *119*, 1901–1903.

45. Odejide, O., Weigert, O., Lane, A.A., Toscano, D., Lunning, M.A., Kopp, N., Kim, S., van Bodegom, D., Bolla, S., Schatz, J.H., et al. (2014). A targeted mutational landscape of angioimmunoblastic T-cell lymphoma. Blood *123*, 1293–1296.

46. Sakata-Yanagimoto, M., Enami, T., Yoshida, K., Shiraishi, Y., Ishii, R., Miyake, Y., Muto, H., Tsuyama, N., Sato-Otsubo, A., Okuno, Y., et al. (2014). Somatic RHOA mutation in angioimmunoblastic T cell lymphoma. Nat. Genet. *46*, 171–175.

47. Wang, C., McKeithan, T.W., Gong, Q., Zhang, W., Bouska, A., Rosenwald, A., Gascoyne, R.D., Wu, X., Wang, J., Muhammad, Z., et al. (2015). $IDH2^{R172}$ mutations define a unique subgroup of patients with angioimmunoblastic T-cell lymphoma. Blood *126*, 1741–1752.

48. Dogan, S., Chute, D.J., Xu, B., Ptashkin, R.N., Chandramohan, R., Casanova-Murphy, J., Nafa, K., Bishop, J.A., Chiosea, S.I., Stelow, E.B., et al. (2017). Frequent IDH2 R172 mutations in undifferentiated and poorly-differentiated sinonasal carcinomas. J. Pathol. *242*, 400–408.

49. Jo, V.Y., Chau, N.G., Hornick, J.L., Krane, J.F., and Sholl, L.M. (2017). Recurrent IDH2 R172X mutations in sinonasal undifferentiated carcinoma. Mod. Pathol. *30*, 650–659.

50. Riobello, C., López-Hernández, A., Cabal, V.N., García-Marín, R., Suárez-Fernández, L., Sánchez-Fernández, P., Vivanco, B., Blanco, V., López, F., Franchi, A., et al. (2020). IDH2 Mutation Analysis in Undifferentiated and Poorly Differentiated Sinonasal Carcinomas for Diagnosis and Clinical Management. Am. J. Surg. Pathol. *44*, 396–405.

51. Chiang, S., Weigelt, B., Wen, H.-C., Pareja, F., Raghavendra, A., Martelotto, L.G., Burke, K.A., Basili, T., Li, A., Geyer, F.C., et al. (2016). *IDH2* Mutations Define a Unique Subtype of Breast Cancer with Altered Nuclear Polarity. Cancer Res. *76*, 7118–7129.

52. Lozada, J.R., Basili, T., Pareja, F., Alemar, B., Paula, A.D.C., Gularte-Merida, R., Giri, D.D., Querzoli, P., Cserni, G., Rakha, E.A., et al. (2018). Solid papillary breast carcinomas resembling the tall cell variant of papillary thyroid neoplasms (solid papillary carcinomas with reverse polarity) harbour recurrent mutations affecting IDH2 and PIK3CA: a validation cohort. Histopathology *73*, 339–344.

53. Louis, D.N., Perry, A., Reifenberger, G., von Deimling, A., Figarella-Branger, D., Cavenee, W.K., Ohgaki, H., Wiestler, O.D., Kleihues, P., and Ellison, D.W. (2016). The 2016 World Health Organization Classification of Tumors of the Central Nervous System: a summary. Acta Neuropathol. *131*, 803–820.

54. Louis, D.N., Perry, A., Wesseling, P., Brat, D.J., Cree, I.A., Figarella-Branger, D., Hawkins, C., Ng, H.K., Pfister, S.M., Reifenberger, G., et al. (2021). The 2021 WHO Classification of Tumors of the Central Nervous System: a summary. Neuro-oncol. *23*, 1231–1251.

55. Pusch, S., Sahm, F., Meyer, J., Mittelbronn, M., Hartmann, C., and von Deimling, A. (2011). Glioma IDH1 mutation patterns off the beaten track. Neuropathol. Appl. Neurobiol. *37*, 428–430.

56. Gupta, R., Flanagan, S., Li, C.C.Y., Lee, M., Shivalingham, B., Maleki, S., Wheeler, H.R., and Buckland, M.E. (2013). Expanding the spectrum of IDH1 mutations in gliomas. Mod. Pathol. *26*, 619–625.

57. Balss, J., Pusch, S., Beck, A.-C., Herold-Mende, C., Krämer, A., Thiede, C., Buckel, W., Langhans, C.-D., Okun, J.G., and von Deimling, A. (2012). Enzymatic assay for quantitative analysis of (D)-2-hydroxyglutarate. Acta Neuropathol. *124*, 883–891.

58. Gross, S., Cairns, R.A., Minden, M.D., Driggers, E.M., Bittinger, M.A., Jang, H.G., Sasaki, M., Jin, S., Schenkein, D.P., Su, S.M., et al. (2010). Cancer-associated metabolite 2-hydroxyglutarate accumulates in acute myelogenous leukemia with isocitrate dehydrogenase 1 and 2 mutations. J. Exp. Med. *207*, 339–344.

59. Kang, M.R., Kim, M.S., Oh, J.E., Kim, Y.R., Song, S.Y., Seo, S.I., Lee, J.Y., Yoo, N.J., and Lee, S.H. (2009). Mutational analysis of IDH1 codon 132 in glioblastomas and other common cancers. Int. J. Cancer *125*, 353–355.

60. Thol, F., Weissinger, E.M., Krauter, J., Wagner, K., Damm, F., Wichmann, M., Göhring, G., Schumann, C., Bug, G., Ottmann, O., et al. (2010). *IDH1* mutations in patients with myelodysplastic syndromes are associated with an unfavorable prognosis. Haematologica *95*, 1668–1674.

61. Tefferi, A., Lasho, T.L., Abdel-Wahab, O., Guglielmelli, P., Patel, J., Caramazza, D., Pieri, L., Finke, C.M., Kilpivaara, O., Wadleigh, M., et al. (2010). IDH1 and IDH2 mutation studies in 1473 patients with chronic-, fibrotic- or blast-phase essential thrombocythemia, polycythemia vera or myelofibrosis. Leukemia *24*, 1302–1309.

62. Pardanani, A., Lasho, T.L., Finke, C.M., Mai, M., McClure, R.F., and Tefferi, A. (2010). IDH1 and IDH2 mutation analysis in chronic- and blast-phase myeloproliferative neoplasms. Leukemia *24*, 1146–1151.

63. Andersson, A.K., Miller, D.W., Lynch, J.A., Lemoff, A.S., Cai, Z., Pounds, S.B., Radtke, I., Yan, B., Schuetz, J.D., Rubnitz, J.E., et al. (2011). IDH1 and IDH2 mutations in pediatric acute leukemia. Leukemia *25*, 1570–1577.

64. Oki, K., Takita, J., Hiwatari, M., Nishimura, R., Sanada, M., Okubo, J., Adachi, M., Sotomatsu, M., Kikuchi, A., Igarashi, T., et al. (2011). IDH1 and IDH2 mutations are rare in pediatric myeloid malignancies. Leukemia *25*, 382–384.

65. Ally, A., Balasundaram, M., Carlsen, R., Chuah, E., Clarke, A., Dhalla, N., Holt, R.A., Jones, S.J.M., Lee, D., Ma, Y., et al.; Cancer Genome Atlas Research Network. Electronic address: wheeler@bcm.edu; Cancer Genome Atlas Research Network (2017). Comprehensive and Integrative Genomic Characterization of Hepatocellular Carcinoma. Cell *169*, 1327–1341.e23.

66. Zhu, G.G., Nafa, K., Agaram, N., Zehir, A., Benayed, R., Sadowska, J., Borsu, L., Kelly, C., Tap, W.D., Fabbri, N., et al. (2020). Genomic Profiling Identifies Association of *IDH1/IDH2* Mutation with Longer Relapse-Free and Metastasis-Free Survival in High-Grade Chondrosarcoma. Clin. Cancer Res. *26*, 419–427.

67. Liu, X., Kato, Y., Kaneko, M.K., Sugawara, M., Ogasawara, S., Tsujimoto, Y., Naganuma, Y., Yamakawa, M., Tsuchiya, T., and Takagi, M. (2013). Isocitrate dehydrogenase 2 mutation is a frequent event in osteosarcoma detected by a multi-specific monoclonal antibody MsMab-1. Cancer Med. *2*, 803–814.

68. Na, K.Y., Noh, B.-J., Sung, J.-Y., Kim, Y.W., Santini Araujo, E., and Park, Y.-K. (2015). IDH Mutation Analysis in Ewing Sarcoma Family Tumors. J. Pathol. Transl. Med. *49*, 257–261.

69. Pansuriya, T.C., van Eijk, R., d'Adamo, P., van Ruler, M.A.J.H., Kuijjer, M.L., Oosting, J., Cleton-Jansen, A.-M., van Oosterwijk, J.G., Verbeke, S.L.J., Meijer, D., et al. (2011). Somatic mosaic IDH1 and IDH2 mutations are associated with enchondroma and spindle cell hemangioma in Ollier disease and Maffucci syndrome. Nat. Genet. *43*, 1256–1261.

70. Amary, M.F., Damato, S., Halai, D., Eskandarpour, M., Berisha, F., Bonar, F., McCarthy, S., Fantin, V.R., Straley, K.S., Lobo, S., et al. (2011). Ollier disease and Maffucci syndrome are caused by somatic mosaic mutations of IDH1 and IDH2. Nat. Genet. *43*, 1262–1265.

71. Fathi, A.T., Sadrzadeh, H., Comander, A.H., Higgins, M.J., Bardia, A., Perry, A., Burke, M., Silver, R., Matulis, C.R., Straley, K.S., et al. (2014). Isocitrate dehydrogenase 1 (IDH1) mutation in breast adenocarcinoma is associated with elevated levels of serum and urine 2-hydroxyglutarate. Oncologist *19*, 602–607.

72. Li-Chang, H.H., Kasaian, K., Ng, Y., Lum, A., Kong, E., Lim, H., Jones, S.J., Huntsman, D.G., Schaeffer, D.F., and Yip, S. (2015). Retrospective review using targeted deep sequencing reveals mutational differences between gastroesophageal junction and gastric carcinomas. BMC Cancer *15*, 32.

73. Hartman, D.J., Binion, D., Regueiro, M., Schraut, W., Bahary, N., Sun, W., Nikiforova, M., and Pai, R.K. (2014). Isocitrate dehydrogenase-1 is mutated in inflammatory bowel disease-associated intestinal adenocarcinoma with low-grade tubuloglandular histology but not in sporadic intestinal adenocarcinoma. Am. J. Surg. Pathol. *38*, 1147–1156.

74. Lopez, G.Y., Reitman, Z.J., Solomon, D., Waldman, T., Bigner, D.D., McLendon, R.E., Rosenberg, S.A., Samuels, Y., and Yan, H. (2010). IDH1(R132) mutation identified in one human melanoma metastasis, but not correlated with metastases to the brain. Biochem. Biophys. Res. Commun. *398*, 585–587.

75. Toth, L.N., de Abreu, F.B., and Tafe, L.J. (2018). Non-small cell lung cancers with isocitrate dehydrogenase 1 or 2 (IDH1/2) mutations. Hum. Pathol. *78*, 138–143.

76. Gaal, J., Burnichon, N., Korpershoek, E., Roncelin, I., Bertherat, J., Plouin, P.F., de Krijger, R.R., Gimenez-Roqueplo, A.P., and Dinjens, W.N. (2010). Isocitrate dehydrogenase mutations are rare in pheochromocytomas and paragangliomas. J. Clin. Endocrinol. Metab. *95*, 1274–1278.

77. Hinsch, A., Brolund, M., Hube-Magg, C., Kluth, M., Simon, R., Möller-Koop, C., Sauter, G., Steurer, S., Luebke, A., Angerer, A., et al. (2018). Immunohistochemically detected IDH1^{R132H} mutation is rare and mostly heterogeneous in prostate cancer. World J. Urol. *36*, 877–882.

78. Kurek, K.C., Pansuriya, T.C., van Ruler, M.A.J.H., van den Akker, B., Luks, V.L., Verbeke, S.L.J., Kozakewich, H.P., Sciot, R., Lev, D., Lazar, A.J., et al. (2013). R132C IDH1 mutations are found in spindle cell hemangiomas and not in other vascular tumors or malformations. Am. J. Pathol. *182*, 1494–1500.

79. Murugan, A.K., Bojdani, E., and Xing, M. (2010). Identification and functional characterization of isocitrate dehydrogenase 1 (IDH1) mutations in thyroid cancer. Biochem. Biophys. Res. Commun. *393*, 555–559.

80. Hemerly, J.P., Bastos, A.U., and Cerutti, J.M. (2010). Identification of several novel non-p.R132 IDH1 variants in thyroid carcinomas. Eur. J. Endocrinol. *163*, 747–755.

81. Rakheja, D., Mitui, M., Boriack, R.L., and DeBerardinis, R.J. (2011). Isocitrate dehydrogenase 1/2 mutational analyses and 2-hydroxyglutarate measurements in Wilms tumors. Pediatr. Blood Cancer *56*, 379–383.

82. Cadoux-Hudson, Thomas, Schofield, Christopher, McCullagh, James, et al. (2021). Isocitrate dehydrogenase gene variants in cancer and their clinical significance. Biochemical Society Transactions. https://doi.org/10.1042/BST20210277.

83. Suzuki, H., Aoki, K., Chiba, K., Sato, Y., Shiozawa, Y., Shiraishi, Y., Shimamura, T., Niida, A., Motomura, K., Ohka, F., et al. (2015). Mutational landscape and clonal architecture in grade II and III gliomas. Nat. Genet. *47*, 458–468.

84. Abdel-Wahab, O., Manshouri, T., Patel, J., Harris, K., Yao, J., Hedvat, C., Heguy, A., Bueso-Ramos, C., Kantarjian, H., Levine, R.L., and Verstovsek, S. (2010). Genetic analysis of transforming events that convert chronic myeloproliferative neoplasms to leukemias. Cancer Res. *70*, 447–452.

85. Xie, M., Lu, C., Wang, J., McLellan, M.D., Johnson, K.J., Wendl, M.C., McMichael, J.F., Schmidt, H.K., Yellapantula, V., Miller, C.A., et al. (2014). Age-related mutations associated with clonal hematopoietic expansion and malignancies. Nat. Med. *20*, 1472–1478.

86. Chowdhury, R., Yeoh, K.K., Tian, Y.M., Hillringhaus, L., Bagg, E.A., Rose, N.R., Leung, I.K.H., Li, X.S., Woon, E.C.Y., Yang, M., et al. (2011). The oncometabolite 2-hydroxyglutarate inhibits histone lysine demethylases. EMBO Rep. *12*, 463–469.

87. Xu, W., Yang, H., Liu, Y., Yang, Y., Wang, P., Kim, S.-H., Ito, S., Yang, C., Wang, P., Xiao, M.-T., et al. (2011). Oncometabolite 2-hydroxyglutarate is a competitive inhibitor of α-ketoglutarate-dependent dioxygenases. Cancer Cell *19*, 17–30.

88. Ye, D., Guan, K.-L., and Xiong, Y. (2018). Metabolism, Activity, and Targeting of D- and L-2-Hydroxyglutarates. Trends Cancer *4*, 151–165.

89. Johannessen, T.A., Mukherjee, J., Viswanath, P., Ohba, S., Ronen, S.M., Bjerkvig, R., and Pieper, R.O. (2016). Rapid Conversion of Mutant IDH1 from Driver to Passenger in a Model of Human Gliomagenesis. Mol. Cancer Res. *14*, 976–983.

90. Walker, O.S., Elsässer, S.J., Mahesh, M., Bachman, M., Balasubramanian, S., and Chin, J.W. (2016). Photoactivation of Mutant Isocitrate Dehydrogenase 2 Reveals Rapid Cancer-Associated Metabolic and Epigenetic Changes. J. Am. Chem. Soc. *138*, 718–721.

91. Mazor, T., Chesnelong, C., Pankov, A., Jalbert, L.E., Hong, C., Hayes, J., Smirnov, I.V., Marshall, R., Souza, C.F., Shen, Y., et al. (2017). Clonal expansion and epigenetic reprogramming following deletion or amplification of mutant *IDH1*. Proc. Natl. Acad. Sci. USA *114*, 10743–10748.

92. Chaturvedi, A., Goparaju, R., Gupta, C., Weder, J., Klünemann, T., Araujo Cruz, M.M., Kloos, A., Goerlich, K., Schottmann, R., Othman, B., et al. (2020). In vivo efficacy of mutant IDH1 inhibitor HMS-101 and structural resolution of distinct binding site. Leukemia *34*, 416–426.

93. Li, S., Chen, X., Wang, J., Meydan, C., Glass, J.L., Shih, A.H., Delwel, R., Levine, R.L., Mason, C.E., and Melnick, A.M. (2020). Somatic Mutations Drive Specific, but Reversible, Epigenetic Heterogeneity States in AML. Cancer Discov. *10*, 1934–1949.

94. Wang, F., Morita, K., DiNardo, C.D., Furudate, K., Tanaka, T., Yan, Y., Patel, K.P., MacBeth, K.J., Wu, B., Liu, G., et al. (2021). Leukemia stemness and co-occurring mutations drive resistance to IDH inhibitors in acute myeloid leukemia. Nat. Commun. *12*, 2607.

CellPress
OPEN ACCESS

95. Badur, M.G., Muthusamy, T., Parker, S.J., Ma, S., McBrayer, S.K., Cordes, T., Magana, J.H., Guan, K.-L., and Metallo, C.M. (2018). Oncogenic R132 IDH1 mutations limit NADPH for de novo lipogenesis through (D)2-Hydroxyglutarate production in fibrosarcoma cells. Cell Rep. *25*, 1680.

96. Biedermann, J., Preussler, M., Conde, M., Peitzsch, M., Richter, S., Wiedemuth, R., Abou-El-Ardat, K., Krüger, A., Meinhardt, M., Schackert, G., et al. (2019). Mutant IDH1 Differently Affects Redox State and Metabolism in Glial Cells of Normal and Tumor Origin. Cancers (Basel) *11*, 2028.

97. Bleeker, F.E., Atai, N.A., Lamba, S., Jonker, A., Rijkeboer, D., Bosch, K.S., Tigchelaar, W., Troost, D., Vandertop, W.P., Bardelli, A., and Van Noorden, C.J. (2010). The prognostic IDH1(R132) mutation is associated with reduced NADP+-dependent IDH activity in glioblastoma. Acta Neuropathol. *119*, 487–494.

98. Esmaeili, M., Hamans, B.C., Navis, A.C., van Horssen, R., Bathen, T.F., Gribbestad, I.S., Leenders, W.P.J., and Heerschap, A. (2014). IDH1 R132H mutation generates a distinct phospholipid metabolite profile in glioma. Cancer Res. *74*, 4898–4907.

99. Fack, F., Tardito, S., Hochart, G., Oudin, A., Zheng, L., Fritah, S., Golebiewska, A., Nazarov, P.V., Bernard, A., Hau, A.C., et al. (2017). Altered metabolic landscape in IDH-mutant gliomas affects phospholipid, energy, and oxidative stress pathways. EMBO Mol. Med. *9*, 1681–1695.

100. Gelman, S.J., Naser, F., Mahieu, N.G., McKenzie, L.D., Dunn, G.P., Chheda, M.G., and Patti, G.J. (2018). Consumption of NADPH for 2-HG synthesis increases pentose phosphate pathway flux and sensitizes cells to oxidative stress. Cell Rep. *22*, 512–522.

101. Hujber, Z., Horváth, G., Petővári, G., Krencz, I., Dankó, T., Mészáros, K., Rajnai, H., Szoboszlai, N., Leenders, W.P.J., Jeney, A., et al. (2018). GABA, glutamine, glutamate oxidation and succinic semialdehyde dehydrogenase expression in human gliomas. J. Exp. Clin. Cancer Res. *37*, 271.

102. Izquierdo-Garcia, J.L., Viswanath, P., Eriksson, P., Chaumeil, M.M., Pieper, R.O., Phillips, J.J., and Ronen, S.M. (2015). Metabolic reprogramming in mutant IDH1 glioma cells. PLoS ONE *10*, e0118781.

103. Jalbert, L.E., Elkhaled, A., Phillips, J.J., Neill, E., Williams, A., Crane, J.C., Olson, M.P., Molinaro, A.M., Berger, M.S., Kurhanewicz, J., et al. (2017). Metabolic Profiling of IDH Mutation and Malignant Progression in Infiltrating Glioma. Sci. Rep. *7*, 44792.

104. Lenting, K., Khurshed, M., Peeters, T.H., van den Heuvel, C.N.A.M., van Lith, S.A.M., de Bitter, T., Hendriks, W., Span, P.N., Molenaar, R.J., Botman, D., et al. (2019). Isocitrate dehydrogenase 1-mutated human gliomas depend on lactate and glutamate to alleviate metabolic stress. FASEB J. *33*, 557–571.

105. Mugoni, V., Panella, R., Cheloni, G., Chen, M., Pozdnyakova, O., Stroopinsky, D., Guarnerio, J., Monteleone, E., Lee, J.D., Mendez, L., et al. (2019). Vulnerabilities in mIDH2 AML confer sensitivity to APL-like targeted combination therapy. Cell Res. *29*, 446–459.

106. Ohka, F., Ito, M., Ranjit, M., Senga, T., Motomura, A., Motomura, K., Saito, K., Kato, K., Kato, Y., Wakabayashi, T., et al. (2014). Quantitative metabolome analysis profiles activation of glutaminolysis in glioma with IDH1 mutation. Tumour Biol. *35*, 5911–5920.

107. Reitman, Z.J., Jin, G., Karoly, E.D., Spasojevic, I., Yang, J., Kinzler, K.W., He, Y., Bigner, D.D., Vogelstein, B., and Yan, H. (2011). Profiling the effects of isocitrate dehydrogenase 1 and 2 mutations on the cellular metabolome. Proc. Natl. Acad. Sci. USA *108*, 3270–3275.

108. Shi, J., Zuo, H., Ni, L., Xia, L., Zhao, L., Gong, M., Nie, D., Gong, P., Cui, D., Shi, W., and Chen, J. (2014). An IDH1 mutation inhibits growth of glioma cells via GSH depletion and ROS generation. Neurol. Sci. *35*, 839–845.

109. Stuani, L., Riols, F., Millard, P., Sabatier, M., Batut, A., Saland, E., Viars, F., Tonini, L., Zaghdoudi, S., Linares, L.K., et al. (2018). Stable Isotope Labeling Highlights Enhanced Fatty Acid and Lipid Metabolism in Human Acute Myeloid Leukemia. Int. J. Mol. Sci. *19*, 3325.

110. Tang, X., Fu, X., Liu, Y., Yu, D., Cai, S.J., and Yang, C. (2020). Blockade of Glutathione Metabolism in *IDH1*-Mutated Glioma. Mol. Cancer Ther. *19*, 221–230.

111. Tateishi, K., Wakimoto, H., Iafrate, A.J., Tanaka, S., Loebel, F., Lelic, N., Wiederschain, D., Bedel, O., Deng, G., Zhang, B., et al. (2015). Extreme Vulnerability of IDH1 Mutant Cancers to NAD+ Depletion. Cancer Cell *28*, 773–784.

112. Tiburcio, P.D.B., Gillespie, D.L., Jensen, R.L., and Huang, L.E. (2020). Extracellular glutamate and $IDH1^{R132H}$ inhibitor promote glioma growth by boosting redox potential. J. Neurooncol. *146*, 427–437.

113. Walsby-Tickle, J., Gannon, J., Hvinden, I., Bardella, C., Abboud, M.I., Nazeer, A., Hauton, D., Pires, E., Cadoux-Hudson, T., Schofield, C.J., and McCullagh, J.S.O. (2020). Anion-exchange chromatography mass spectrometry provides extensive coverage of primary metabolic pathways revealing altered metabolism in IDH1 mutant cells. Commun. Biol. *3*, 247.

114. Wen, H., Cho, H.R., Yun, T., Kim, H., Park, C.K., Lee, S.H., Choi, S.H., and Park, S. (2015). Metabolomic comparison between cells over-expressing isocitrate dehydrogenase 1 and 2 mutants and the effects of an inhibitor on the metabolism. J. Neurochem. *132*, 183–193.

115. Yang, R., Zhao, Y., Gu, Y., Yang, Y., Gao, X., Yuan, Y., Xiao, L., Zhang, J., Sun, C., Yang, H., et al. (2020). Isocitrate dehydrogenase 1 mutation enhances 24(S)-hydroxycholesterol production and alters cholesterol homeostasis in glioma. Oncogene *39*, 6340–6353.

116. Zhou, L., Wang, Z., Hu, C., Zhang, C., Kovatcheva-Datchary, P., Yu, D., Liu, S., Ren, F., Wang, X., Li, Y., et al. (2019). Integrated Metabolomics and Lipidomics Analyses Reveal Metabolic Reprogramming in Human Glioma with IDH1 Mutation. J. Proteome Res. *18*, 960–969.

117. Intlekofer, A.M., Shih, A.H., Wang, B., Nazir, A., Rustenburg, A.S., Albanese, S.K., Patel, M., Famulare, C., Correa, F.M., Takemoto, N., et al. (2018). Acquired resistance to IDH inhibition through trans or cis dimer-interface mutations. Nature *559*, 125–129.

118. Harding, J.J., Lowery, M.A., Shih, A.H., Schvartzman, J.M., Hou, S., Famulare, C., Patel, M., Roshal, M., Do, R.K., Zehir, A., et al. (2018). Isoform Switching as a Mechanism of Acquired Resistance to Mutant Isocitrate Dehydrogenase Inhibition. Cancer Discov. *8*, 1540–1547.

119. Amatangelo, M.D., Quek, L., Shih, A., Stein, E.M., Roshal, M., David, M.D., Marteyn, B., Farnoud, N.R., de Botton, S., Bernard, O.A., et al. (2017). Enasidenib induces acute myeloid leukemia cell differentiation to promote clinical response. Blood *130*, 732–741.

120. Choe, S., Wang, H., DiNardo, C.D., Stein, E.M., de Botton, S., Roboz, G.J., Altman, J.K., Mims, A.S., Watts, J.M., Pollyea, D.A., et al. (2020). Molecular mechanisms mediating relapse following ivosidenib monotherapy in IDH1-mutant relapsed or refractory AML. Blood Adv. *4*, 1894–1905.

121. Wishart, D.S. (2019). Metabolomics for Investigating Physiological and Pathophysiological Processes. Physiol. Rev. *99*, 1819–1875.

122. An, Z., Ganji, S.K., Tiwari, V., Pinho, M.C., Patel, T., Barnett, S., Pan, E., Mickey, B.E., Maher, E.A., and Choi, C. (2017). Detection of 2-hydroxyglutarate in brain tumors by triple-refocusing MR spectroscopy at 3T in vivo. Magn. Reson. Med. *78*, 40–48.

123. Choi, C., Ganji, S.K., DeBerardinis, R.J., Hatanpaa, K.J., Rakheja, D., Kovacs, Z., Yang, X.-L., Mashimo, T., Raisanen, J.M., Marin-Valencia, I., et al. (2012). 2-hydroxyglutarate detection by magnetic resonance spectroscopy in IDH-mutated patients with gliomas. Nat. Med. *18*, 624–629.

124. Emir, U.E., Larkin, S.J., de Pennington, N., Voets, N., Plaha, P., Stacey, R., Al-Qahtani, K., Mccullagh, J., Schofield, C.J., Clare, S., et al. (2016). Noninvasive Quantification of 2-Hydroxyglutarate in Human Gliomas with IDH1 and IDH2 Mutations. Cancer Res. *76*, 43–49.

125. Cairns, R.A., and Mak, T.W. (2013). Oncogenic isocitrate dehydrogenase mutations: mechanisms, models, and clinical opportunities. Cancer Discov. *3*, 730–741.

126. D Adamo, A.F., Jr., and Haft, D.E. (1965). An alternate pathway of α-ketoglutarate catabolism in the isolated, perfused rat liver. I. Studies with DL-glutamate-2- and -5-14C. J. Biol. Chem. *240*, 613–617.

127. Dalziel, K., and Londesborough, J.C. (1968). The mechanisms of reductive carboxylation reactions. Carbon dioxide or bicarbonate as substrate of nicotinamide-adenine dinucleotide phosphate-linked isocitrate dehydrogenase and malic enzyme. Biochem. J. *110*, 223–230.

128. Gabriel, J.L., Zervos, P.R., and Plaut, G.W.E. (1986). Activity of purified NAD-specific isocitrate dehydrogenase at modulator and substrate concentrations approximating conditions in mitochondria. Metabolism *35*, 661–667.

129. Lowenstein, J.M., and Smith, S.R. (1962). Intra- and extramitochondrial isocitrate dehydrogenases. Biochim. Biophys. Acta *56*, 385–387.

130. Geisbrecht, B.V., and Gould, S.J. (1999). The human PICD gene encodes a cytoplasmic and peroxisomal NADP(+)-dependent isocitrate dehydrogenase. J. Biol. Chem. *274*, 30527–30533.

131. Chen, R.F., and Plaut, G.W.E. (1963). Activation and Inhibition of DPN-linked Isocitrate Dehydrogenase of Heart by Certain Nucleotides. Biochemistry *2*, 1023–1032.

132. Plaut, G.W., and Aogaichi, T. (1968). Purification and properties of diphosphopyridine nuleotide-linked isocitrate dehydrogenase of mammalian liver. J. Biol. Chem. *243*, 5572–5583.

133. Koh, H.-J., Lee, S.-M., Son, B.-G., Lee, S.-H., Ryoo, Z.Y., Chang, K.-T., Park, J.-W., Park, D.-C., Song, B.J., Veech, R.L., et al. (2004). Cytosolic NADP+-dependent isocitrate dehydrogenase plays a key role in lipid metabolism. J. Biol. Chem. *279*, 39968–39974.

134. Jo, S.-H., Lee, S.-H., Chun, H.S., Lee, S.M., Koh, H.-J., Lee, S.-E., Chun, J.-S., Park, J.-W., and Huh, T.-L. (2002). Cellular defense against UVB-induced phototoxicity by cytosolic NADP(+)-dependent isocitrate dehydrogenase. Biochem. Biophys. Res. Commun. *292*, 542–549.

135. Kim, S.Y., Lee, S.M., Tak, J.K., Choi, K.S., Kwon, T.K., and Park, J.-W. (2007). Regulation of singlet oxygen-induced apoptosis by cytosolic NADP+-dependent isocitrate dehydrogenase. Mol. Cell. Biochem. *302*, 27–34.

136. Metallo, C.M., Gameiro, P.A., Bell, E.L., Mattaini, K.R., Yang, J., Hiller, K., Jewell, C.M., Johnson, Z.R., Irvine, D.J., Guarente, L., et al. (2011). Reductive glutamine metabolism by IDH1 mediates lipogenesis under hypoxia. Nature *481*, 380–384.

137. Mullen, A.R., Wheaton, W.W., Jin, E.S., Chen, P.-H., Sullivan, L.B., Cheng, T., Yang, Y., Linehan, W.M., Chandel, N.S., and DeBerardinis, R.J. (2011). Reductive carboxylation supports growth in tumour cells with defective mitochondria. Nature *481*, 385–388.

138. Jo, S.-H., Son, M.-K., Koh, H.-J., Lee, S.-M., Song, I.-H., Kim, Y.-O., Lee, Y.-S., Jeong, K.-S., Kim, W.B., Park, J.-W., et al. (2001). Control of mitochondrial redox balance and cellular defense against oxidative damage by mitochondrial NADP+-dependent isocitrate dehydrogenase. J. Biol. Chem. *276*, 16168–16176.

139. Lee, J.H., Kim, S.Y., Kil, I.S., and Park, J.-W. (2007). Regulation of ionizing radiation-induced apoptosis by mitochondrial NADP+-dependent isocitrate dehydrogenase. J. Biol. Chem. *282*, 13385–13394.

140. Wise, D.R., Ward, P.S., Shay, J.E.S., Cross, J.R., Gruber, J.J., Sachdeva, U.M., Platt, J.M., DeMatteo, R.G., Simon, M.C., and Thompson, C.B. (2011). Hypoxia promotes isocitrate dehydrogenase-dependent carboxylation of α-ketoglutarate to citrate to support cell growth and viability. Proc. Natl. Acad. Sci. USA *108*, 19611–19616.

141. Hoffmann, G.F., Seppel, C.K., Holmes, B., Mitchell, L., Christen, H.J., Hanefeld, F., Rating, D., and Nyhan, W.L. (1993). Quantitative organic acid analysis in cerebrospinal fluid and plasma: reference values in a pediatric population. J. Chromatogr. A *617*, 1–10.

142. Lindahl, G., Lindstedt, G., and Lindstedt, S. (1967). Metabolism of 2-amino-5-hydroxyadipic acid in the rat. Arch. Biochem. Biophys. *119*, 347–352.

143. Kaufman, E.E., Nelson, T., Fales, H.M., and Levin, D.M. (1988). Isolation and characterization of a hydroxyacid-oxoacid transhydrogenase from rat kidney mitochondria. J. Biol. Chem. *263*, 16872–16879.

144. Struys, E.A., Verhoeven, N.M., Ten Brink, H.J., Wickenhagen, W.V., Gibson, K.M., and Jakobs, C. (2005). Kinetic characterization of human hydroxyacid-oxoacid transhydrogenase: relevance to D-2-hydroxyglutaric and γ-hydroxybutyric acidurias. J. Inherit. Metab. Dis. *28*, 921–930.

145. Rzem, R., Vincent, M.-F., Van Schaftingen, E., and Veiga-da-Cunha, M. (2007). L-2-hydroxyglutaric aciduria, a defect of metabolite repair. J. Inherit. Metab. Dis. *30*, 681–689.

146. Fan, J., Teng, X., Liu, L., Mattaini, K.R., Looper, R.E., Vander Heiden, M.G., and Rabinowitz, J.D. (2015). Human phosphoglycerate dehydrogenase produces the oncometabolite D-2-hydroxyglutarate. ACS Chem. Biol. *10*, 510–516.

147. Intlekofer, A.M., Dematteo, R.G., Venneti, S., Finley, L.W., Lu, C., Judkins, A.R., Rustenburg, A.S., Grinaway, P.B., Chodera, J.D., Cross, J.R., and Thompson, C.B. (2015). Hypoxia Induces Production of L-2-Hydroxyglutarate. Cell Metab. *22*, 304–311.

148. Oldham, W.M., Clish, C.B., Yang, Y., and Loscalzo, J. (2015). Hypoxia-Mediated Increases in L-2-hydroxyglutarate Coordinate the Metabolic Response to Reductive Stress. Cell Metab. *22*, 291–303.

149. Intlekofer, A.M., Wang, B., Liu, H., Shah, H., Carmona-Fontaine, C., Rustenburg, A.S., Salah, S., Gunner, M.R., Chodera, J.D., Cross, J.R., and Thompson, C.B. (2017). L-2-Hydroxyglutarate production arises from noncanonical enzyme function at acidic pH. Nat. Chem. Biol. *13*, 494–500.

150. Nadtochiy, S.M., Schafer, X., Fu, D., Nehrke, K., Munger, J., and Brookes, P.S. (2016). Acidic pH Is a Metabolic Switch for 2-Hydroxyglutarate Generation and Signaling. J. Biol. Chem. *291*, 20188–20197.

151. Kranendijk, M., Struys, E.A., van Schaftingen, E., Gibson, K.M., Kanhai, W.A., van der Knaap, M.S., Amiel, J., Buist, N.R., Das, A.M., de Klerk, J.B., et al. (2010). *IDH2* mutations in patients with *D*-2-hydroxyglutaric aciduria. Science *330*, 336.

152. Achouri, Y., Noël, G., Vertommen, D., Rider, M.H., Veiga-Da-Cunha, M., and Van Schaftingen, E. (2004). Identification of a dehydrogenase acting on D-2-hydroxyglutarate. Biochem. J. *381*, 35–42.

153. Rzem, R., Veiga-da-Cunha, M., Noël, G., Goffette, S., Nassogne, M.-C., Tabarki, B., Schöller, C., Marquardt, T., Vikkula, M., and Van Schaftingen, E. (2004). A gene encoding a putative FAD-dependent L-2-hydroxyglutarate dehydrogenase is mutated in L-2-hydroxyglutaric aciduria. Proc. Natl. Acad. Sci. USA *101*, 16849–16854.

154. Steenweg, M.E., Jakobs, C., Errami, A., van Dooren, S.J.M., Adeva Bartolomé, M.T., Aerssens, P., Augoustides-Savvapoulou, P., Baric, I., Baumann, M., Bonafé, L., et al. (2010). An overview of L-2-hydroxyglutarate dehydrogenase gene (L2HGDH) variants: a genotype-phenotype study. Hum. Mutat. *31*, 380–390.

155. Struys, E.A., Salomons, G.S., Achouri, Y., Van Schaftingen, E., Grosso, S., Craigen, W.J., Verhoeven, N.M., and Jakobs, C. (2005). Mutations in the D-2-hydroxyglutarate dehydrogenase gene cause D-2-hydroxyglutaric aciduria. Am. J. Hum. Genet. *76*, 358–360.

156. Topçu, M., Jobard, F., Halliez, S., Coskun, T., Yalçinkayal, C., Gerceker, F.O., Wanders, R.J.A., Prud'homme, J.-F., Lathrop, M., Özguc, M., and Fischer, J. (2004). L-2-Hydroxyglutaric aciduria: identification of a mutant gene C14orf160, localized on chromosome 14q22.1. Hum. Mol. Genet. *13*, 2803–2811.

157. Kranendijk, M., Struys, E.A., Gibson, K.M., Wickenhagen, W.V., Abdenur, J.E., Buechner, J., Christensen, E., de Kremer, R.D., Errami, A., Gissen, P., et al. (2010). Evidence for genetic heterogeneity in D-2-hydroxyglutaric aciduria. Hum. Mutat. *31*, 279–283.

158. Kranendijk, M., Struys, E.A., Salomons, G.S., Van der Knaap, M.S., and Jakobs, C. (2012). Progress in understanding 2-hydroxyglutaric acidurias. J. Inherit. Metab. Dis. *35*, 571–587.

CellPress
OPEN ACCESS

159. London, F., and Jeanjean, A. (2015). Gliomatosis cerebri in L-2-hydroxyglutaric aciduria. Acta Neurol. Belg. *115*, 749–751.

160. Patay, Z., Mills, J.C., Löbel, U., Lambert, A., Sablauer, A., and Ellison, D.W. (2012). Cerebral neoplasms in L-2 hydroxyglutaric aciduria: 3 new cases and meta-analysis of literature data. AJNR Am. J. Neuroradiol. *33*, 940–943.

161. Fourati, H., Ellouze, E., Ahmadi, M., Chaari, D., Kamoun, F., Hsairi, I., Triki, C., and Mnif, Z. (2016). MRI features in 17 patients with l2 hydroxyglutaric aciduria. Eur. J. Radiol. Open *3*, 245–250.

162. Pietrak, B., Zhao, H., Qi, H., Quinn, C., Gao, E., Boyer, J.G., Concha, N., Brown, K., Duraiswami, C., Wooster, R., et al. (2011). A tale of two subunits: how the neomorphic R132H IDH1 mutation enhances production of αHG. Biochemistry *50*, 4804–4812.

163. Liu, S., Abboud, M.I., John, T., Mikhailov, V., Hvinden, I., Walsby-Tickle, J., Liu, X., Pettinati, I., Cadoux-Hudson, T., McCullagh, J.S.O., and Schofield, C.J. (2021). Roles of metal ions in the selective inhibition of oncogenic variants of isocitrate dehydrogenase 1. Commun. Biol. *4*, 1243.

164. Deng, G., Shen, J., Yin, M., McManus, J., Mathieu, M., Gee, P., He, T., Shi, C., Bedel, O., McLean, L.R., et al. (2015). Selective inhibition of mutant isocitrate dehydrogenase 1 (IDH1) via disruption of a metal binding network by an allosteric small molecule. J. Biol. Chem. *290*, 762–774.

165. Jin, G., Reitman, Z.J., Spasojevic, I., Batinic-Haberle, I., Yang, J., Schmidt-Kittler, O., Bigner, D.D., and Yan, H. (2011). 2-hydroxyglutarate production, but not dominant negative function, is conferred by glioma-derived NADP-dependent isocitrate dehydrogenase mutations. PLoS ONE *6*, e16812.

166. Ward, P.S., Lu, C., Cross, J.R., Abdel-Wahab, O., Levine, R.L., Schwartz, G.K., and Thompson, C.B. (2013). The potential for isocitrate dehydrogenase mutations to produce 2-hydroxyglutarate depends on allele specificity and subcellular compartmentalization. J. Biol. Chem. *288*, 3804–3815.

167. Moure, C.J., Diplas, B.H., Chen, L.H., Yang, R., Pirozzi, C.J., Wang, Z., Spasojevic, I., Waitkus, M.S., He, Y., and Yan, H. (2019). CRISPR Editing of Mutant IDH1 R132H Induces a CpG Methylation-Low State in Patient-Derived Glioma Models of G-CIMP. Mol. Cancer Res. *17*, 2042–2050.

168. Dexter, J.P., Ward, P.S., Dasgupta, T., Hosios, A.M., Gunawardena, J., and Vander Heiden, M.G. (2018). Lack of evidence for substrate channeling or flux between wildtype and mutant isocitrate dehydrogenase to produce the oncometabolite 2-hydroxyglutarate. J. Biol. Chem. *293*, 20051–20061.

169. Pusch, S., Schweizer, L., Beck, A.-C., Lehmler, J.-M., Weissert, S., Balss, J., Miller, A.K., and von Deimling, A. (2014). D-2-Hydroxyglutarate producing neo-enzymatic activity inversely correlates with frequency of the type of isocitrate dehydrogenase 1 mutations found in glioma. Acta Neuropathol. Commun. *2*, 19.

170. Sahm, F., Capper, D., Pusch, S., Balss, J., Koch, A., Langhans, C.-D., Okun, J.G., and von Deimling, A. (2012). Detection of 2-hydroxyglutarate in formalin-fixed paraffin-embedded glioma specimens by gas chromatography/mass spectrometry. Brain Pathol. *22*, 26–31.

171. Shen, X., Voets, N.L., Larkin, S.J., de Pennington, N., Plaha, P., Stacey, R., McCullagh, J.S.O., Schofield, C.J., Clare, S., Jezzard, P., et al. (2019). A Noninvasive Comparison Study between Human Gliomas with IDH1 and IDH2 Mutations by MR Spectroscopy. Metabolites *9*, 11.

172. Piaskowski, S., Bienkowski, M., Stoczynska-Fidelus, E., Stawski, R., Sieruta, M., Szybka, M., Papierz, W., Wolanczyk, M., Jaskolski, D.J., Liberski, P.P., and Rieske, P. (2011). Glioma cells showing IDH1 mutation cannot be propagated in standard cell culture conditions. Br. J. Cancer *104*, 968–970.

173. Garrett, M., Sperry, J., Braas, D., Yan, W., Le, T.M., Mottahedeh, J., Ludwig, K., Eskin, A., Qin, Y., Levy, R., et al. (2018). Metabolic characterization of isocitrate dehydrogenase (IDH) mutant and IDH wildtype gliomaspheres uncovers cell type-specific vulnerabilities. Cancer Metab. *6*, 4.

174. Luchman, H.A., Stechishin, O.D., Dang, N.H., Blough, M.D., Chesnelong, C., Kelly, J.J., Nguyen, S.A., Chan, J.A., Weljie, A.M., Cairncross, J.G., and Weiss, S. (2012). An in vivo patient-derived model of endogenous IDH1-mutant glioma. Neuro-oncol. *14*, 184–191.

175. Luchman, H.A., Chesnelong, C., Cairncross, J.G., and Weiss, S. (2013). Spontaneous loss of heterozygosity leading to homozygous R132H in a patient-derived IDH1 mutant cell line. Neuro-oncol. *15*, 979–980.

176. Cano-Galiano, A., Oudin, A., Fack, F., Allega, M.-F., Sumpton, D., Martinez-Garcia, E., Dittmar, G., Hau, A.-C., De Falco, A., Herold-Mende, C., et al. (2021). Cystathionine-γ-lyase drives antioxidant defense in cysteine-restricted IDH1-mutant astrocytomas. Neurooncol. Adv. *3*, b057.

177. Izquierdo-Garcia, J.L., Cai, L.M., Chaumeil, M.M., Eriksson, P., Robinson, A.E., Pieper, R.O., Phillips, J.J., and Ronen, S.M. (2014). Glioma cells with the IDH1 mutation modulate metabolic fractional flux through pyruvate carboxylase. PLoS ONE *9*, e108289.

178. Zhang, J., Wang, G., Mao, Q., Li, S., Xiong, W., Lin, Y., and Ge, J. (2016). Glutamate dehydrogenase (GDH) regulates bioenergetics and redox homeostasis in human glioma. Oncotarget *295*, 799–800.

179. Viswanath, P., Radoul, M., Izquierdo-Garcia, J.L., Ong, W.Q., Luchman, H.A., Cairncross, J.G., Huang, B., Pieper, R.O., Phillips, J.J., and Ronen, S.M. (2018). 2-Hydroxyglutarate-Mediated Autophagy of the Endoplasmic Reticulum Leads to an Unusual Downregulation of Phospholipid Biosynthesis in Mutant IDH1 Gliomas. Cancer Res. *78*, 2290–2304.

180. Eckel-Passow, J.E., Lachance, D.H., Molinaro, A.M., Walsh, K.M., Decker, P.A., Sicotte, H., Pekmezci, M., Rice, T., Kosel, M.L., Smirnov, I.V., et al. (2015). Glioma Groups Based on 1p/19q, IDH, and TERT Promoter Mutations in Tumors. N. Engl. J. Med. *372*, 2499–2508.

181. Lenting, K., Verhaak, R., Ter Laan, M., Wesseling, P., and Leenders, W. (2017). Glioma: experimental models and reality. Acta Neuropathol. *133*, 263–282.

182. Carbonneau, M., M Gagné, L., Lalonde, M.E., Germain, M.A., Motorina, A., Guiot, M.C., Secco, B., Vincent, E.E., Tumber, A., Hulea, L., et al. (2016). The oncometabolite 2-hydroxyglutarate activates the mTOR signalling pathway. Nat. Commun. *7*, 12700.

183. Verheul, C., Ntafoulis, I., Kers, T.V., Hoogstrate, Y., Mastroberardino, P.G., Barnhoorn, S., Payán-Gómez, C., Tching Chi Yen, R., Struys, E.A., Koolen, S.L.W., et al. (2021). Generation, characterization, and drug sensitivities of 12 patient-derived IDH1-mutant glioma cell cultures. Neurooncol. Adv. *3*, vdab103.

184. Kelly, J.J.P., Blough, M.D., Stechishin, O.D.M., Chan, J.A.W., Beauchamp, D., Perizzolo, M., Demetrick, D.J., Steele, L., Auer, R.N., Hader, W.J., et al. (2010). Oligodendroglioma cell lines containing t(1;19)(q10;p10). Neuro-oncol. *12*, 745–755.

185. Dao Trong, P., Rösch, S., Mairbäurl, H., Pusch, S., Unterberg, A., Herold-Mende, C., and Warta, R. (2018). Identification of a Prognostic Hypoxia-Associated Gene Set in IDH-Mutant Glioma. Int. J. Mol. Sci. *19*, 2903.

186. Trong, P.D., Jungwirth, G., Yu, T., Pusch, S., Unterberg, A., Herold-Mende, C., and Warta, R. (2020). Large-Scale Drug Screening in Patient-Derived IDH(mut)Glioma Stem Cells Identifies Several Efficient Drugs among FDA-Approved Antineoplastic Agents. Cells *9*, 1389.

187. Klink, B., Miletic, H., Stieber, D., Huszthy, P.C., Campos Valenzuela, J.A., Balss, J., Wang, J., Schubert, M., Sakariassen, P.Ø., Sundstrøm, T., et al. (2013). A novel, diffusely infiltrative xenograft model of human anaplastic oligodendroglioma with mutations in FUBP1, CIC, and IDH1. PLoS ONE *8*, e59773.

188. Navis, A.C., Niclou, S.P., Fack, F., Stieber, D., van Lith, S., Verrijp, K., Wright, A., Stauber, J., Tops, B., Otte-Holler, I., et al. (2013). Increased mitochondrial activity in a novel IDH1-R132H mutant human oligodendroglioma xenograft model: in situ detection of 2-HG and α-KG. Acta Neuropathol. Commun. *1*, 18.

189. Golebiewska, A., Hau, A.-C., Oudin, A., Stieber, D., Yabo, Y.A., Baus, V., Barthelemy, V., Klein, E., Bougnaud, S., Keunen, O., et al. (2020). Patient-

derived organoids and orthotopic xenografts of primary and recurrent gliomas represent relevant patient avatars for precision oncology. Acta Neuropathol. *140*, 919–949.

190. Addie, R.D., de Jong, Y., Alberti, G., Kruisselbrink, A.B., Que, I., Baelde, H., and Bovée, J.V.M.G. (2019). Exploration of the chondrosarcoma metabolome; the mTOR pathway as an important pro-survival pathway. J. Bone Oncol. *15*, 100222.

191. Ma, S., Jiang, B., Deng, W., Gu, Z.-K., Wu, F.-Z., Li, T., Xia, Y., Yang, H., Ye, D., Xiong, Y., and Guan, K.L. (2015). D-2-hydroxyglutarate is essential for maintaining oncogenic property of mutant IDH-containing cancer cells but dispensable for cell growth. Oncotarget *6*, 8606–8620.

192. Peterse, E.F.P., Niessen, B., Addie, R.D., de Jong, Y., Cleven, A.H.G., Kruisselbrink, A.B., van den Akker, B.E.W.M., Molenaar, R.J., Cleton-Jansen, A.-M., and Bovée, J.V.M.G. (2018). Targeting glutaminolysis in chondrosarcoma in context of the IDH1/2 mutation. Br. J. Cancer *118*, 1074–1083.

193. Peterse, E.F.P., van den Akker, B.E.W.M., Niessen, B., Oosting, J., Suijker, J., de Jong, Y., Danen, E.H.J., Cleton-Jansen, A.-M., and Bovée, J.V.M.G. (2017). NAD Synthesis Pathway Interference Is a Viable Therapeutic Strategy for Chondrosarcoma. Mol. Cancer Res. *15*, 1714–1721.

194. Salamanca-Cardona, L., Shah, H., Poot, A.J., Correa, F.M., Di Gialleonardo, V., Lui, H., Miloushev, V.Z., Granlund, K.L., Tee, S.S., Cross, J.R., et al. (2017). In Vivo Imaging of Glutamine Metabolism to the Oncometabolite 2-Hydroxyglutarate in IDH1/2 Mutant Tumors. Cell Metab. *26*, 830–841.e3.

195. van Oosterwijk, J.G., de Jong, D., van Ruler, M.A.J.H., Hogendoorn, P.C.W., Dijkstra, P.D.S., van Rijswijk, C.S.P., Machado, I., Llombart-Bosch, A., Szuhai, K., and Bovée, J.V.M.G. (2012). Three new chondrosarcoma cell lines: one grade III conventional central chondrosarcoma and two dedifferentiated chondrosarcomas of bone. BMC Cancer *12*, 375.

196. Carrabba, M.G., Tavel, L., Oliveira, G., Forcina, A., Quilici, G., Nardelli, F., Tresoldi, C., Ambrosi, A., Ciceri, F., Bernardi, M., et al. (2016). Integrating a prospective pilot trial and patient-derived xenografts to trace metabolic changes associated with acute myeloid leukemia. J. Hematol. Oncol. *9*, 115.

197. Boutzen, H., Saland, E., Larrue, C., de Toni, F., Gales, L., Castelli, F.A., Cathebas, M., Zaghdoudi, S., Stuani, L., Kaoma, T., et al. (2016). Isocitrate dehydrogenase 1 mutations prime the all-trans retinoic acid myeloid differentiation pathway in acute myeloid leukemia. J. Exp. Med. *213*, 483–497.

198. Emadi, A., Jun, S.A., Tsukamoto, T., Fathi, A.T., Minden, M.D., and Dang, C.V. (2014). Inhibition of glutaminase selectively suppresses the growth of primary acute myeloid leukemia cells with IDH mutations. Exp. Hematol. *42*, 247–251.

199. Fujiwara, H., Tateishi, K., Misumi, K., Hayashi, A., Igarashi, K., Kato, H., Nakatsuka, T., Suzuki, N., Yamamoto, K., Kudo, Y., et al. (2019). Mutant IDH1 confers resistance to energy stress in normal biliary cells through PFKP-induced aerobic glycolysis and AMPK activation. Sci. Rep. *9*, 18859.

200. Saha, S.K., Parachoniak, C.A., Ghanta, K.S., Fitamant, J., Ross, K.N., Najem, M.S., Gurumurthy, S., Akbay, E.A., Sia, D., Cornella, H., et al. (2014). Mutant IDH inhibits HNF-4α to block hepatocyte differentiation and promote biliary cancer. Nature *513*, 110–114.

201. McBrayer, S.K., Mayers, J.R., DiNatale, G.J., Shi, D.D., Khanal, J., Chakraborty, A.A., Sarosiek, K.A., Briggs, K.J., Robbins, A.K., Sewastianik, T., et al. (2018). Transaminase Inhibition by 2-Hydroxyglutarate Impairs Glutamate Biosynthesis and Redox Homeostasis in Glioma. Cell *175*, 101–116.e25.

202. Nakagawa, M., Nakatani, F., Matsunaga, H., Seki, T., Endo, M., Ogawara, Y., Machida, Y., Katsumoto, T., Yamagata, K., Hattori, A., et al. (2019). Selective inhibition of mutant IDH1 by DS-1001b ameliorates aberrant histone modifications and impairs tumor activity in chondrosarcoma. Oncogene *38*, 6835–6849.

203. Suijker, J., Oosting, J., Koornneef, A., Struys, E.A., Salomons, G.S., Schaap, F.G., Waaijer, C.J.F., Wijers-Koster, P.M., Briaire-de Bruijn, I.H., Haazen, L., et al. (2015). Inhibition of mutant IDH1 decreases D-2-HG levels without affecting tumorigenic properties of chondrosarcoma cell lines. Oncotarget *6*, 12505–12519.

204. Lo Presti, C., Fauvelle, F., Jacob, M.C., Mondet, J., and Mossuz, P. (2021). The metabolic reprogramming in acute myeloid leukemia patients depends on their genotype and is a prognostic marker. Blood Adv. *5*, 156–166.

205. Mohammad, N., Wong, D., Lum, A., Lin, J., Ho, J., Lee, C.-H., and Yip, S. (2020). Characterisation of isocitrate dehydrogenase 1/isocitrate dehydrogenase 2 gene mutation and the d-2-hydroxyglutarate oncometabolite level in dedifferentiated chondrosarcoma. Histopathology *76*, 722–730.

206. Winter, H., Kaisaki, P.J., Harvey, J., Giacopuzzi, E., Ferla, M.P., Pentony, M.M., Knight, S.J.L., Sharma, R.A., Taylor, J.C., and McCullagh, J.S.O. (2019). Identification of Circulating Genomic and Metabolic Biomarkers in Intrahepatic Cholangiocarcinoma. Cancers (Basel) *11*, 1895.

207. Grassian, A.R., Parker, S.J., Davidson, S.M., Divakaruni, A.S., Green, C.R., Zhang, X., Slocum, K.L., Pu, M., Lin, F., Vickers, C., et al. (2014). IDH1 mutations alter citric acid cycle metabolism and increase dependence on oxidative mitochondrial metabolism. Cancer Res. *74*, 3317–3331.

208. Li, S., Chou, A.P., Chen, W., Chen, R., Deng, Y., Phillips, H.S., Selfridge, J., Zurayk, M., Lou, J.J., Everson, R.G., et al. (2013). Overexpression of isocitrate dehydrogenase mutant proteins renders glioma cells more sensitive to radiation. Neuro-oncol. *15*, 57–68.

209. Su, L., Zhang, X., Zheng, L., Wang, M., Zhu, Z., and Li, P. (2020). Mutation of *Isocitrate Dehydrogenase 1* in Cholangiocarcinoma Impairs Tumor Progression by Inhibiting Isocitrate Metabolism. Front. Endocrinol. (Lausanne) *11*, 189.

210. Wei, S., Wang, J., Oyinlade, O., Ma, D., Wang, S., Kratz, L., Lal, B., Xu, Q., Liu, S., Shah, S.R., et al. (2018). Heterozygous IDH1$^{R132H/WT}$ created by "single base editing" inhibits human astroglial cell growth by downregulating YAP. Oncogene *37*, 5160–5174.

211. Su, R., Dong, L., Li, C., Nachtergaele, S., Wunderlich, M., Qing, Y., Deng, X., Wang, Y., Weng, X., Hu, C., et al. (2018). R-2HG Exhibits Anti-tumor Activity by Targeting FTO/m^{6}A/MYC/CEBPA Signaling. Cell *172*, 90–105.e23.

212. Khurshed, M., Molenaar, R.J., Lenting, K., Leenders, W.P., and van Noorden, C.J.F. (2017). In silico gene expression analysis reveals glycolysis and acetate anaplerosis in IDH1 wild-type glioma and lactate and glutamate anaplerosis in IDH1-mutated glioma. Oncotarget *8*, 49165–49177.

213. Dekker, L.J.M., Wu, S., Jurriëns, C., Mustafa, D.A.N., Grevers, F., Burgers, P.C., Sillevis Smitt, P.A.E., Kros, J.M., and Luider, T.M. (2020). Metabolic changes related to the IDH1 mutation in gliomas preserve TCA-cycle activity: An investigation at the protein level. FASEB J. *34*, 3646–3657.

214. Tanaka, K., Sasayama, T., Irino, Y., Takata, K., Nagashima, H., Satoh, N., Kyotani, K., Mizowaki, T., Imahori, T., Ejima, Y., et al. (2015). Compensatory glutamine metabolism promotes glioblastoma resistance to mTOR inhibitor treatment. J. Clin. Invest. *125*, 1591–1602.

215. Mashimo, T., Pichumani, K., Vemireddy, V., Hatanpaa, K.J., Singh, D.K., Sirasanagandla, S., Nannepaga, S., Piccirillo, S.G., Kovacs, Z., Foong, C., et al. (2014). Acetate is a bioenergetic substrate for human glioblastoma and brain metastases. Cell *159*, 1603–1614.

216. Viswanath, P., Najac, C., Izquierdo-Garcia, J.L., Pankov, A., Hong, C., Eriksson, P., Costello, J.F., Pieper, R.O., and Ronen, S.M. (2016). Mutant IDH1 expression is associated with down-regulation of monocarboxylate transporters. Oncotarget *7*, 34942–34955.

217. Chaumeil, M.M., Radoul, M., Najac, C., Eriksson, P., Viswanath, P., Blough, M.D., Chesnelong, C., Luchman, H.A., Cairncross, J.G., and Ronen, S.M. (2016). Hyperpolarized (13)C MR imaging detects no lactate

production in mutant IDH1 gliomas: Implications for diagnosis and response monitoring. Neuroimage Clin. *12*, 180–189.

218. Chesnelong, C., Chaumeil, M.M., Blough, M.D., Al-Najjar, M., Stechishin, O.D., Chan, J.A., Pieper, R.O., Ronen, S.M., Weiss, S., Luchman, H.A., and Cairncross, J.G. (2014). Lactate dehydrogenase A silencing in IDH mutant gliomas. Neuro-oncol. *16*, 686–695.

219. Izquierdo-Garcia, J.L., Viswanath, P., Eriksson, P., Cai, L., Radoul, M., Chaumeil, M.M., Blough, M., Luchman, H.A., Weiss, S., Cairncross, J.G., et al. (2015). IDH1 Mutation Induces Reprogramming of Pyruvate Metabolism. Cancer Res. *75*, 2999–3009.

220. Turcan, S., Rohle, D., Goenka, A., Walsh, L.A., Fang, F., Yilmaz, E., Campos, C., Fabius, A.W.M., Lu, C., Ward, P.S., et al. (2012). IDH1 mutation is sufficient to establish the glioma hypermethylator phenotype. Nature *483*, 479–483.

221. Waitkus, M.S., Pirozzi, C.J., Moure, C.J., Diplas, B.H., Hansen, L.J., Carpenter, A.B., Yang, R., Wang, Z., Ingram, B.O., Karoly, E.D., et al. (2018). Adaptive Evolution of the GDH2 Allosteric Domain Promotes Gliomagenesis by Resolving IDH1^{R132H}-Induced Metabolic Liabilities. Cancer Res. *78*, 36–50.

222. Chen, R., Nishimura, M.C., Kharbanda, S., Peale, F., Deng, Y., Daemen, A., Forrest, W.F., Kwong, M., Hedehus, M., Hatzivassiliou, G., et al. (2014). Hominoid-specific enzyme GLUD2 promotes growth of IDH1^{R132H} glioma. Proc. Natl. Acad. Sci. USA *111*, 14217–14222.

223. García-Espinosa, M.A., Wallin, R., Hutson, S.M., and Sweatt, A.J. (2007). Widespread neuronal expression of branched-chain aminotransferase in the CNS: implications for leucine/glutamate metabolism and for signaling by amino acids. J. Neurochem. *100*, 1458–1468.

224. Tönjes, M., Barbus, S., Park, Y.J., Wang, W., Schlotter, M., Lindroth, A.M., Pleier, S.V., Bai, A.H.C., Karra, D., Piro, R.M., et al. (2013). BCAT1 promotes cell proliferation through amino acid catabolism in gliomas carrying wild-type IDH1. Nat. Med. *19*, 901–908.

225. Ichihara, A., and Koyama, E. (1966). Transaminase of branched chain amino acids. I. Branched chain amino acids-alpha-ketoglutarate transaminase. J. Biochem. *59*, 160–169.

226. Yang, L., Venneti, S., and Nagrath, D. (2017). Glutaminolysis: A Hallmark of Cancer Metabolism. Annu. Rev. Biomed. Eng. *19*, 163–194.

227. Seltzer, M.J., Bennett, B.D., Joshi, A.D., Gao, P., Thomas, A.G., Ferraris, D.V., Tsukamoto, T., Rojas, C.J., Slusher, B.S., Rabinowitz, J.D., et al. (2010). Inhibition of glutaminase preferentially slows growth of glioma cells with mutant IDH1. Cancer Res. *70*, 8981–8987.

228. Muir, A., Danai, L.V., Gui, D.Y., Waingarten, C.Y., Lewis, C.A., and Vander Heiden, M.G. (2017). Environmental cystine drives glutamine anaplerosis and sensitizes cancer cells to glutaminase inhibition. eLife *6*, e27713.

229. Karlstaedt, A., Zhang, X., Vitrac, H., Harmancey, R., Vasquez, H., Wang, J.H., Goodell, M.A., and Taegtmeyer, H. (2016). Oncometabolite d-2-hydroxyglutarate impairs α-ketoglutarate dehydrogenase and contractile function in rodent heart. Proc. Natl. Acad. Sci. USA *113*, 10436–10441.

230. Moloney, J.N., and Cotter, T.G. (2018). ROS signalling in the biology of cancer. Semin. Cell Dev. Biol. *80*, 50–64.

231. Griffith, O.W. (1999). Biologic and pharmacologic regulation of mammalian glutathione synthesis. Free Radic. Biol. Med. *27*, 922–935.

232. Chen, L., Zhang, Z., Hoshino, A., Zheng, H.D., Morley, M., Arany, Z., and Rabinowitz, J.D. (2019). NADPH production by the oxidative pentose-phosphate pathway supports folate metabolism. Nat. Metab. *1*, 404–415.

233. Charitou, P., Rodriguez-Colman, M., Gerrits, J., van Triest, M., Groot Koerkamp, M., Hornsveld, M., Holstege, F., Verhoeven-Duif, N.M., and Burgering, B.M.T. (2015). FOXOs support the metabolic requirements of normal and tumor cells by promoting IDH1 expression. EMBO Rep. *16*, 456–466.

234. Han, S.J., Choi, H.S., Kim, J.I., Park, J.-W., and Park, K.M. (2018). IDH2 deficiency increases the liver susceptibility to ischemia-reperfusion injury via increased mitochondrial oxidative injury. Redox Biol. *14*, 142–153.

235. Han, S.J., Jang, H.-S., Noh, M.R., Kim, J., Kong, M.J., Kim, J.I., Park, J.-W., and Park, K.M. (2017). Mitochondrial NADP^{+}-Dependent Isocitrate Dehydrogenase Deficiency Exacerbates Mitochondrial and Cell Damage after Kidney Ischemia-Reperfusion Injury. J. Am. Soc. Nephrol. *28*, 1200–1215.

236. Ku, H.J., Ahn, Y., Lee, J.H., Park, K.M., and Park, J.-W. (2015). IDH2 deficiency promotes mitochondrial dysfunction and cardiac hypertrophy in mice. Free Radic. Biol. Med. *80*, 84–92.

237. Park, J.H., Ku, H.J., Lee, J.H., and Park, J.-W. (2017). *Idh2* Deficiency Exacerbates Acrolein-Induced Lung Injury through Mitochondrial Redox Environment Deterioration. Oxid. Med. Cell. Longev. *2017*, 1595103.

238. Calvert, A.E., Chalastanis, A., Wu, Y., Hurley, L.A., Kouri, F.M., Bi, Y., Kachman, M., May, J.L., Bartom, E., Hua, Y., et al. (2017). Cancer-Associated IDH1 Promotes Growth and Resistance to Targeted Therapies in the Absence of Mutation. Cell Rep. *19*, 1858–1873.

239. Wahl, D.R., Dresser, J., Wilder-Romans, K., Parsels, J.D., Zhao, S.G., Davis, M., Zhao, L., Kachman, M., Wernisch, S., Burant, C.F., et al. (2017). Glioblastoma Therapy Can Be Augmented by Targeting IDH1-Mediated NADPH Biosynthesis. Cancer Res. *77*, 960–970.

240. DiNardo, C.D., Jabbour, E., Ravandi, F., Takahashi, K., Daver, N., Routbort, M., Patel, K.P., Brandt, M., Pierce, S., Kantarjian, H., and Garcia-Manero, G. (2016). IDH1 and IDH2 mutations in myelodysplastic syndromes and role in disease progression. Leukemia *30*, 980–984.

241. Goyal, L., Govindan, A., Sheth, R.A., Nardi, V., Blaszkowsky, L.S., Faris, J.E., Clark, J.W., Ryan, D.P., Kwak, E.L., Allen, J.N., et al. (2015). Prognosis and Clinicopathologic Features of Patients With Advanced Stage Isocitrate Dehydrogenase (IDH) Mutant and IDH Wild-Type Intrahepatic Cholangiocarcinoma. Oncologist *20*, 1019–1027.

242. Petersen, J.K., Boldt, H.B., Sørensen, M.D., Blach, S., Dahlrot, R.H., Hansen, S., Burton, M., Thomassen, M., Kruse, T., Poulsen, F.R., et al. (2021). Targeted next-generation sequencing of adult gliomas for retrospective prognostic evaluation and up-front diagnostics. Neuropathol. Appl. Neurobiol. *47*, 108–126.

243. de Jong, Y., Ingola, M., Briaire-de Bruijn, I.H., Kruisselbrink, A.B., Venneker, S., Palubeckaite, I., Heijs, B.P.A.M., Cleton-Jansen, A.-M., Haas, R.L.M., and Bovée, J.V.M.G. (2019). Radiotherapy resistance in chondrosarcoma cells; a possible correlation with alterations in cell cycle related genes. Clin. Sarcoma Res. *9*, 9.

244. Leonardi, R., Subramanian, C., Jackowski, S., and Rock, C.O. (2012). Cancer-associated isocitrate dehydrogenase mutations inactivate NADPH-dependent reductive carboxylation. J. Biol. Chem. *287*, 14615–14620.

245. Wenger, K.J., Hattingen, E., Franz, K., Steinbach, J., Bähr, O., and Pilatus, U. (2019). In vivo Metabolic Profiles as Determined by ^{31}P and short TE ^{1}H MR-Spectroscopy : No Difference Between Patients with IDH Wildtype and IDH Mutant Gliomas. Clin. Neuroradiol. *29*, 27–36.

246. Abou-Alfa, G.K., Macarulla, T., Javle, M.M., Kelley, R.K., Lubner, S.J., Adeva, J., Cleary, J.M., Catenacci, D.V., Borad, M.J., Bridgewater, J., et al. (2020). Ivosidenib in IDH1-mutant, chemotherapy-refractory cholangiocarcinoma (ClarIDHy): a multicentre, randomised, double-blind, placebo-controlled, phase 3 study. Lancet Oncol. *21*, 796–807.

247. Stein, E.M., DiNardo, C.D., Fathi, A.T., Pollyea, D.A., Stone, R.M., Altman, J.K., Roboz, G.J., Patel, M.R., Collins, R., Flinn, I.W., et al. (2019). Molecular remission and response patterns in patients with mutant-*IDH2* acute myeloid leukemia treated with enasidenib. Blood *133*, 676–687.

248. Mellinghoff, I.K., Ellingson, B.M., Touat, M., Maher, E., De La Fuente, M.I., Holdhoff, M., Cote, G.M., Burris, H., Janku, F., Young, R.J., et al. (2020). Ivosidenib in Isocitrate Dehydrogenase 1-Mutated Advanced Glioma. J. Clin. Oncol. *38*, 3398–3406.

249. Li, L., Paz, A.C., Wilky, B.A., Johnson, B., Galoian, K., Rosenberg, A., Hu, G., Tinoco, G., Bodamer, O., and Trent, J.C. (2015). Treatment with a Small Molecule Mutant IDH1 Inhibitor Suppresses Tumorigenic Activity

and Decreases Production of the Oncometabolite 2-Hydroxyglutarate in Human Chondrosarcoma Cells. PLoS ONE *10*, e0133813.

250. Chaturvedi, A., Herbst, L., Pusch, S., Klett, L., Goparaju, R., Stichel, D., Kaulfuss, S., Panknin, O., Zimmermann, K., Toschi, L., et al. (2017). Pan-mutant-IDH1 inhibitor BAY1436032 is highly effective against human IDH1 mutant acute myeloid leukemia in vivo. Leukemia *31*, 2020–2028.

251. Rohle, D., Popovici-Muller, J., Palaskas, N., Turcan, S., Grommes, C., Campos, C., Tsoi, J., Clark, O., Oldrini, B., Komisopoulou, E., et al. (2013). An inhibitor of mutant IDH1 delays growth and promotes differentiation of glioma cells. Science *340*, 626–630.

252. Molloy, A.R., Najac, C., Viswanath, P., Lakhani, A., Subramani, E., Batsios, G., Radoul, M., Gillespie, A.M., Pieper, R.O., and Ronen, S.M. (2020). MR-detectable metabolic biomarkers of response to mutant IDH inhibition in low-grade glioma. Theranostics *10*, 8757–8770.

253. Radoul, M., Hong, D., Gillespie, A.M., Najac, C., Viswanath, P., Pieper, R.O., Costello, J.F., Luchman, H.A., and Ronen, S.M. (2021). Early Noninvasive Metabolic Biomarkers of Mutant IDH Inhibition in Glioma. Metabolites *11*, 109.

254. Andronesi, O.C., Arrillaga-Romany, I.C., Ly, K.I., Bogner, W., Ratai, E.M., Reitz, K., Iafrate, A.J., Dietrich, J., Gerstner, E.R., Chi, A.S., et al. (2018). Pharmacodynamics of mutant-IDH1 inhibitors in glioma patients probed by in vivo 3D MRS imaging of 2-hydroxyglutarate. Nat. Commun. *9*, 1474.

255. DiNardo, C.D., Stein, E.M., de Botton, S., Roboz, G.J., Altman, J.K., Mims, A.S., Swords, R., Collins, R.H., Mannis, G.N., Pollyea, D.A., et al. (2018). Durable Remissions with Ivosidenib in IDH1-Mutated Relapsed or Refractory AML. N. Engl. J. Med. *378*, 2386–2398.

256. Roboz, G.J., DiNardo, C.D., Stein, E.M., de Botton, S., Mims, A.S., Prince, G.T., Altman, J.K., Arellano, M.L., Donnellan, W., Erba, H.P., et al. (2020). Ivosidenib induces deep durable remissions in patients with newly diagnosed IDH1-mutant acute myeloid leukemia. Blood *135*, 463–471.

257. Khurshed, M., Aarnoudse, N., Hulsbos, R., Hira, V.V.V., van Laarhoven, H.W.M., Wilmink, J.W., Molenaar, R.J., and van Noorden, C.J.F. (2018). IDH1-mutant cancer cells are sensitive to cisplatin and an IDH1-mutant inhibitor counteracts this sensitivity. FASEB J. *32*, fj201800547R.

258. Molenaar, R.J., Botman, D., Smits, M.A., Hira, V.V., van Lith, S.A., Stap, J., Henneman, P., Khurshed, M., Lenting, K., Mul, A.N., et al. (2015). Radioprotection of *IDH1*-Mutated Cancer Cells by the IDH1-Mutant Inhibitor AGI-5198. Cancer Res. *75*, 4790–4802.

259. Wang, P., Wu, J., Ma, S., Zhang, L., Yao, J., Hoadley, K.A., Wilkerson, M.D., Perou, C.M., Guan, K.-L., Ye, D., and Xiong, Y. (2015). Oncometabolite D-2-Hydroxyglutarate Inhibits ALKBH DNA Repair Enzymes and Sensitizes IDH Mutant Cells to Alkylating Agents. Cell Rep. *13*, 2353–2361.

260. Tocci, A., Parolini, I., Gabbianelli, M., Testa, U., Luchetti, L., Samoggia, P., Masella, B., Russo, G., Valtieri, M., and Peschle, C. (1996). Dual action of retinoic acid on human embryonic/fetal hematopoiesis: blockade of primitive progenitor proliferation and shift from multipotent/erythroid/monocytic to granulocytic differentiation program. Blood *88*, 2878–2888.

261. Kim, Y., Jeung, H.K., Cheong, J.W., Song, J., Bae, S.H., Lee, J.I., and Min, Y.H. (2020). All-Trans Retinoic Acid Synergizes with Enasidenib to Induce Differentiation of IDH2-Mutant Acute Myeloid Leukemia Cells. Yonsei Med. J. *61*, 762–773.

262. Molenaar, R.J., Coelen, R.J.S., Khurshed, M., Roos, E., Caan, M.W.A., van Linde, M.E., Kouwenhoven, M., Bramer, J.A.M., Bovée, J.V.M.G., Mathôt, R.A., et al. (2017). Study protocol of a phase IB/II clinical trial of metformin and chloroquine in patients with *IDH1*-mutated or *IDH2*-mutated solid tumours. BMJ Open *7*, e014961.

263. Elhammali, A., Ippolito, J.E., Collins, L., Crowley, J., Marasa, J., and Piwnica-Worms, D. (2014). A high-throughput fluorimetric assay for 2-hydroxyglutarate identifies Zaprinast as a glutaminase inhibitor. Cancer Discov. *4*, 828–839.

264. Cuyàs, E., Fernández-Arroyo, S., Corominas-Faja, B., Rodríguez-Gallego, E., Bosch-Barrera, J., Martin-Castillo, B., De Llorens, R., Joven, J., and Menendez, J.A.J.O. (2015). Oncometabolic mutation IDH1 R132H confers a metformin-hypersensitive phenotype. Oncotarget *6*, 12279–12296.

265. Harding, J.J., Telli, M., Munster, P., Voss, M.H., Infante, J.R., DeMichele, A., Dunphy, M., Le, M.H., Molineaux, C., Orford, K., et al. (2021). A Phase I Dose-Escalation and Expansion Study of Telaglenastat in Patients with Advanced or Metastatic Solid Tumors. Clin. Cancer Res. *27*, 4994–5003.

266. Saxena, K., Konopleva, M., Bhagat, T.D., Guerra, V.A., Maduike, R., Tiziani, S., Borthakur, G., Jabbour, E., Pemmaraju, N., Kadia, T.M., et al. (2020). AZA + Glutaminase Inhibition with Telaglenastat (CB-839) for Advanced MDS: An Updated Interim Analysis. Blood *136*, 31–32.

267. Tannir, N.M., Agarwal, N., Porta, C., Lawrence, N.J., Motzer, R.J., Lee, R.J., Jain, R.K., Davis, N.B., Appleman, L.J., Goodman, O.B., et al. (2021). CANTATA: Primary analysis of a global, randomized, placebo (Pbo)-controlled, double-blind trial of telaglenastat (CB-839) + cabozantinib versus Pbo + cabozantinib in advanced/metastatic renal cell carcinoma (mRCC) patients (pts) who progressed on immune checkpoint inhibitor (ICI) or anti-angiogenic therapies. J. Clin. Oncol. *39*, 4501.

268. Motzer, R.J., Lee, C.H., Emamekhoo, H., Matrana, M., Percent, I., Hsieh, J.J., Hussain, A., Vaishampayan, U.N., Graham, R., Liu, S., et al. (2019). LBA54 - ENTRATA: Randomized, double-blind, phase II study of telaglenastat (tela; CB-839) + everolimus (E) vs placebo (pbo) + E in patients (pts) with advanced/metastatic renal cell carcinoma (mRCC). Ann. Oncol. *30*, v889–v890.

269. Motzer, R.J., Escudier, B., Oudard, S., Hutson, T.E., Porta, C., Bracarda, S., Grünwald, V., Thompson, J.A., Figlin, R.A., Hollaender, N., et al.; RECORD-1 Study Group (2008). Efficacy of everolimus in advanced renal cell carcinoma: a double-blind, randomised, placebo-controlled phase III trial. Lancet *372*, 449–456.

270. Choueiri, T.K., Escudier, B., Powles, T., Tannir, N.M., Mainwaring, P.N., Rini, B.I., Hammers, H.J., Donskov, F., Roth, B.J., Peltola, K., et al.; METEOR investigators (2016). Cabozantinib versus everolimus in advanced renal cell carcinoma (METEOR): final results from a randomised, open-label, phase 3 trial. Lancet Oncol. *17*, 917–927.

271. Mukhopadhyay, S., Goswami, D., Adiseshaiah, P.P., Burgan, W., Yi, M., Guerin, T.M., Kozlov, S.V., Nissley, D.V., and McCormick, F. (2020). Undermining Glutaminolysis Bolsters Chemotherapy While NRF2 Promotes Chemoresistance in KRAS-Driven Pancreatic Cancers. Cancer Res. *80*, 1630–1643.

272. Romero, R., Sayin, V.I., Davidson, S.M., Bauer, M.R., Singh, S.X., LeBoeuf, S.E., Karakousi, T.R., Ellis, D.C., Bhutkar, A., Sánchez-Rivera, F.J., et al. (2017). Keap1 loss promotes Kras-driven lung cancer and results in dependence on glutaminolysis. Nat. Med. *23*, 1362–1368.

273. Loeb, F., Clark, W.M., Coatney, G.R., Coggeshall, L.T., Dieuaide, F.R., Dochez, A.R., Hakansson, E.G., Marshall, E.K., Jr., Marvel, C.S., McCoy, O.R., et al. (1946). ACTIVITY OF A NEW ANTIMALARIAL AGENT, CHLOROQUINE (SN 7618): Statement Approved by the Board for Coordination of Malarial Studies. J. Am. Med. Assoc. *130*, 1069–1070.

274. Pascolo, S. (2016). Time to use a dose of Chloroquine as an adjuvant to anti-cancer chemotherapies. Eur. J. Pharmacol. *771*, 139–144.

275. Choi, M.-M., Kim, E.-A., Choi, S.-Y., Kim, T.-U., Cho, S.-W., and Yang, S.-J. (2007). Inhibitory properties of nerve-specific human glutamate dehydrogenase isozyme by chloroquine. J. Biochem. Mol. Biol. *40*, 1077–1082.

276. Firat, E., Weyerbrock, A., Gaedicke, S., Grosu, A.-L., and Niedermann, G. (2012). Chloroquine or chloroquine-PI3K/Akt pathway inhibitor combinations strongly promote γ-irradiation-induced cell death in primary stem-like glioma cells. PLoS ONE *7*, e47357.

277. Grønningsæter, I.S., Reikvam, H., Aasebø, E., Bartaula-Brevik, S., Hernandez-Valladares, M., Selheim, F., Berven, F.S., Tvedt, T.H., Bruserud, Ø., and Hatfield, K.J. (2021). Effects of the Autophagy-Inhibiting Agent

Chloroquine on Acute Myeloid Leukemia Cells; Characterization of Patient Heterogeneity. J. Pers. Med. *11*, 779.

278. Sanchez-Rangel, E., and Inzucchi, S.E. (2017). Metformin: clinical use in type 2 diabetes. Diabetologia *60*, 1586–1593.

279. Evans, J.M.M., Donnelly, L.A., Emslie-Smith, A.M., Alessi, D.R., and Morris, A.D. (2005). Metformin and reduced risk of cancer in diabetic patients. BMJ *330*, 1304–1305.

280. Libby, G., Donnelly, L.A., Donnan, P.T., Alessi, D.R., Morris, A.D., and Evans, J.M.M. (2009). New users of metformin are at low risk of incident cancer: a cohort study among people with type 2 diabetes. Diabetes Care *32*, 1620–1625.

281. Zhao, B., Luo, J., Yu, T., Zhou, L., Lv, H., and Shang, P. (2020). Anticancer mechanisms of metformin: A review of the current evidence. Life Sci. *254*, 117717.

282. Zhang, H.-H., and Guo, X.-L. (2016). Combinational strategies of metformin and chemotherapy in cancers. Cancer Chemother. Pharmacol. *78*, 13–26.

283. Yu, Z., Zhao, G., Xie, G., Zhao, L., Chen, Y., Yu, H., Zhang, Z., Li, C., and Li, Y. (2015). Metformin and temozolomide act synergistically to inhibit growth of glioma cells and glioma stem cells in vitro and in vivo. Oncotarget *6*, 32930–32943.

284. Yuan, F., Cheng, C., Xiao, F., Liu, H., Cao, S., and Zhou, G. (2020). Inhibition of mTORC1/P70S6K pathway by Metformin synergistically sensitizes Acute Myeloid Leukemia to Ara-C. Life Sci. *243*, 117276.

285. Singh-Makkar, S., Pandav, K., Hathaway, D., Paul, T., and Youssef, P. (2021). Multidimensional mechanisms of metformin in cancer treatment. Tumori, 03008916211023548.

286. Khurshed, M., Molenaar, R.J., van Linde, M.E., Mathôt, R.A., Struys, E.A., van Wezel, T., van Noorden, C.J.F., Klümpen, H.-J., Bovée, J.V.M.G., and Wilmink, J.W. (2021). A Phase Ib Clinical Trial of Metformin and Chloroquine in Patients with *IDH1*-Mutated Solid Tumors. Cancers (Basel) *13*, 2474.

287. Phannasil, P., Thuwajit, C., Warnnissorn, M., Wallace, J.C., MacDonald, M.J., and Jitrapakdee, S. (2015). Pyruvate Carboxylase Is Up-Regulated in Breast Cancer and Essential to Support Growth and Invasion of MDA-MB-231 Cells. PLoS ONE *10*, e0129848.

288. Sellers, K., Fox, M.P., Bousamra, M., 2nd, Slone, S.P., Higashi, R.M., Miller, D.M., Wang, Y., Yan, J., Yuneva, M.O., Deshpande, R., et al. (2015). Pyruvate carboxylase is critical for non-small-cell lung cancer proliferation. J. Clin. Invest. *125*, 687–698.

289. Cheng, T., Sudderth, J., Yang, C., Mullen, A.R., Jin, E.S., Matés, J.M., and DeBerardinis, R.J. (2011). Pyruvate carboxylase is required for glutamine-independent growth of tumor cells. Proc. Natl. Acad. Sci. USA *108*, 8674–8679.

290. Brisson, L., Bański, P., Sboarina, M., Dethier, C., Danhier, P., Fontenille, M.-J., Van Hée, V.F., Vazeille, T., Tardy, M., Falces, J., et al. (2016). Lactate Dehydrogenase B Controls Lysosome Activity and Autophagy in Cancer. Cancer Cell *30*, 418–431.

291. Shi, L., Yan, H., An, S., Shen, M., Jia, W., Zhang, R., Zhao, L., Huang, G., and Liu, J. (2019). SIRT5-mediated deacetylation of LDHB promotes autophagy and tumorigenesis in colorectal cancer. Mol. Oncol. *13*, 358–375.

292. Yun, C.W., and Lee, S.H. (2018). The Roles of Autophagy in Cancer. Int. J. Mol. Sci. *19*, 3466.

Cell Reports Medicine, Volume *2*

Supplemental information

Metabolic adaptations in cancers expressing isocitrate dehydrogenase mutations

Ingvild Comfort Hvinden, Tom Cadoux-Hudson, Christopher J. Schofield, and James S.O. McCullagh

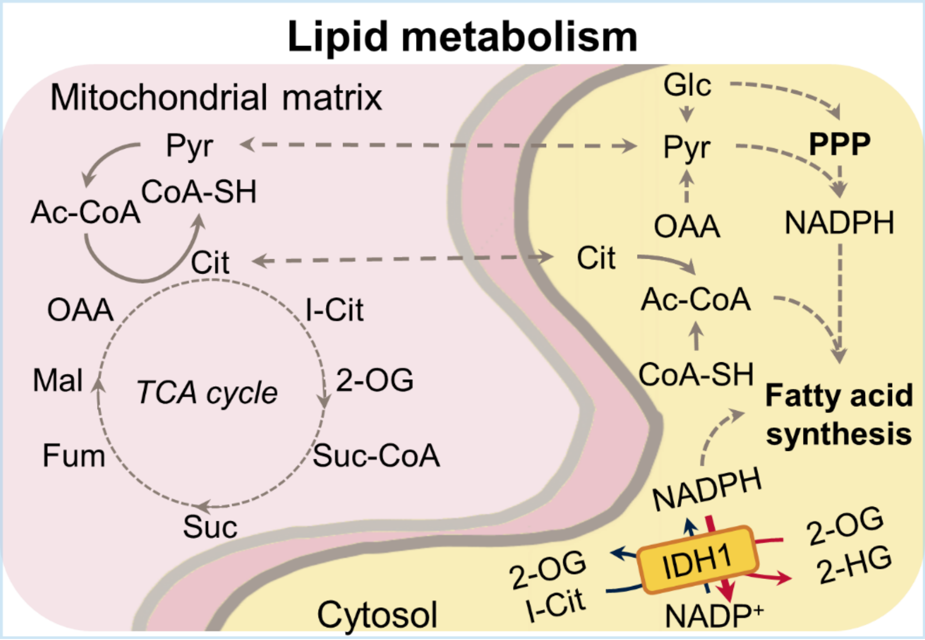

SI Figure 1. **Overview of IDH related lipid biosynthesis**. Lipid biosynthesis require NADPH, which is sourced from the oxidative pentose phosphate pathway (PPP), citric acid shuttle, and IDH1. When IDH1 is mutated, copious NADPH is used to synthesise 2-HG. Abbreviations: Cit (citrate), I-Cit (isocitrate), 2-OG (2-oxoglutarate), Suc-CoA (succinyl-CoA), Suc (succinate), Fum (fumarate), Mal (malate), OAA (oxaloacetate), Pyr (Pyruvate) and Ac-CoA (acetyl-CoA).
